# **Nemophilosides A**–**I, nine meroterpenoid glucosides isolated from *Nemophila menziesii***

**DOI:** 10.1007/s11418-025-01965-9

**Published:** 2025-12-29

**Authors:** Nanami Kurosawa, Yoshinobu Ishikawa, Tatsuo Katagiri, Eri Isowaki, Hayato Sato, Kenroh Sasaki, Toshihiro Murata

**Affiliations:** 1 Division of Pharmacognosy, Tohoku Medical and Pharmaceutical University, 4-1 Komatsushima 4-Chome Aoba-Ku, Sendai, 981-8558 Japan; 2 Faculty of Pharmaceutical Sciences, Shonan University of Medical Sciences, 16-10 Kamishinano, Totsuka-Ku, Yokohama, 244-0806 Japan; 3 Faculty of Liberal Arts and Sciences, University of Toyama, 2630, Sugitani, Toyama, 930-0194 Japan; 4 School of Pharmacy and Pharmaceutical Sciences, University of Toyama, 2630, Sugitani, Toyama, 930-0194, Japan

**Keywords:** Meroterpenoid glucoside, *Nemophila menziesii*, Boraginaceae, Nemophilosides A–I, NO production in RAW264.7 cells

## Abstract

Nine previously undescribed meroterpenoid glucosides, named nemophilosides A–I, were isolated from the whole plant extract of *Nemophila menziesii*. Their molecular structures were determined from their NMR and MS spectra, and the absolute configurations were confirmed using ECD spectroscopy by comparing the experimental data with calculated data. The meroterpenoids consist of monoterpenoids and hydroquinone moieties; however, meroterpenoids obtained as glycosides are relatively rare, which distinguishes this class of compounds. Their skeletons are similar to those of cannabinoids and shikonins, suggesting homologous biosynthetic pathways. Compared with the well-known meroterpenoids, the monoterpenoid moieties of the compounds isolated in this study were characterized by oxidation. Although these compounds showed little or no inhibitory activity against fatty acid amide hydrolase and acetylcholinesterase, compound **6** regulated NO production in RAW264.7 cells.

**Graphical Abstract:**

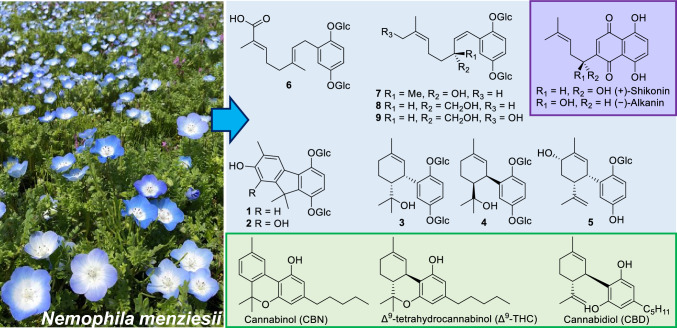

**Supplementary Information:**

The online version contains supplementary material available at 10.1007/s11418-025-01965-9.

## Introduction

*Nemophila menziesii* Hook. et Arn. (Boraginaceae), named baby blue eye, is an annual herb often used for ornamental purposes. Although native to North America, the blue flowers of the cultivar “Insignis Blue” are popular in gardens and parks worldwide. *Nemophila menziesii* is not commonly used for medicinal or culinary purposes despite several reports regarding the presence of a blue flower pigment [1]. In this study, we attempted to isolate meroterpenoids from the whole plant of *Nemophila menziesii.* Meroterpenoids are complexes of terpenoids and other biosynthetic products isolated from plants, fungi, and marine products, and exhibit diverse and potent biological activities; they are expected to show potential as pharmaceutical seeds, as many meroterpenoids, including cannabinoids and shikonins, have had a positive impact on society and are considered useful plant constituents [2]. The family Boraginaceae is recognized as one of the plant families that biosynthesize quinone-type meroterpenoids, with many characteristic meroterpenoids identified from the genus *Lithospermum*, [2] *Cordia*, [3] and *Arnebia*. [4] In this study, a series of significant meroterpenoids with quinone moiety, given their biosynthesis pathway, were isolated from the whole plant of *N. menziesii* (Insignis Blue). Notably, the meroterpenoids were isolated as glucoside types on the quinone. The procedures used to determine the chemical structure of meroterpenoids from this plant are described below.

## Results and discussion

**Fig. 1:**
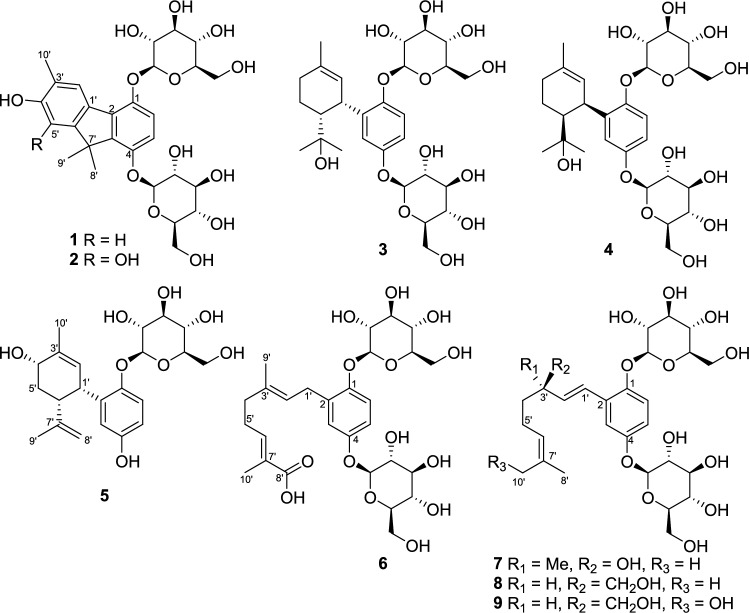
Chemical structures of meroterpenoids isolated from *Nemophila menziesii*

The acetone–water (4:1) extract (116 g) of the whole plants of *Nemophila menziesii* Hook. et Arn. was subjected to a Diaion HP-20 column to yield approximately separated fractions Frs. 1A-1G using a methanol–water mobile phase solvent system. Compounds **1**–**9** were isolated from Frs. 1C [MeOH: water (2:3)] and 1D [MeOH: water (3:2)] using reversed-phase HPLC. Compounds **1**–**9** comprised a hydroquinone, monoterpenoids, and either one or two glucosyl moieties (Fig. 1).

The HPLC sugar analyses after acid hydrolysis of **1**–**9** showed that the glucosyl moieties were D-glucose [5], and the coupling constant value *J* = 7.0–7.5 Hz indicated their β-orientations. That is, **1**–**9** were meroterpenoids characterized as di- or mono-glycosides according to the number of β-D-glucosyl moieties.

**Fig. 2:**
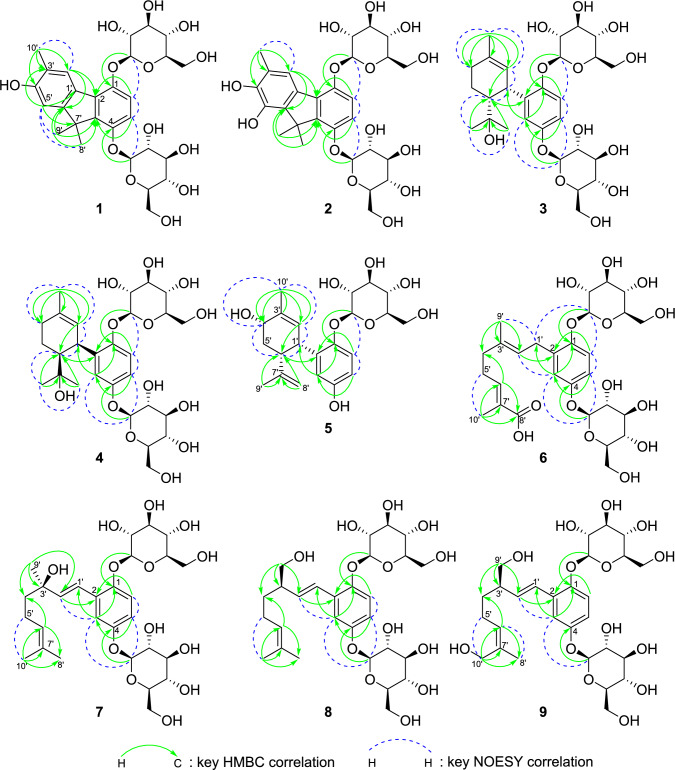
Key HMBC and NOE correlations of compounds **1**–**9**

The molecular formula of **1** (C_28_H_36_O_13_) was confirmed by (+)-HRFABMS ([M + Na]^+^ ion at *m*/*z* 603.2042, calculated for C_28_H_36_O_13_Na: 603.2053). The ^1^H and ^13^C NMR data for **1** are listed in Table 1. In the ^1^H NMR spectrum of **1**, three methyl [δ_H_ 2.24 (3H, s, H-10′), 1.56 (3H, s, H-9′), and 1.50 (3H, s, H-8′)], olefinic [δ_H_ 7.95 (1H, s, H-2′), 7.06 (1H, d, *J* = 9.0 Hz, H-6), 6.93 (1H, d,* J* = 9.0 Hz, H-5), and 6.77 (1H, s, H-5′) and glycosyl [δ_H_ 5.07–3.35] proton resonances were observed. In the aliphatic region of the ^13^C NMR spectrum of **1**, three methyl (δ_C_ 25.8, C-9′; 25.6, C-8′; 16.6, C-10′), one quaternary (δ_C_ 49.0, overlapping, C-7′), and two sets of glucosyl (δ_C_ 102.3, 75.3, 78.8, 71.5, 78.2, 62.6 and δ_C_ 102.1, 75.3, 78.7, 71.4, 78.1, 62.6) carbon resonances were observed. In its olefinic region, there were 12 carbon resonances indicating the presence of two phenyl moieties (δ_C_ 148.9, C-1; 131.5, C-2; 142.0, C-3; 151.1, C-4; 113.5, C-5; 115.9, C-6; 130.6, C-1′; 127.8, C-2′; 123.9, C-3′; 156.2, C-4′; 109.0, C-5′; 154.9, C-6′). In the HMBC spectrum, two methyl (H-8′ and H-9′) and H-5′ protons were long-range coupled with the quaternary C-7′; the methyl protons and H-5 were long-range coupled with C-3, which suggested the two phenyl moieties were connected to the quaternary carbon. The HMBC correlations from the other methyl and olefinic protons (Fig. 2) suggested the presence of the 3′,7′,7′-tri-methyl-1,4,4′-trioxy-fluorene skeleton. The NOESY correlation between H-2′ and H-10′ and that between H-5′ and H-8′ supported this conclusion. The anomeric protons at δ_H_ 5.07 (1H, d, *J* = 7.5 Hz) and 4.99 (1H, d, *J* = 7.5 Hz) were HMBC long-range coupled with C-1 and C-4, respectively, which showed the 1,4-diglucosyl of fluorene. From these data, the chemical structure of **1** was established as illustrated in Fig. 1.

**Table 1:** MR Spectroscopic Data for Compounds** 1–9**

**1**^*a*^					
				HMBC	NOESY
Position	*δ*_C_	type	*δ*_H_ (*J* in Hz)	(H to C)	(H to H)
1	148.9	C			
2	131.5	C			
3	142.0	C			
4	151.1	C			
5	113.5	CH	6.93, d (9.0)	1, 3, 7′	6, 4-*O*-Glc-1
6	115.9	CH	7.06, d (9.0)	2, 4	5, 1-*O*-Glc-1
1′	130.6	C			
2′	127.8	CH	7.95, s	2, 4′, 5′, 6′, 10′	10′
3′	123.9	C			
4′	156.2	C			
5′	109.0	CH	6.77, s	1′, 3′, 4′, 7′, 10′	8′, 9′
6′	154.9	C			
7′	49.0^*b*^	C			
8′	25.6	CH_3_	1.50, s	3, 6′, 7′, 9′	5′
9′	25.8	CH_3_	1.56, s	3, 6′, 7′, 8′	5′
10′	16.6	CH_3_	2.24, s	2′, 3′, 4′	2′
1-*O*-Glc-1	102.3	CH	5.07, d (7.5)	1	6, 1-*O*-Glc-2
1-*O*-Glc-2	75.3	CH	3.64, dd (9.0, 8.0)	1-*O*-Glc-1, 1-*O*-Glc-3	1-*O*-Glc-1
1-*O*-Glc-3	78.8	CH	3.35–3.55^*b*^		
1-*O*-Glc-4	71.5	CH	3.35–3.55^*b*^		
1-*O*-Glc-5	78.2	CH	3.35–3.55^*b*^		
1-*O*-Glc-6	62.6	CH_2_	3.71, dd (11.5, 5.0)	1-*O*-Glc-5	1-*O*-Glc-6
			3.92^*b*^	1-*O*-Glc-4	1-*O*-Glc-6
4-*O*-Glc-1	102.1	CH	4.99, d (7.5)	4	5
4-*O*-Glc-2	75.3	CH	3.35–3.55^*b*^		
4-*O*-Glc-3	78.7	CH	3.35–3.55^*b*^		
4-*O*-Glc-4	71.4	CH	3.35–3.55^*b*^		
4-*O*-Glc-5	78.1	CH	3.35–3.55^*b*^		
4-*O*-Glc-6	62.6	CH_2_	3.71, dd (11.5, 5.0)	4-*O*-Glc-5	4-*O*-Glc-6
			3.92^*b*^	4-*O*-Glc-4	4-*O*-Glc-6

**2**^*a*^					
				HMBC	NOESY
Position	*δ*_C_	type	*δ*_H_ (*J* in Hz)	(H to C)	(H to H)
1	149.1	C			
2	131.5	C			
3	143.0	C			
4	150.9	C			
5	113.6	CH	6.93, d (9.0)	1, 3	6, 4-*O*-Glc-1
6	115.6	CH	7.04, d (9.0)	2, 4	5, 1-*O*-Glc-1
1′ 2′	132.2	C			
	118.8	CH	7.55, s		
3′	125.2	C			
4′	143.7	C			
5′	143.0	C			
6′	138.3	C			
7′	49.6^*b*^	C			
8′	22.6	CH_3_	1.67, s	3, 6′, 7′, 9′	
9′	22.5	CH_3_	1.73, s	3, 6′, 7′, 8′	
10′	16.8	CH_3_	2.27, s	2′, 3′, 4′	
1-*O*-Glc-1	102.3	CH	5.07, d (7.5)	1	6
1-*O*-Glc-2	75.4	CH	3.63, dd (9.0, 8.0)	1-*O*-Glc-1, 1-*O*-Glc-3	
1-*O*-Glc-3	78.8	CH	3.44^*b*^		
1-*O*-Glc-4	71.6	CH	3.44^*b*^		
1-*O*-Glc-5	78.2	CH	3.44^*b*^		
1-*O*-Glc-6	62.7	CH_2_	3.69^*b*^		1-*O*-Glc-6
			3.89^*b*^	1-*O*-Glc-4	1-*O*-Glc-6
4-*O*-Glc-1	102.0	CH	4.99, d (7.5)	4	5
4-*O*-Glc-2	75.3	CH	3.54, dd (9.0, 7.5)	4-*O*-Glc-1, 4-*O*-Glc-3	
4-*O*-Glc-3	78.7	CH	3.44^*b*^		
4-*O*-Glc-4	71.4	CH	3.44^*b*^		
4-*O*-Glc-5	78.1	CH	3.44^*b*^		
4-*O*-Glc-6	62.6	CH_2_	3.69^*b*^		4-*O*-Glc-6
			3.89^*b*^	4-*O*-Glc-4	4-*O*-Glc-6

**3**^*a*^					
				HMBC	NOESY
Position	*δ*_C_	type	*δ*_H_ (*J* in Hz)	(H to C)	(H to H)
1	151.6	C			
2	136.2	C			
3	121.9	CH	6.99, d (3.0)	1, 5, 1′	2′, 4-*O*-Glc-1
4	154.4	C			
5	116.4	CH	6.94, dd (9.0, 3.0)	1, 3, 4	6, 4-*O*-Glc-1
6	118.5	CH	7.04, d (9.0)	1, 2, 4	5, 1-*O*-Glc-1
1′	34.3	CH	4.35, brt (4.5)	2, 2′, 3′	2′, 6′, 8′
2′	127.5	CH	5.39, brd (5.0)	4′, 6′, 10′	3, 1′, 10′
3′	134.1	C			
4′	32.4	CH_2_	2.20, m	3′, 6′	10′
5′	21.2	CH_2_	1.65–1.95^*b*^		
6′	52.0	CH	1.92, ddd (13.0, 4.5, 2.5)	2, 5′, 7′	1′, 8′, 9′
7′	74.3	C			
8′	25.1	CH_3_	0.69, s	6′, 7′, 9′	1′, 6′, 9′
9′	29.9	CH_3_	1.00, s	6′, 7′, 8′	5′, 6′, 8′
10′	23.6	CH_3_	1.72, s	2′, 3′, 4′	2′, 4′
1-*O*-Glc-1	104.3	CH	4.85, d (7.0)	1	6
1-*O*-Glc-2	74.9	CH	3.3–3.5^*b*^		
1-*O*-Glc-3	78.2	CH	3.3–3.5^*b*^		
1-*O*-Glc-4	71.3	CH	3.3–3.5^*b*^		
1-*O*-Glc-5	78.0	CH	3.3–3.5^*b*^		
1-*O*-Glc-6	62.6	CH_2_	3.74, dd (12.0, 5.0)		1-*O*-Glc-6
			3.85, brd (12.0)	1-*O*-Glc-4	1-*O*-Glc-6
4-*O*-Glc-1	103.3	CH	4.72, d (7.5)	4	3, 5
4-*O*-Glc-2	74.8	CH	3.3–3.5^*b*^		
4-*O*-Glc-3	78.1	CH	3.3–3.5^*b*^		
4-*O*-Glc-4	71.1	CH	3.3–3.5^*b*^		
4-*O*-Glc-5	78.0	CH	3.3–3.5^*b*^		
4-*O*-Glc-6	62.3	CH_2_	3.70, dd (12.0, 5.0)		4-*O*-Glc-6
			3.85, brd (12.0)	4-*O*-Glc-4	4-*O*-Glc-6

**4**^*a*^					
				HMBC	NOESY
Position	*δ*_C_	type	*δ*_H_ (*J* in Hz)	(H to C)	(H to H)
1	151.8	C			
2	134.8	C			
3	121.9	CH	6.97, d (3.0)	1, 4, 5, 1′	2′, 4-*O*-Glc-1
4	154.2	C			
5	116.5	CH	6.93, dd (9.0, 3.0)	3	6, 4-*O*-Glc-1
6	118.5	CH	7.22, d (9.0)	1, 2, 3, 4, 1′	5, 1-*O*-Glc-1
1′	34.3	CH	4.32, m	1, 2, 3, 2′	2′, 6′, 9′
2′	127.1	CH	5.35, brd, (5.5)	4′, 6′, 10′	3, 1′, 10′
3′	134.4	C			
4′	32.4	CH_2_	2.10–2.25^*b*^	2′, 5′, 6′	5′, 10′
5′	21.1	CH_2_	1.60–1.90^*b*^		4′
6′	51.9	CH	1.94, ddd (13.0, 4.5, 2.0)	2, 1′, 5′, 7′, 8′, 9′	1′, 8′, 9′
7′	74.4	C			
8′	28.7	CH_3_	0.94, s	6′, 7′, 9′	6′, 9′
9′	26.1	CH_3_	0.77, s	6′, 7′, 8′	1′, 6′, 8′
10′	23.6	CH_3_	1.73, s	2′, 3′, 4′	2′, 4′
1-*O*-Glc-1	104.4	CH	4.78, d (7.0)	1, 1-*O*-Glc-5	6
1-*O*-Glc-2	75.1	CH	3.39–3.45^*b*^		
1-*O*-Glc-3	78.4	CH	3.39–3.45^*b*^		
1-*O*-Glc-4	71.3	CH	3.39–3.45^*b*^		
1-*O*-Glc-5	78.1	CH	3.39–3.45^*b*^		
1-*O*-Glc-6	62.7	CH_2_	3.74, dd (12.0, 5.0)	1-*O*-Glc-5	
			3.70, dd (12.0, 5.0)	1-*O*-Glc-5	
4-*O*-Glc-1	102.6	CH	4.78, d (7.0)	4, 4-*O*-Glc-5	3
4-*O*-Glc-2	74.8	CH	3.39–3.45^*b*^		
4-*O*-Glc-3	78.3	CH	3.39–3.45^*b*^		
4-*O*-Glc-4	71.1	CH	3.39–3.45^*b*^		
4-*O*-Glc-5	78.0	CH	3.39–3.45^*b*^		
4-*O*-Glc-6	62.3	CH_2_	3.92, dd (12.0, 2.0)	4-*O*-Glc-5	
			3.84, dd (12.0, 2.0)	4-*O*-Glc-5	

**5**^*a*^					
				HMBC	NOESY
Position	*δ*_C_	type	*δ*_H_ (*J* in Hz)	(H to C)	(H to H)
1	150.7	C			
2	135.0	C			
3	117.9	CH	6.50, d (3.0)	1, 4, 5, 1′	
4	153.4	C			
5	114.7	CH	6.53, dd (8.5, 3.0)	1, 3, 4	6
6	120.4	CH	6.93, d (8.5)	1, 2, 4	5
1′	37.5	CH	4.38, brdd (5.5, 5.0)	1, 2, 3, 2′, 3′, 5′, 6′	2′, 6′, 9′
2′	130.5	CH	5.61, dd (5.0, 1.0)	1′, 4′, 6′, 10′	1′, 10′
3′	135.2	C			
4′	69.2	CH	4.12, brd (3.0)	2′, 3′, 6′	5′, 6′, 10′
5′	34.0	CH_2_	2.15, dt (14.0, 5.0)	1′, 6′	4′, 5′, 6′, 8′
			1.64^*b*^	1′, 3′, 4′, 6′	4′, 5′, 6′, 8′
6′	40.3	CH	2.80, ddd (13.5, 5.0, 2.5)	2, 1′, 4′, 5′, 7′, 8′, 9′	1′, 4′, 5′, 8′
7′	149.5	C			
8′	110.5	CH_2_	4.28, s	6′, 7′, 9′	5′, 6′, 8′, 9′
			4.50, s	6′, 7′, 9′	8′, 9′
9′	23.6	CH_3_	1.66, s	6′, 7′, 8′	1′, 8′
10′	21.4	CH_3_	1.87, s	2′, 3′, 4′	2′, 4′
1-*O*-Glc-1	105.6	CH	4.65, d (7.5)	1	6
1-*O*-Glc-2	75.2	CH	3.3–3.45^*b*^		
1-*O*-Glc-3	78.3	CH	3.3–3.45^*b*^		
1-*O*-Glc-4	71.7	CH	3.3–3.45^*b*^		
1-*O*-Glc-5	78.1	CH	3.25, m		
1-*O*-Glc-6	62.9	CH_2_	3.67, dd (12.0, 5.0)	1-*O*-Glc-5	1-*O*-Glc-6
			3.84, dd (12.0, 2.5)	1-*O*-Glc-4	1-*O*-Glc-6
4-*O*-Glc-1					
4-*O*-Glc-2					
4-*O*-Glc-3					
4-*O*-Glc-4					
4-*O*-Glc-5					
4-*O*-Glc-6					

**6**^*a*^					
				HMBC	NOESY
Position	*δ*_C_	type	*δ*_H_ (*J* in Hz)	(H to C)	(H to H)
1	152.2	C			
2	133.6	C			
3	119.1	CH	6.89, d (3.0)	1, 5, 1′	1′, 2′, 4-O-Glc-1
4	154.4	C			
5	115.7	CH	6.87, br s	1, 3	6, 4-*O*-Glc-1
6	117.7	CH	7.08, d (9.0)	1, 2, 3, 4, 5,	5, 1-*O*-Glc-1
1′	29.2	CH_2_	3.3–3.5^*b*^	1, 2, 3, 2′, 3′	3, 2′, 9′
2′	124.6	CH	5.39, dd (7.5, 6.0)	2, 1′, 4′, 9′	3, 1′, 4′
3′	136.4	C			
4′	39.4	CH_2_	2.18, br t (7.5)	2′, 3′, 5′, 6′, 9′	2′, 5′, 6′, 9′
5′	28.2	CH_2_	2.34, brdd (14.0, 7.5)	3′, 4′, 6′, 7′	4′, 6′, 9′, 10′
6′	143.7	CH	6.78, m	4′, 5′, 7′, 8′, 10′	4′, 5′
7′	128.9	C			
8′	171.7	C			
9′	16.2	CH_3_	1.74, s	2′, 3′, 4′	1′, 4′, 5′
10′	12.5	CH_3_	1.80, s	6′, 7′, 8′	5′
1-*O*-Glc-1	103.6	CH	4.79^*b*^	1	6
1-*O*-Glc-2	75.1	CH	3.3–3.5^*b*^		
1-*O*-Glc-3	78.3	CH	3.3–3.5^*b*^		
1-*O*-Glc-4	71.5	CH	3.3–3.5^*b*^		
1-*O*-Glc-5	78.1	CH	3.3–3.5^*b*^		
1-*O*-Glc-6	62.6	CH_2_	3.70, dd (12.0, 3.0)	1-*O*-Glc-5	1-*O*-Glc-6
			3.87, dd (12.0, 3.0)	1-*O*-Glc-4	1-*O*-Glc-6
4-*O*-Glc-1	103.0	CH	4.79^*b*^	4	3,5
4-*O*-Glc-2	74.9	CH	3.3–3.5^*b*^		
4-*O*-Glc-3	78.3	CH	3.3–3.5^*b*^		
4-*O*-Glc-4	71.4	CH	3.3–3.5^*b*^		
4-*O*-Glc-5	78.0	CH	3.3–3.5^*b*^		
4-*O*-Glc-6	62.5	CH_2_	3.70, dd (12.0, 3.0)	4-*O*-Glc-5	1-*O*-Glc-6
			3.87, dd (12.0, 3.0)	4-*O*-Glc-4	1-*O*-Glc-6

**7**^*a*^					
				HMBC	NOESY
Position	*δ*_C_	type	*δ*_H_ (*J* in Hz)	(H to C)	(H to H)
1	151.7	C			
2	130.0	C			
3	115.4	CH	7.27, d (3.0)	1, 4, 5, 1′	2′, 4-*O*-Glc-1
4	154.8	C			
5	117.9	CH	6.96, dd (9.0, 3.0)	1, 3, 4	6, 4-*O*-Glc-1
6	119.1	CH	7.13, d (9.0)	1, 2, 4, 5	5, 1-*O*-Glc-1
1′	122.6	CH	7.02, d (16.5)	1, 3, 3′	9′
2′	138.6	CH	6.27, d (16.5)	2, 3′, 9′	3, 4′, 9′
3′	74.2	C			
4′	44.0	CH_2_	1.60, t (6.0)	2′, 3′, 6′	2′, 5′, 6′, 9′
5′	24.0	CH_2_	2.06, m	4′, 6′, 7′	4′, 9′, 10′
6′	125.8	CH	5.13, dd (8.5, 7.0)	8′, 10′	4′, 8′, 10′
7′	132.2	C			
8′	25.9	CH_3_	1.66, s	6′, 7′, 10′	6′
9′	28.2	CH_3_	1.35, s	2′, 3′, 4′	1′, 2′, 4′, 5′
10′	17.8	CH_3_	1.60, s	6′, 7′, 8′	5′, 6′
1-*O*-Glc-1	103.9	CH	4.76, d (7.5)	1	6
1-*O*-Glc-2	75.1	CH	3.37–3.45^*b*^		
1-*O*-Glc-3	78.3	CH	3.37–3.45^*b*^		
1-*O*-Glc-4	71.4	CH	3.37–3.45^*b*^		
1-*O*-Glc-5	78.1	CH	3.37–3.45^*b*^		
1-*O*-Glc-6	62.6	CH_2_	3.69, dd (11.5, 5.0)	1-*O*-Glc-5	1-*O*-Glc-6
			3.88, m	1-*O*-Glc-4	1-*O*-Glc-6
4-*O*-Glc-1	103.4	CH	4.80, d (7.0)	4	3, 5
4-*O*-Glc-2	75.0	CH	3.37–3.45^*b*^		
4-*O*-Glc-3	78.2	CH	3.37–3.45^*b*^		
4-*O*-Glc-4	71.5	CH	3.37–3.45^*b*^		
4-*O*-Glc-5	78.1	CH	3.37–3.45^*b*^		
4-*O*-Glc-6	62.6	CH_2_	3.69, dd (11.5, 5.0)	4-*O*-Glc-5	4-*O*-Glc-6
			3.88, m	4-*O*-Glc-4	4-*O*-Glc-6

**8**^*a*^					
				HMBC	NOESY
Position	*δ*_C_	type	*δ*_H_ (*J* in Hz)	(H to C)	(H to H)
1	151.2	C			
2	130.2	C			
3	115.4	CH	7.29, d (3.0)	1, 4, 5, 1′	2′, 4-*O*-Glc-1
4	154.7	C			
5	117.7	CH	6.94, dd (9.0, 3.0)	1, 3, 4	6, 4-*O*-Glc-1
6	118.8	CH	7.10, d (9.0)	1, 2, 4, 5, 1′	5, 1-*O*-Glc-1
1′	127.0	CH	6.92, d (16.0)	1, 2, 3, 3′	3, 3′
2′	134.3	CH	6.03, dd (16.0, 9.0)	2, 3′, 4′, 9′	3, 3′, 4′, 9′
3′	47.1	CH	2.36, m		1′, 2′, 4′, 9′
4′	32.6	CH_2_	1.37, m	3′, 6′	2′, 4′, 5′
			1.55^*b*^	6′	3′, 4′, 5′, 9′, 10′
5′	26.8	CH_2_	2.01, m	3′, 4′, 6′, 7′	3′, 4′, 6′, 10′
6′	125.7	CH	5.13, t (8.0)	8′, 10′	5′, 8′
7′	132.5	C			
8′	26.0	CH_3_	1.68, s	6′, 7′, 10′	6′
9′	66.9	CH_2_	3.54, t (6.5)	2′, 3′, 4′	2′, 3′, 4′
10′	18.0	CH_3_	1.59, s	6′, 7′, 8′	4′, 5′
1-*O*-Glc-1	103.5	CH	4.79, d (7.0)	1, 1-*O*-Glc-5	6
1-*O*-Glc-2	75.1	CH	3.37–3.44^*b*^		
1-*O*-Glc-3	78.2	CH	3.37–3.44^*b*^		
1-*O*-Glc-4	71.5	CH	3.37–3.44^*b*^		
1-*O*-Glc-5	78.1	CH	3.37–3.44^*b*^		
1-*O*-Glc-6	62.6	CH_2_	3.69, dd (12.0, 5.0)	1-*O*-Glc-5	1-*O*-Glc-6
			3.87, dd (12.0, 2.0)	1-*O*-Glc-4	1-*O*-Glc-6
4-*O*-Glc-1	103.4	CH	4.79, d (7.0)	4, 4-*O*-Glc-5	3, 5
4-*O*-Glc-2	75.0	CH	3.37–3.44^*b*^		
4-*O*-Glc-3	78.2	CH	3.37–3.44^*b*^		
4-*O*-Glc-4	71.4	CH	3.37–3.44^*b*^		
4-*O*-Glc-5	78.1	CH	3.37–3.44^*b*^		
4-*O*-Glc-6	62.6	CH_2_	3.69, dd (12.0, 5.0)	4-*O*-Glc-5	4-*O*-Glc-6
			3.87, dd (12.0, 2.0)	4-*O*-Glc-4	4-*O*-Glc-6

**9**^*a*^					
				HMBC	NOESY
Position	*δ*_C_	type	*δ*_H_ (*J* in Hz)	(H to C)	(H to H)
1	151.2	C			
2	130.2	C			
3	115.3	CH	7.30, d (3.0)	1, 4, 5, 1′	2′, 4-*O*-Glc-1
4	154.7	C			
5	117.8	CH	6.94, dd (9.0, 3.0)	1, 2, 3, 4	6, 4-*O*-Glc-1
6	118.9	CH	7.11, dd (9.0)	1, 2, 4, 5, 1′	5, 1-*O*-Glc-1
1′	127.1	CH	6.87, d (18.0)	1, 2, 3, 4, 3′	3′
2′	134.1	CH	6.03, dd (16.0, 9.0)	2, 3′, 4′, 9′	3, 9′
3′	47.0	CH	2.35, m		1′, 4′, 9′
4′	32.7	CH_2_	1.39, m	3′, 6′	4′, 5′
			1.59, m		3′, 4′, 9′
5′	26.3	CH_2_	2.10, t (7.5)	4′, 6′, 7′	4′, 6′, 10′
6′	128.9	CH	5.29, t (6.0)	4′, 5′, 8′, 10′	4′, 5′, 8′
7′	136.0	C			
8′	21.6	CH_3_	1.76, s	6′, 7′, 10′	6′, 10′
9′	66.9	CH_2_	3.53, dd (6.0, 2.5)	2′, 3′, 4′	2′, 3′, 4′
10′	61.6	CH_2_	4.02, m	6′, 7′, 8′	5′, 8′
1-*O*-Glc-1	103.5	CH	4.80, d (7.5)	1, 1-*O*-Glc-5	6
1-*O*-Glc-2	75.1	CH	3.35–3.45^*b*^		
1-*O*-Glc-3	78.3	CH	3.35–3.45^*b*^		
1-*O*-Glc-4	71.6	CH	3.35–3.45^*b*^		
1-*O*-Glc-5	78.1	CH	3.35–3.45^*b*^		
1-*O*-Glc-6	62.6	CH_2_	3.68, dd (12.0, 5.5)		1-*O*-Glc-6
			3.85, dd (14.0, 3.0)		1-*O*-Glc-6
4-*O*-Glc-1	103.4	CH	4.80, d (7.5)	4, 4-*O*-Glc-5	3, 5
4-*O*-Glc-2	75.0	CH	3.35–3.45^*b*^		
4-*O*-Glc-3	78.2	CH	3.35–3.45^*b*^		
4-*O*-Glc-4	71.4	CH	3.35–3.45^*b*^		
4-*O*-Glc-5	78.0	CH	3.35–3.45^*b*^		
4-*O*-Glc-6	62.5	CH_2_	3.68, dd (12.0, 5.5)		4-*O*-Glc-6
			3.85, dd (14.0, 3.0)		4-*O*-Glc-6

^*a*^In methanol-*d*_4_ solution

^*b*^Unclear signal pattern due to overlapping

Compound **2** had a molecular formula of C_28_H_36_O_14_ (HRFABMS negative *m/z* 595.2042; [M–H]^–^ ion at *m*/*z*, calcd for C_28_H_35_O_14_: 595.2027), with one more oxygen atom than that in **1**. There is no H-5′ resonance in the ^1^H NMR spectrum, and an oxygenated olefinic carbon (δ_C_ 143.0, C-5′) was observed in the ^13^C NMR spectrum of **2**, instead of δ_C_ 109.0 in that of **1**. Therefore, **2** was confirmed to be a hydroxy derivative of **1** as shown in Fig. 1.

The molecular formula of **3** (C_28_H_42_O_13_) was confirmed using (+)-HRFABMS, which showed an [M + Na]^+^ ion at *m*/*z* 609.2515 (calcd. for C_28_H_42_O_13_Na: 609.2523). The ^1^H NMR spectrum of **3** showed three methyl [δ_H_ 1.72 (3H, s, H-10′), 1.00 (3H, s, H-9′), and 0.69 (3H, s, H-8′)], *o*- and *m*- coupling system olefinic [δ_H_ 6.99 (1H, d, *J* = 3.0 Hz, H-3), 6.94 (1H, dd, *J* = 9.0, 3.0 Hz, H-5), 7.04 (1H, d,* J* = 9.0 Hz, H-6)] proton resonances were observed. In the ^13^C NMR spectrum, 12 glycosidic, 10 terpenoid, and six phenyl carbon resonances were observed. These data, except for that of the sugar moieties were similar to those of conitriol which was one of the meroterpenoids isolated from the Ascidian *Aplidium conicum*. [6] The HMBC correlations (Fig. 2), including those from H-10′ to C-2′ (δ_C_ 127.5), C-3′ (δ_C_ 134.1), C-4′ (δ_C_ 32.4); from H-8′ and H-9′ to C-6′ (δ_C_ 52.0); from H-3 to C-1 (δ_C_ 151.6), C-5 (δ_C_ 116.4), C-1′ (δ_C_ 34.3), supported that the aglycone of **3** was conitriol.

**Fig. 3:**
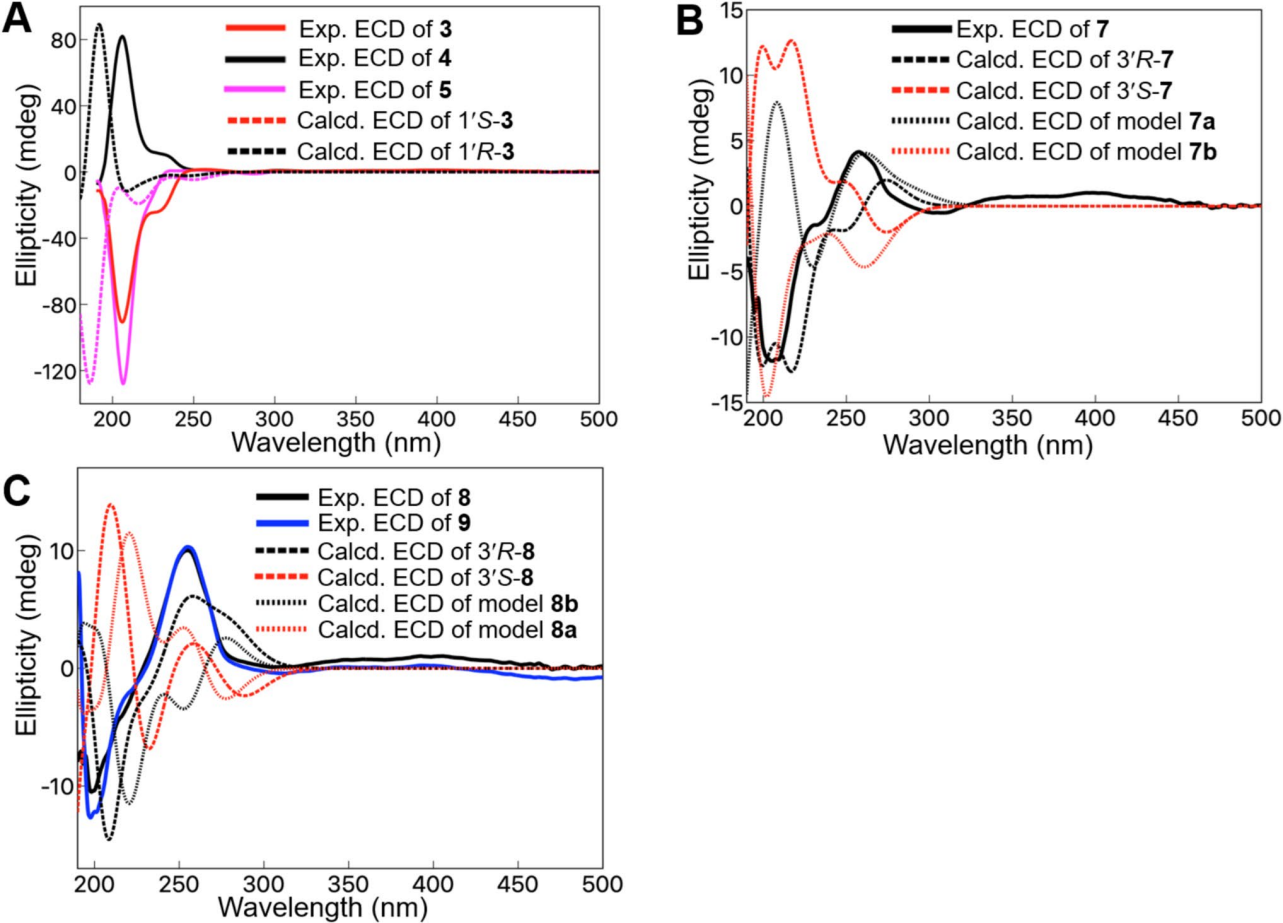
**A**: Experimental ECD spectra of **3**–**5** and calculated ECD spectra of 1′*S*-**3** and 1′*R*-**3**, **B**: Experimental ECD spectrum of **7** and calculated ECD spectra of 3′*R*-**7** and 3′*S*-**7** and simplified models **7a** and **7b**, **C**: Experimental ECD spectrum of **8** and **9** and calculated ECD spectra of 3′*R*-**8** and 3′*S*-**8** and simplified models **8a** and **8b**

The 1,4-diglucosyl moiety was confirmed by the HMBC correlations between the anomeric protons at δ_H_ 4.85 (1H, d, *J* = 7.0 Hz) and 4.72 (1H, d, *J* = 7.5 Hz) and C-1 and C-4 (δ_C_ 154.4), respectively. The relative configuration of C-1′ and C-6′ was confirmed as the *Z*-configuration by the coupling constant *J*_1′-6′_ = 4.5 Hz and the NOESY correlation, [6–8]. Based on the determined structures, the most stable conformations were calculated for all the possible absolute configurations at C-1′ and C-6′ on the cyclohexene ring (Figure S2). Thereafter, the dihedral diagonals were obtained and applied to the Karplus equation (1′*S*6′*R*: *J*_1′-6′_ = 4.37 Hz; 1′*S*6′*S*: *J*_1′-6′_ = 7.61 Hz; 1′*R*6′*R*: *J*_1′-6′_ = 1.56 Hz; 1′*R*6′*S*: *J*_1′-6′_ = 3.57 Hz), which supports its *Z*-configuration. Furthermore, their expected ECD spectra were calculated, and the C-1′ absolute configuration of **3** was confirmed to be *S* by the strong negative Cotton effect at approximately 205 nm in the ECD spectrum by comparison with the calculated data and experimental data of **3** (Fig. 3A). The chemical structure of compound **3** was confirmed to be as shown in Fig. 1.

The molecular formula of **4** (C_28_H_42_O_13_) was confirmed using (+)-HRFABMS ([M + Na]^+^ ion at *m*/*z* 609.2515, calcd. for C_28_H_42_O_13_Na: 609.2523), which was identical to that of **3**. The ^1^H and ^13^C NMR spectra of **4** are similar to those of **3** (Table 1), suggesting that the aglycone of **4** is an enantiomer of the aglycone of **3**. In the ECD curve of **4**, a positive Cotton effect was observed at approximately 205 nm, indicating the 1′*R*-configuration (Fig. 3A). Therefore, the structure of **4** was elucidated as shown in Fig. 1.

The molecular formula of **5** (C_22_H_30_O_8_) was determined using (–)-HRFABMS, which showed an [M–H]^–^ ion at *m*/*z* 421.1867 (calcd. for C_22_H_29_O_8_: 421.1863). The ^1^H and ^13^C NMR spectra of **5** (Table 1) were similar to those of **3** and **4**. Conversely, **5** had additional olefinic protons and carbons (δ_H_ 4.50, 1H, s, H-8′; 4.28, 1H, s, H-8′; δ_C_ 149.5, C-7′; 110.5, C-8′) and an oxygenated carbon and corresponding proton resonances (δ_H_ 4.12, 1H, br d, *J* = 3.0 Hz, H-4′; δ_C_ 69.2, C-4′) and only one set of the glucosyl moiety. In the HMBC spectrum, H-2′ (δ_H_ 5.61, 1H, dd, *J* = 5.0, 1.0 Hz), H-6′ (δ_H_ 2.80, 1H, ddd, *J* = 13.5, 5.0, 2.5), and H-10′ (δ_H_ 1.87, 3H, s) protons were long-range coupled with C-4′, suggesting a hydroxy group bonded to C-4′. The HMBC correlations from H-8′ to C-6′ (δ_C_ 40.3), C-7′, and C-9′ (δ_C_ 23.6) indicated the C-7′–C-8′ double bond. The anomeric proton resonance at δ_H_ 4.65 (1H, d, *J* = 7.5 Hz) was long-range coupled with C-1, suggesting that the glycosyl group was bonded to C-1. The relative and absolute configurations of C-1′*S* and C-6′*R* of **5** were confirmed by the same procedure used to confirm those of **3** and **4** (Fig. 3A and Figure S2). Although it was weak, a NOESY correlation was observed between H-4′ and H-6′, suggesting C-4′ was in the *R*-configuration. From these data, the structure of **5** was elucidated, as shown in Fig. 1.

The molecular formula of **6** (C_28_H_40_O_14_) was confirmed by (–)-HRFABMS, which showed a [M–H]^–^ ion at *m*/*z* 599.2323 (calcd. for C_28_H_39_O_14_: 599.2340). The IR absorption at 1683 cm^−1^ suggested the presence of the α,β-unsaturated carboxylic acid moiety. In the ^1^H NMR spectrum of **6**, the proton resonances of H-3 (δ_H_ 6.89, 1H, d, *J* = 3.0 Hz), H-5 (δ_H_ 6.87, 1H, br s), and H-6 (δ_H_ 7.08, 1H, d, *J* = 9.0 Hz) indicated the presence of the 1,2,4-trisubstituted benzene. The two 3H singlet proton resonances (δ_H_ 1.80 and 1.74) suggested the presence of two methyl groups. In the ^13^C NMR spectrum of **6**, a carbonyl (δ_C_ 171.7, C-8′), 10 olefinic (δ_C_ 152.2, C-1; 133.6, C-2; 119.1, C-3; 154.4, C-4; 115.7, C-5; 117.7, C-6; 124.6, C-2′; 136.4, C-3′; 143.7, C-6′; 128.9, C-7′), and 5 aliphatic (δ_C_ 29.2, C-1′; 39.4, C-4′; 28.2, C-5′; 16.2, C-9′; 12.5, C-10′) carbon resonances were observed. The HMBC correlations from H-3 to C-1, C-5, and C-1′, from H-6 to C-1, C-2, C-4, and C-5, from the two anomeric protons (δ_H_ 4.79, overlapping) to C-1 and C-4 indicated the 1,4-diglucosyl-2-*C*-substituted benzene moiety. Furthermore, the HMBC correlation from the two methyl groups (H-9′ to C-2′, C-3′, C-4′) and (H-10′ to C-6′, C-7′, C-8′) and from the two olefinic protons (H-2′ to C-2, C-1′, C-4′, C-9′) and (H-6′ to C-4′, C-5′, C-7′, C-8′, C-10′) established the structure of the side chain. The NOESY correlation between H-1′ and H-9′ and between H-5′ and H-10′ indicated a *E*-configurations of the C-2′–C-3′ and the C-6′–C-7′ double bonds. The data show the chemical structure of **6** (Fig. 1).

In the UV spectra of compounds **7**–**9**, common absorption peaks at 250–251 nm and 303–304 nm were observed, suggesting that they had similar skeletons. Compound **7** was a prenylated phenyl glycoside, similar to **6**. Its negative-mode HRFABMS (*m/z* 585.2535 [M–H]^–^, calcd for C_28_H_41_O_13_: 585.2547) established its molecular formula as C_28_H_42_O_13_. In the ^1^H NMR spectrum of **7**, one set of *E*-configured olefinic proton (δ_H_ 7.02, 1H, d, *J* = 16.5 Hz, H-1′; 6.27, 1H, d, *J* = 16.5 Hz, H-2′) and three singlet methyl proton (δ_H_ 1.66, H-8′; 1.60, H-10′; 1.35, H-9′) resonances were observed instead of those of H-1′ methylene, H-2′ olefinic, and two methyl protons (H-9′, H-10′) in **6**. The ^13^C NMR spectrum of **7** suggested that C-8′ was a methyl instead of a carbonyl as in **6**. Although it was difficult to determine the absolute configuration of C-3′ owing to its chain structure and low yield, we attempted to use ECD. The experimental spectrum was compared with the calculated values of the stable conformations of each 3′*R*-**7** and 3′*S*-**7**. The negative and positive Cotton effect curves of the calculated data in the 220–240 nm and 240–300 nm ranges, respectively, were in good agreement with the experimental curves (Fig. 3B). However, the 200–220 curves conflict with each other. Therefore, ECD of the simpler modeled structures of **7** (**7a** and **7b**) were calculated (Fig. 3B) and compared with the experimental ECD spectrum of **7**; the 3′*R* calculated curves were in better agreement with the experimental curve, suggesting a 3′*R* configuration of **7**. Therefore, 250–280 nm may be a key region. (Fig. 3B and Figure S3 and S4).

The molecular formula C_28_H_42_O_13_ of compound** 8** was identical to that of **7** as obtained from HRFABMS (negative) *m/z* 585.2540 [M–H]^–^ (calcd. for C_28_H_41_O_13_: 585.2547). In the ^1^H NMR spectrum of **8**, methylene (δ_H_ 3.54, 2H, t, *J* = 6.5 Hz, H-9′) and methine (δ_H_ 2.36, 1H, m, H-3′) proton resonances were observed instead of the H-9′ methyl in **7**. Similar to **7**, the stable conformations of 3′*R*-**8** and 3′*S*-**8** and their ECD data were calculated and compared with the experimental ECD spectrum of **8** (Fig. 3C). The 3′*R* calculated curves were in better agreement with the experimental curve, suggesting a 3′*R* configuration of **8**. Although the calculated data of the simpler modeled structures of **8** (**8a** and **8b**) were not fully conclusive, 240–270 nm and/or 190–210 nm appeared to be the key regions (Fig. 3C).

Compound **9** showed a molecular formula of C_28_H_42_O_14_ [HRFABMS (negative) *m/z* 601.2493 [M–H]^–^ (calcd. for C_28_H_41_O_14_: 601.2496), with one more oxygen atom than that of compound **8**. In the ^1^H NMR spectrum of **9**, an oxygenated methylene (δ_H_ 4.02, 2H, m, H-10′) proton resonance was observed instead of that of the H-10′ methyl as observed for **8**. The NOESY correlation between H-5′ and H-10′ and between H-6′ and H-8′ indicated a *Z*-configuration of the C-6′–C-7′ double bond. The experimental ECD spectrum of **9** was similar to that of **8**, indicating its 3′*R*-configuration (Fig. 3C). From the above results, the chemical structures of **7**–**9** were those shown in Fig. 1.

**Scheme 1:**
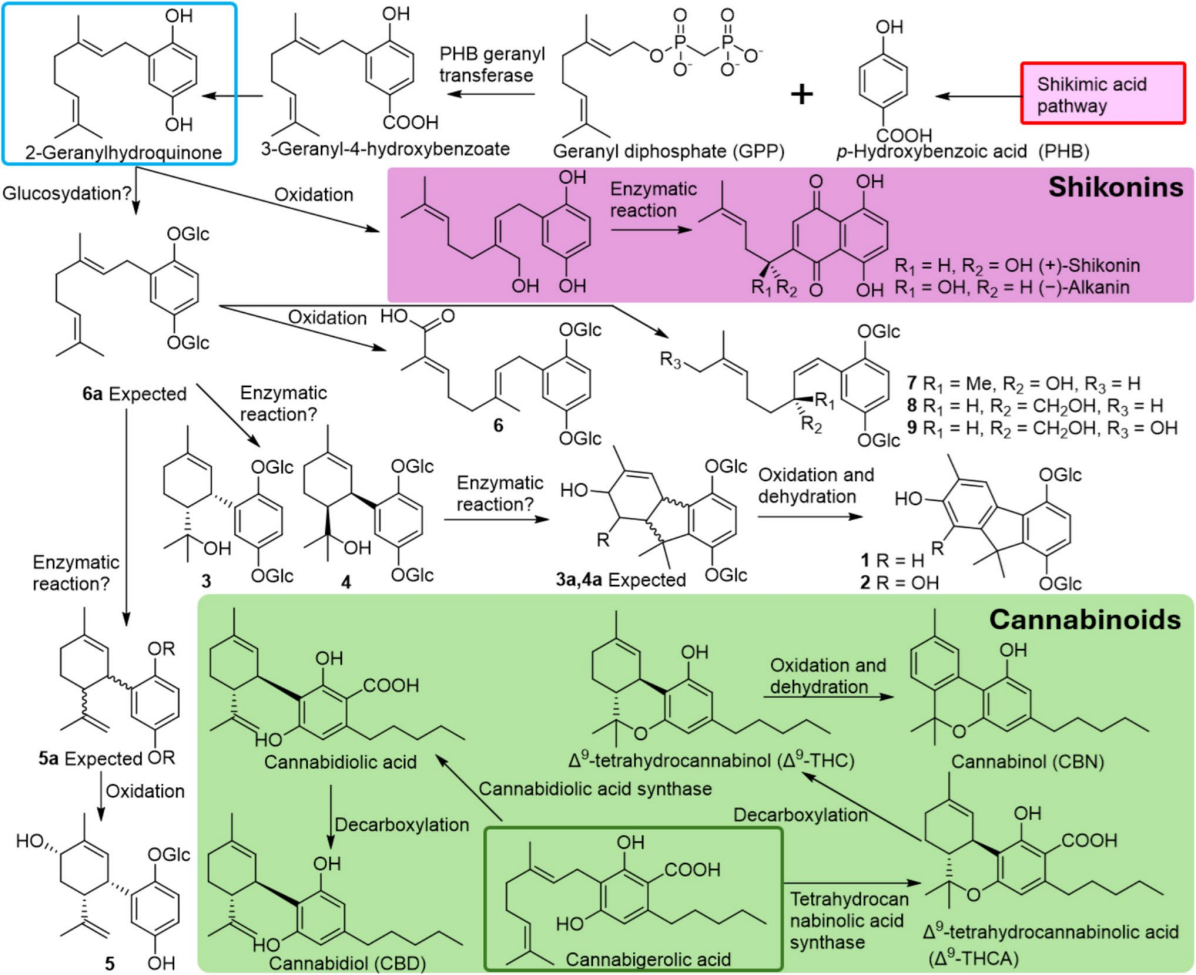
Expected biosynthetic pathway of *Nemophila* meroterpenoids compared with those of the shikonin of *Lithospermum erythrorhizon* and cannabinoids of *Cannabis sativa*

The meroterpenoids obtained in this study (**1**–**9**) are chemical structures characterized by being a hybrid of the hydroquinone moiety and the typical monoterpene moieties, including carvacrol (**1** and **2**), α-terpineol (**3** and **4**), carveol (**5**), and linalool (**7**). Compounds **6**–**9** have prenyl chain structures and are derivatives of geranylhydroquinone isolated from *Aplidium*; [6, 7] **6**–**9** are similar to the starting material for shikonins, which are the main constituents of the medicinal drug Lithospermum Root (*Lithospermum erythrorhizon*, Boraginaceae) [9]. Furthermore, compounds **3**–**5** may be applied in the order of the biosynthetic pathways of cannabinoids identified from *Cannabis sativa*, including tetrahydrocannabinol (THC) and cannabidiol (CBD) (Scheme 1). [10] Compounds **1** and **2** have a fluorene skeleton. Given their biosynthetic pathways, their structures are very similar to those of cannabinol (CBN). CBN has an ether bond with an olivetol moiety, and it appeared that in compounds** 1** and **2**, the bond between C-3 and C-7′ forms via a similar pathway (Scheme 1). Because CBN is easily produced via oxidation and dehydration from THC [10], it is possible that non-oxidized compounds (**3a**–**6a**) may exist in this plant. Although the specific mechanisms and enzyme involvement have not been established, compounds **1** and **2** are presumably derived from **3** and **4**, while compounds **6**–**9** are likely derived from **6a**, through a series of oxidation steps. The most important feature of *Nemophila* meroterpenoids is their glycosylated form, as such compounds are relatively rare. Second, many of them are oxidized compared with the general terpenoid moieties. Because glucosidation occurred only at the two hydroxyl groups of hydroquinone, it was assumed to be caused by hydroquinonization, and the absence of glycosylated hydroxy groups in all the obtained derivatives, except for the hydroquinone moieties, suggested that glucosidation occurred at the 2-geranylhydroquinone stage (Scheme 1).

The above (Scheme 1) provides hints for considering the biosynthetic pathways of plants that contain meroterpenoids and should be investigated in future studies.

**Fig. 4:**
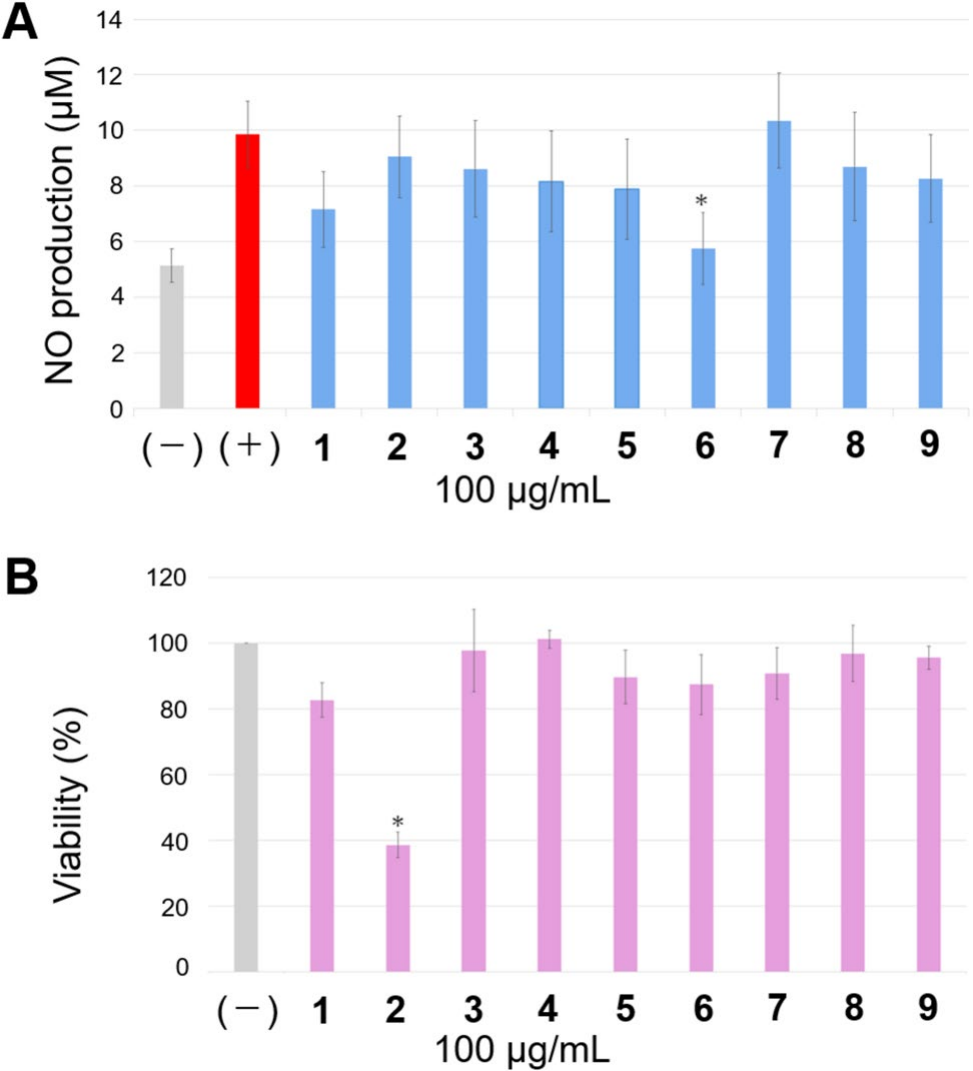
**A**: NO production from mouse RAW264.7 macrophage cells with the solutions of **1**–**9** and **B**: cytotoxicity of **1**–**9** against the cells. *: *p* < 0.05

To investigate the biological activities of the isolated meroterpenoids, with reference to the anti-inflammatory and/or sedative effects of shikonins [11] and cannabinoids [12] and the inhibitory activities against cholinesterase of many meroterpenoids [4], the anti-degranulation activity and toxicity to mouse RAW264.7 macrophage cells, fatty acid amide hydrolase (FAAH) inhibitory activity, and acetylcholinesterase (AChE) inhibitory activities were evaluated. Compound **6** significantly regulated nitric oxide (NO) production from the LPS-stimulated RAW264.7 cells at 100 µg/mL with no toxicity, showing inhibition of degranulation (Fig. 4).

The cytotoxicity of compound **2** may be attributed to the presence of catechol; however, the details are unknown. For the FAAH inhibitory test, the IC_50_ values were estimated as **1**, **2**, and **5**, and the compounds showed very low activity relative to that of the positive control JZL 195 hydrochloride (Table 2). No active compounds inhibited AChE in this study. Currently, among the *Nemophila* meroterpenoids, no significant biological activity has been revealed. One of the reasons why no strong activity was found in this study may be partly because **1**–**9** were all glycosides. The activities of aglycones alone or their function as glycosides in plants are issues to be addressed in future studies.

**Table 2:** In vitro FAAH inhibitory compounds from* Nemophila menziesii*

Sample	Inhibition rate (%)^***a***^	IC_50_ (mM)
**1**	93.7	5.41
**2**	92.1	3.98
**3**	26.9	N.D
**4**	36.2	N.D
**5**	72.0	12.3
**6**	34.6	N.D
**7**	23.8	N.D
**8**	30.2	N.D
**9**	1.81	N.D
positive control^***b***^	―	0.00388

Each value was average of triplicated tests

^***a***^1.0 mg of sample was solved in the 100 µL of DMSO

^***b***^JZL 195 hydrochloride

## Experimental section

### General experimental procedures

The optical rotations were recorded using a P-2300 polarimeter (Jasco Co., Tokyo, Japan). The UV spectra were recorded using a Shimadzu MPS-2450 instrument (Shimadzu, Kyoto, Japan). ECD spectra were recorded using a JASCO J-720 spectropolarimeter (Jasco Co.). Fluorescence detection assays were recorded using a SpectraMax® iD5 Multimode Microplate Reader (moleculardevices, Tokyo, Japan). ^1^H NMR (400 MHz) and ^13^C NMR (100 MHz), ^1^H-^1^H COSY, HMQC (optimized for ^1^*J*_C-H_= 145 Hz), and HMBC (optimized for ^n^*J*_C-H_= 8 Hz) spectra were recorded on JNM-AL400, JNM-ECZ400S/L1, and JNM-ECZ600R/S1 FT-NMR spectrometers (JEOL Ltd., Tokyo, Japan) (chemical shifts are expressed in *δ* relative to TMS (δ 0) as the internal standard or residual solvent peaks methanol-*d*_4_ (δ_H_ 3.305, δ_C_ 49.0). HR-FABMS data were obtained on a JMS700 mass spectrometer (Jeol Ltd.) using either *m*-nitrobenzyl alcohol or a glycerol matrix. A Diaion HP-20 column (Mitsubishi Chemical Co., Tokyo, Japan) was used for column chromatography. Preparative HPLC was performed on a Jasco 2089 instrument fitted with a UV detector (210 nm) [columns: TSKgel ODS-120T (Tosoh, Tokyo, Japan, 21.5 × 300 mm), TSK-gel ODS-80Ts (Tosoh, Tokyo, Japan, 21.5 × 300 mm), Develosil C_30_-UG-5 (Nomura Chemical, Aichi, Japan, 20 × 250 mm), CAPCELL PAK C18 AQ (OSAKA SODA CO., LTD., Osaka Japan, 20 × 250 mm), COSMOSIL 5C_18_-AR-II (NACALAI TESQUE, INC., Kyoto, Japan, 20 × 250 mm) and ODS-SM-50C-M (Yamazen Co., Osaka, Japan, 37 × 300 mm)].

### Plant material

*Nemophila menziesii* Hook. et Arn. (“Insignis Blue”) was harvested from the National Management Hitachi Seaside Park (605–4 Onuma-aza, Mawatari, Hitachinaka, Ibaraki 312–0012, Japan). A voucher specimen (no. TMPUNM20230530) was deposited in the herbarium of Tohoku Medical and Pharmaceutical University. One of the authors (TM) identified this plant species as previously described [13].

### Extraction and isolation

The whole plant of *Nemophila menziesii* was extracted using acetone-H_2_O (4:1) to obtain a crude extract (116.8 g). This crude extract was applied to a Diaion HP-20 open column, and eluted with H_2_O (fraction NM-1A, 68.0 g), methanol (MeOH)-H_2_O (1:4) (fraction NM-1B, 5.27 g), MeOH-H_2_O (2:3) (fraction NM-1C, 2.27 g), MeOH-H_2_O (3:2) (fraction NM-1D, 2.92 g), MeOH-H_2_O (4:1) (fraction NM-1E, 2.39 g), MeOH (fraction NM-1F, 1.5 g) and acetone (fraction NM-1G, 0.59 g). Fraction NM-1C was subjected to a reverse-phase HPLC column ODS-SM-50C-M and eluted using a gradient system from MeOH-H_2_O (1:9) to MeOH-H_2_O (1:1) to yield 14 fractions (fraction NM-2A–N). Fraction 2E (437.9 mg) was subjected to HPLC separation using TSK-gel ODS-80Ts (3:17 and 1:4 CH_3_CN-H_2_O as the mobile phases) to yield compounds **1** (6.0 mg) and **2** (6.8 mg). Fraction 2J (39.7 mg) was subjected to HPLC separation using a TSK-gel ODS-120T column (with 1:4 CH_3_CN-H_2_O as the mobile phase) to yield compound **3** (5.3 mg). Fraction 2F (252.4 mg) was subjected to HPLC separation using TSK-gel ODS-80Ts (1:9, 3:17, and 1:4 CH_3_CN-H_2_O as the mobile phase) and CAPCELL PAK C_18_ AQ (3:17 CH_3_CN-H_2_O as the mobile phase) to yield compound **9** (5.3 mg). Fraction NM-1D was subjected to HPLC separation using an ODS-SM-50C-M and eluted using a gradient system from MeOH-H_2_O (3:7) to MeOH-H_2_O (3:2), yielding 13 fractions (fraction NM-3A–M). Fraction 3B (231.7 mg) was subjected to HPLC separation using the TSK-gel ODS-80Ts (1:4 CH_3_CN-H_2_O as the mobile phase), and the COSMOSIL 5C_18_-AR-II (1:4 CH_3_CN-H_2_O as the mobile phase) to yield compound **5** (1.8 mg). A mixture of fractions 3C and 3D (280.5 mg) was subjected to HPLC separation using TSK-gel ODS-80Ts (3:17, CH_3_CN-H_2_O as the mobile phase) and CAPCELL PAK C_18_ AQ (1:4, CH_3_CN-H_2_O as the mobile phase) to yield compounds **3** (3.8 mg) and **4** (13.3 mg). A mixture of fractions 3E and 3F (311.6 mg) was subjected to HPLC separation using a TSK-gel ODS-80Ts (1:4, CH_3_CN-H_2_O as the mobile phase) and CAPCELL PAK C_18_ AQ (1:4 CH_3_CN-H_2_O as the mobile phase) to yield compound **6** (7.6 mg). A mixture of 3G, 3H, and 3I (321.1 mg) was subjected to HPLC separation using TSK-gel ODS-80Ts (5:15, CH_3_CN-H_2_O as the mobile phase), CAPCELL PAK C_18_ AQ (3:17, CH_3_CN-H_2_O as the mobile phase), and TSKgel ODS-120T (1:4, CH_3_CN-H_2_O as the mobile phase) to yield compounds **7** (6.1 mg) and **8** (2.0 mg).

#### Nemophiloside A (1)

Pale brown amorphous solid; [*α*]^21^_D_ –55 (*c* 5.6, MeOH); ^1^H NMR (MeOH-*d*_4_, 400 MHz), Table 1; ^13^C NMR (MeOH-*d*_4_, 100 MHz), Table 1; HRFABMS (positive) *m/z* 603.2042 [M + Na]^+^ (calcd for C_28_H_36_O_13_Na: 603.2053); IR *ν*max (KBr) cm^−1^: 3420, 2930, 1679, 1494, 1465, 1253, 1076; UV (MeOH) λmax (log ε) 219 (2.94), 278 (1.27), 301 (1.12) nm.

#### Nemophiloside B (2)

Dark brown amorphous solid; [*α*]^21^_D_ –83 (*c* 7.1, MeOH); ^1^H NMR (MeOH-*d*_4_, 400 MHz), Table 1; ^13^C NMR (MeOH-*d*_4_, 100 MHz), Table 1; HRFABMS (negative) *m/z* 595.2042 [M–H]^–^ (calcd for C_28_H_35_O_14_: 595.2027); IR *ν*max (KBr) cm^−1^: 3420, 2930, 1637, 1496, 1252, 1075; UV (MeOH) λmax (log ε) 222 (3.02), 282. (1.72) nm.

#### Nemophiloside C (3)

Pale brown amorphous solid; [*α*]^21^_D_ –136 (*c* 9.2, MeOH); ^1^H NMR (MeOH-*d*_4_, 400 MHz), Table 1; ^13^C NMR (MeOH-*d*_4_, 100 MHz), Table 1; HRFABMS (positive) *m/z* 609.2515 [M + Na]^+^ (calcd for C_28_H_42_O_13_Na: 609.2523); IR *ν*max (KBr) cm^−1^: 3393, 2932, 1648, 1493, 1386, 1245, 1201, 1075; UV (MeOH) λmax (log ε) 218 (2.81), 283 (0.76) nm; ECD (c 0.00050, MeOH) ([θ]) 206 (–106,400), 257 (+ 1800), 283 (–80), 302 (+ 900) nm.

#### Nemophiloside D (4)

Yellow amorphous solid; [*α*]^21^_D_ + 42 (*c* 10.3, MeOH); ^1^H NMR (MeOH-*d*_4_, 400 MHz), Table 1; ^13^C NMR (MeOH-*d*_4_, 100 MHz), Table 1; HRFABMS (positive) *m/z* 609.2515 [M + Na]^+^ (calcd for C_28_H_42_O_13_Na: 609.2523); IR *ν*max (KBr) cm^−1^: 3402, 2934, 1652, 1492, 1388, 1244, 1201, 1075; UV (MeOH) λmax (log ε) 219 (2.85), 282 (0.79), 323 (0.38) nm; ECD (c 0.00050, MeOH) ([θ]) 206 (+ 96,000), 280 (–400), 303 (+ 1200) nm.

#### Nemophiloside E (5)

Pale yellow amorphous solid; [*α*]^21^_D_ –153 (*c* 2.0, MeOH); ^1^H NMR (MeOH-*d*_4_, 400 MHz), Table 1; ^13^C NMR (MeOH-*d*_4_, 100 MHz), Table 1; HRFABMS (negative) *m/z* 421.1867 [M–H]^–^ (calcd for C_28_H_35_O_14_: 421.1863); IR *ν*max (KBr) cm^−1^: 3402, 2927, 1633, 1495, 1452, 1202, 1073, 1040; UV (MeOH) λmax (log ε) 206 (1.66), 287 (0.20) nm; ECD (c 0.00050, MeOH) ([θ]) 207 (–108,100), 257 (+ 900), 286 (–1000) nm.

#### Nemophiloside F (6)

Pale brown amorphous solid; [*α*]^21^_D_ –38 (*c* 4.9, MeOH); ^1^H NMR (MeOH-*d*_4_, 400 MHz), Table 1; ^13^C NMR (MeOH-*d*_4_, 100 MHz), Table 1; HRFABMS (negative) *m/z* 599.2323 [M–H]^–^ (calcd for C_28_H_39_O_14_: 599.2340); IR *ν*max (KBr) cm^−1^: 3401, 2926, 1683, 1496, 1386, 1206, 1074; UV (MeOH) λmax (log ε) 211 (2.43), 280 (0.37) nm.

#### Nemophiloside G (7)

Pale yellow amorphous solid; [*α*]^21^_D_ –47 (*c* 6.4, MeOH); ^1^H NMR (MeOH-*d*_4_, 400 MHz), Table 1; ^13^C NMR (MeOH-*d*_4_, 100 MHz), Table 1; HRFABMS (negative) *m/z* 585.2535 [M–H]^–^ (calcd for C_28_H_41_O_13_: 585.2547); IR *ν*max (KBr) cm^−1^: 3401, 2923, 1633, 1493, 1378, 1206, 1076; UV (MeOH) λmax (log ε) 217 (2.82), 250 (2.09), 303 (0.66) nm; ECD (c 0.00050, MeOH) ([θ]) 206 (–13,900), 258 (+ 4900), 308 (–10), 398 (+ 1200) nm.

#### Nemophiloside H (8)

Colorless amorphous solid; [*α*]^21^_D_ –23 (*c* 2.3, MeOH); ^1^H NMR (MeOH-*d*_4_, 400 MHz), Table 1; ^13^C NMR (MeOH-*d*_4_, 100 MHz), Table 1; HRFABMS (negative) *m/z* 585.2540 [M–H]^–^ (calcd for C_28_H_41_O_13_: 585.2547); IR *ν*max (KBr) cm^−1^: 3401, 2953, 2922, 1655, 1493, 1377, 1209, 1076; UV (MeOH) λmax (log ε) 210 (1.40), 251 (0.683), 304 (0.22) nm; ECD (c 0.00050, MeOH) ([θ]) 198 (–12,300), 255 (+ 11,700), 305 (+ 100), 405 (+ 1200) nm.

#### Nemophiloside I (9)

Colorless amorphous solid; [*α*]^21^_D_ –23 (*c* 5.3, MeOH); ^1^H NMR (MeOH-*d*_4_, 400 MHz), Table 1; ^13^C NMR (MeOH-*d*_4_, 100 MHz), Table 1; HRFABMS (negative) *m/z* 601.2493 [M–H]^–^ (calcd for C_28_H_41_O_14_: 601.2496); IR *ν*max (KBr) cm^−1^: 3393, 2927, 1672, 1493, 1430, 1208, 1076; UV (MeOH) λmax (log ε) 207 (0.26), 251 (0.11), 304 (0.03) nm; ECD (c 0.00050, MeOH) ([θ]) 198 (–15,300), 255 (+ 12,400), 313 (–500), 393 (+ 300) nm.

#### Acid hydrolysis and sugar identification

Compounds **1**–**4**,** 6**, **7**, and **9** (1.0 mg) and **5** and **8** (0.5 mg) were separately hydrolyzed with 6 N HCl (0.5 mL) at 70 °C for 1 h. The reaction mixture was filtered through an HP-20 column (5 × 50 mm) using H_2_O (2 mL), and the eluted solution was concentrated. The concentrated samples were separately stirred with L-cysteine methyl ester (5 mg, respectively) in pyridine (0.5 mL) at 60 °C for 1 h, after which *o*-tolyl isothiocyanate (10 μL) was added. The mixtures were analyzed using HPLC (Thermo Acclaim C_18_, 4.6 × 250 mm; mobile phase, CH_3_CN/H_2_O (1:3) containing 0.1% TFA, 1.0 mL/min; detector, UV at 256 nm). D-Glucose derivatives were detected at *t*_R_ 18.9–19.3 min by comparison with the authentic standards of D-glucose (*t*_R_ 18.9 min) and L-glucose derivatives (*t*_R_ 17.3 min), and the glycosidic moiety in **1**–**9** was identified as D-glucose [5].

#### ECD calculations

Conformational analysis was performed using a previously reported shell script [14]. More specifically, 300 energy-minimized three-dimensional structures of the stereoisomers of compounds **3**, **7**, and** 8**, and 150 energy-minimized three-dimensional structures of the simplified model structures of **7** (**7a** and **7b**) and **8** (**8a** and **8b**) were generated from the 2D chemical structures using Open Babel and Balloon [15, 16]. Every 25th conformer for **3** was geometrically optimized in MeOH using the conductor-like polarizable continuum model (CPCM) using the B3LYP/6–31 + G(d,p) level with Grimme dispersion corrections (GD3) [17]. The coupling constants between the vicinal protons at C-1′ and C-6′ on the cyclohexene ring of the lowest-energy conformers of 1′*S*-**3** and 1′*R*-**3** were predicted by the Karplus equation. [18] Every 25th conformer for **7** and **8** was geometrically optimized in the gas phase using the B3LYP/6–31 + G(d,p) level of theory. Every 25th conformer for **7a**, **7b**, **8a**, and **8b** was geometrically optimized in the gas phase using the B3LYP/Def2TZVP level of theory. The ECD calculations for 1′*S*-**3** and 1′*R*-**3** were conducted at the CAM-B3LYP/TZVP level of time-dependent density functional theory (TDDFT) in MeOH using CPCM. The ECD calculations for 3′*R*-**7**, 3′*S*-**7**, 3′*R*-**8**, and 3′*S*-**8** were conducted at the B3LYP/6–31 + G(d,p) level of TDDFT in MeOH using CPCM. ECD calculations for **7a**, **7b**, **8a**, and **8b** were conducted at the B3LYP/TZVP level of TDDFT in MeOH using CPCM. All calculations were performed using Gaussian 16 [19]. The ECD spectra were obtained from 45 calculated excitation energies and rotational strengths as the sum of the Gaussian functions centered at the wavelength of each transition with parameter *s*, which represents the width of the band at a half-height of 0.30 eV.

### Cytotoxicity against RAW264.7

The RAW 264.7 murine macrophage cell line was obtained from the American Type Culture Collection (ATCC TIB71, VA, USA). The cells were grown at 37 ℃ in DMEM medium (FUJIFILM Wako Pure Chemical Corporation, Ltd., Osaka, Japan.) supplemented with 10% FBS (NICHIREI BIOSCIENCES INC., Tokyo, Japan), penicillin (100 units/mL), and streptomycin sulfate (100 mg/mL).

The cytotoxicity of the isolated compounds (**1**–**9**) was estimated using a cell counting kit-8 (Dojindo Laboratories, Kumamoto, Japan) to count living cells by combining 2-(2-methoxy-4-nitrophenyl)-3-(4-nitrophenyl)-5-(2,4-disulfophenyl)- 2H-tetrazolium (WST-8) with 1-methoxyphenazinemethosulfate (1-methoxy-PMS). RAW264.7 cells were dispensed in 100 μL cell suspensions (5 × 10^3^ cells/well) onto a 96-well plate and preincubated for 2 h in a humidified incubator (37 ℃, 5% CO_2_). Cells were treated with the isolated compounds (**1**–**9**) (100 μg/mL) in PBS and compared with a nontreated control group (*n* = 3 wells per group). The treated cells were incubated for 24 h, CCK-8 was added to each well, and the cells were cultured for an additional 2 h. The absorbance of each well was measured at 450 nm using a multi-plate reader (FilterMax F5; Molecular Devices, CA, USA). The ratio of the mean absorbance value for each group to that of the control group was used to determine cell viability.

*Inhibitory effects on NO production in LPS-stimulated RAW264.7 cells.* RAW 264.7 macrophages were plated at 6.0 × 10^5^ cells/0.5 mL in 24 plates and then incubated with or without LPS (1 μg/mL) in the absence or presence of the isolated compounds (**1**–**9**) for 24 h. Nitrite accumulation in the culture medium was measured as an indicator of NO production, based on the Griess reaction. Briefly, 80 μL of cell culture medium was mixed with 80 μL of Griess reagent [equal volumes of % (w/v) sulfanilamide in 5% (v/v) phosphoric acid and 0.1% (w/v) naphtylethylenediamine-HCl], incubated at room temperature for 10 min; thereafter, the absorbance was measured at 540 nm in a microplate reader (FilterMax F5). Fresh culture medium was used as the blank for all experiments. The amount of nitrite in each sample was measured using a serially diluted sodium nitrite standard curve.

### FAAH inhibitory activity

The FAAH inhibitory activities of all the isolated compounds (**1**–**9**) were evaluated using a Fatty Acid Amide Hydrolase Inhibitor Screening Assay Kit (Cayman Chemical Company, Michigan, USA). Positive control (JZL195) concentrations were 20 µM, 10 µM, 2 µM, 0.2 µM, 0.02 µM, and 0.002 µM. The inhibitory activity of each compound (10 mg/mL in DMSO at the time of preparation) was tested using a screening approach. For those showing more than 50% inhibition, diluted samples (1 mg/mL at the time of preparation, dissolved in DMSO, and 0.1 mg/mL at the time of preparation, dissolved in DMSO) were tested and the IC_50_ value was calculated between the two points when 50% inhibition was between the two points.

In a 96-well plate, 170 µL of 125 mM Tris–HCl containing 1 mM EDTA buffer (pH 9.0), 10 µL of FAAH (human recombinant) solution, and 10 µL of sample solution were added and mixed. Blank wells comprised 180 µL of buffer and 10 µL of DMSO instead of the sample; the positive control wells comprised 10 µL of JZL195 (20 µM at the time of preparation, dissolved in DMSO) instead of the sample, and background wells comprised 10 µL of DMSO instead of FAAH solution. The mixture was incubated at 37 °C for 5 min. Subsequently, 10 µL of FAAH substrate (AMC arachidonoyl amido, 20 µM at the time of preparation, dissolved in EtOH) was added to each well. The wells were incubated again at 37 °C for 30 min. The fluorescence was measured at room temperature, after incubation, using a SpectraMax® iD5 Multimode Microplate Reader (moleculardevices, Tokyo, Japan) at an excitation wavelength of 350 nm and emission wavelength of 460 nm. The inhibition rate was calculated using the following equation: Inhibition rate (%) = [1 – {(A _sample_) – (A _background sample_)} / {(A _blank_) – (A _background blank_)}] × 100.

## Supplementary Information

Below is the link to the electronic supplementary material.

Supplementary file1 (PDF 8428 KB)
