# Supplementary material for: Nemophilosides A–I, nine meroterpenoid glucosides isolated from Nemophila menziesii
Source: J Nat Med. 2025 Dec 29;80(1):91–109. doi: 10.1007/s11418-025-01965-9 (PMC12847176; doi:10.1007/s11418-025-01965-9)
Supplement: Supplementary file 1 — Supplementary file1 (PDF 8428 KB) [file 11418_2025_1965_MOESM1_ESM.pdf]

Supplementary Information for **Nemophilosides A–I, Nine Meroterpenoid Glucosides Isolated from *Nemophila menziesii***

Nanami Kurosawa, Yoshinobu Ishikawa, Tatsuo Katagiri, Eri Isowaki, Hayato Sato,

Kenroh Sasaki, Toshihiro Murata*

*Corresponding author.
Dr. MURATA Toshihiro
Tel: +81 22 727 0086. Fax: +81 22 727 0220.
E-mail address: murata-t@tohoku-mpu.ac.jp

Table of contents

Figure S1 Flowers of *Nemophila menziesii* Hook. et Arn. ・・・・・・・・・・5
Figure S2 Stable conformations of stereo isomers of compound **3** (**A**) and simplified models (**B**) and their calculated $J_{1'\text{-}6'}$ values・・・・・・・6
Figure S3 Stable conformations of stereo isomers of compounds **7** and **8**・・7
Figure S4 Conformations of simplified models of compounds **7** (**7a** and **7b**) and **8** (**8a** and **8b**) ・・・・・・・・・・・・・・・・・・・・・・・8
Figure S5 ^{1}H NMR spectrum of compound **1** (in methanol-d_4)・・・・・・・9
Figure S6 ^{13}C NMR spectrum of compound **1** (in methanol-d_4) ・・・・・・10
Figure S7 DEPT spectrum of compound **1** (in methanol-d_4) ・・・・・・・・11
Figure S8 ^{1}H-^{1}H COSY spectrum of compound **1** (in methanol-d_4) ・・・・・12
Figure S9 HMQC spectrum of compound **1** (in methanol-d_4) ・・・・・・・13
Figure S10 HMBC spectra of compound **1** (in methanol-d_4)・・・・・・・・14
Figure S11 NOESY spectra of compound **1** (in methanol-d_4)・・・・・・・・15
Figure S12 ^{1}H NMR spectrum of compound **2** (in methanol-d_4)・・・・・・・16
Figure S13 ^{13}C NMR spectrum of compound **2** (in methanol-d_4) ・・・・・・17
Figure S14 DEPT spectrum of compound **2** (in methanol-d_4)・・・・・・・・18
Figure S15 ^{1}H-^{1}H COSY spectrum of compound **2** (in methanol-d_4) ・・・・19
Figure S16 HMQC spectrum of compound **2** (in methanol-d_4)・・・・・・・20
Figure S17 HMBC spectra of compound **2** (in methanol-d_4)・・・・・・・・21
Figure S18 NOESY spectra of compound **2** (in methanol-d_4)・・・・・・・・21
Figure S19 ^{1}H NMR spectrum of compound **3** (in methanol-d_4)・・・・・・・22
Figure S20 ^{13}C NMR spectrum of compound **3** (in methanol-d_4)・・・・・・23
Figure S21 DEPT spectrum of compounds **3** (in methanol-d_4)・・・・・・・24
Figure S22 ^{1}H-^{1}H COSY spectrum of compound **3** (in methanol-d_4)・・・・・25
Figure S23 HMQC spectrum of compound **3** (in methanol-d_4)・・・・・・・25
Figure S24 HMBC spectrum of compound **3** (in methanol-d_4)・・・・・・・26
Figure S25 NOESY spectrum of compound **3** (in methanol-d_4)・・・・・・・26
Figure S26 ^{1}H NMR spectrum of compound **4** (in methanol-d_4)・・・・・・・27
Figure S27 ^{13}C NMR spectrum of compound **4** (in methanol-d_4)・・・・・・28
Figure S28 DEPT spectrum of compound **4** (in methanol-d_4)・・・・・・・29
Figure S29 ^{1}H-^{1}H COSY spectrum of compound **4** (in methanol-d_4)・・・・・30
Figure S30 HMQC spectrum of compound **4** (in methanol-d_4)・・・・・・・30
Figure S31 HMBC spectrum of compound **4** (in methanol-d_4)・・・・・・・31
Figure S32 NOESY spectrum of compound **4** (in methanol-d_4)・・・・・・・31
Figure S33 ^{1}H NMR spectrum of compound **5** (in methanol-d_4)・・・・・・・32
Figure S34 ^{13}C NMR spectrum of compound **5** (in methanol-d_4)・・・・・・33
Figure S35 DEPT spectrum of compound **5** (in methanol-d_4)・・・・・・・34
Figure S36 ^{1}H-^{1}H COSY spectrum of compound **5** (in methanol-d_4)・・・・・35

Figure S37 HMQC spectrum of compound **5** (in methanol-d_4) · · · · · · 35
Figure S38 HMBC spectrum of compound **5** (in methanol-d_4) · · · · · · · 36
Figure S39 NOESY spectrum of compound **5** (in methanol-d_4) · · · · · · · 36
Figure S40 ^{1}H NMR spectrum of compound **6** (in methanol-d_4) · · · · · · · 37
Figure S41 ^{13}C NMR spectrum of compound **6** (in methanol-d_4) · · · · · · 38
Figure S42 DEPT spectrum of compound **6** (in methanol-d_4) · · · · · · · 39
Figure S43 ^{1}H-^{1}H COSY spectrum of compound **6** (in methanol-d_4) · · · · · 39
Figure S44 HMQC spectrum of compound **6** (in methanol-d_4) · · · · · · 40
Figure S45 HMBC spectrum of compound **6** (in methanol-d_4) · · · · · · · 40
Figure S46 NOESY spectrum of compound **6** (in methanol-d_4) · · · · · · · 41
Figure S47 ^{1}H NMR spectrum of compound **7** (in methanol-d_4) · · · · · · · 42
Figure S48 ^{13}C NMR spectrum of compound **7** (in methanol-d_4) · · · · · · 43
Figure S49 DEPT spectrum of compound **7** (in methanol-d_4) · · · · · · · 44
Figure S50 ^{1}H-^{1}H COSY spectrum of compound **7** (in methanol-d_4) · · · · · 45
Figure S51 HMQC spectrum of compound **7** (in methanol-d_4) · · · · · · · 45
Figure S52 HMBC spectrum of compound **7** (in methanol-d_4) · · · · · · · 46
Figure S53 NOESY spectrum of compound **7** (in methanol-d_4) · · · · · · · 46
Figure S54 ^{1}H NMR spectrum of compound **8** (in methanol-d_4) · · · · · · · 47
Figure S55 ^{13}C NMR spectrum of compound **8** (in methanol-d_4) · · · · · · 48
Figure S56 DEPT spectrum of compound **8** (in methanol-d_4) · · · · · · · 49
Figure S57 ^{1}H-^{1}H COSY spectrum of compound **8** (in methanol-d_4) · · · · · 49
Figure S58 HMQC spectrum of compound **8** (in methanol-d_4) · · · · · · 50
Figure S59 HMBC spectrum of compound **8** (in methanol-d_4) · · · · · · · 50
Figure S60 NOESY spectrum of compound **8** (in methanol-d_4) · · · · · · · 51
Figure S61 ^{1}H NMR spectrum of compound **9** (in methanol-d_4) · · · · · · · 52
Figure S62 ^{13}C NMR spectrum of compound **9** (in methanol-d_4) · · · · · · 53
Figure S63 DEPT spectrum of compound **9** (in methanol-d_4) · · · · · · · 54
Figure S64 ^{1}H-^{1}H COSY spectrum of compound **9** (in methanol-d_4) · · · · · 55
Figure S65 HMQC spectrum of compound **9** (in methanol-d_4) · · · · · · · 55
Figure S66 HMBC spectrum of compound **9** (in methanol-d_4) · · · · · · · 56
Figure S67 NOESY spectrum of compound **9** (in methanol-d_4) · · · · · · · 56
Figure S68 HRFABMS of compound **1** · · · · · · · · · · · · · · · · · 57
Figure S69 HRFABMS of compound **2** · · · · · · · · · · · · · · · · · 57
Figure S70 HRFABMS of compound **3** · · · · · · · · · · · · · · · · · 58
Figure S71 HRFABMS of compound **4** · · · · · · · · · · · · · · · · · 58
Figure S72 HRFABMS of compound **5** · · · · · · · · · · · · · · · · · 59
Figure S73 HRFABMS of compound **6** · · · · · · · · · · · · · · · · · 59
Figure S74 HRFABMS of compound **7** · · · · · · · · · · · · · · · · · 60
Figure S75 HRFABMS of compound **8** · · · · · · · · · · · · · · · · · 60
Figure S76 HRFABMS of compound **9** · · · · · · · · · · · · · · · · · 61
Figure S77 HPLC analyses for sugar identification of **1-4** · · · · · · · · 61

Figure S78 HPLC analyses for sugar identification of **5-8** · · · · · · · · · 62
Figure S79 HPLC analyses for sugar identification of **9** · · · · · · · · · · 62

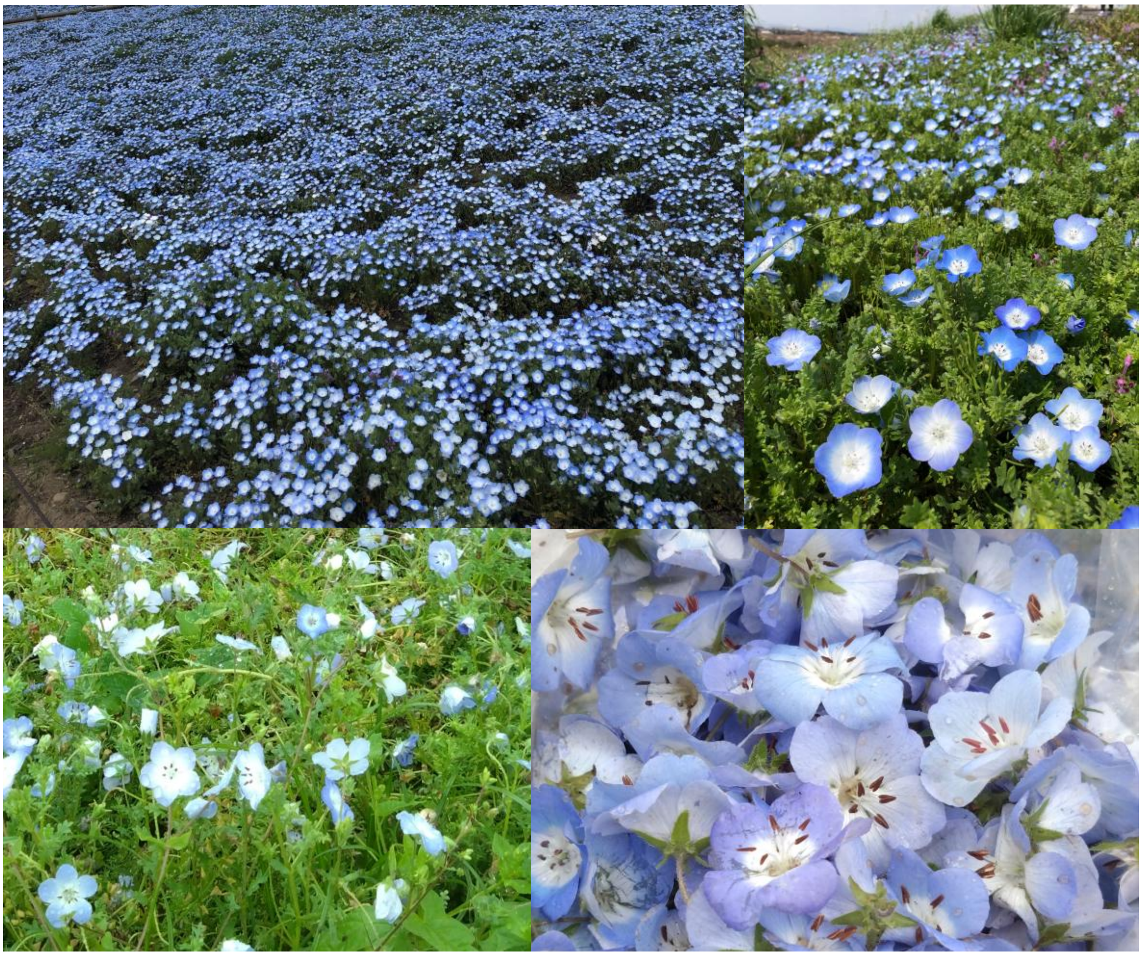

Figure S1 Flowers of *Nemophila menziesii* Hook. et Arn.

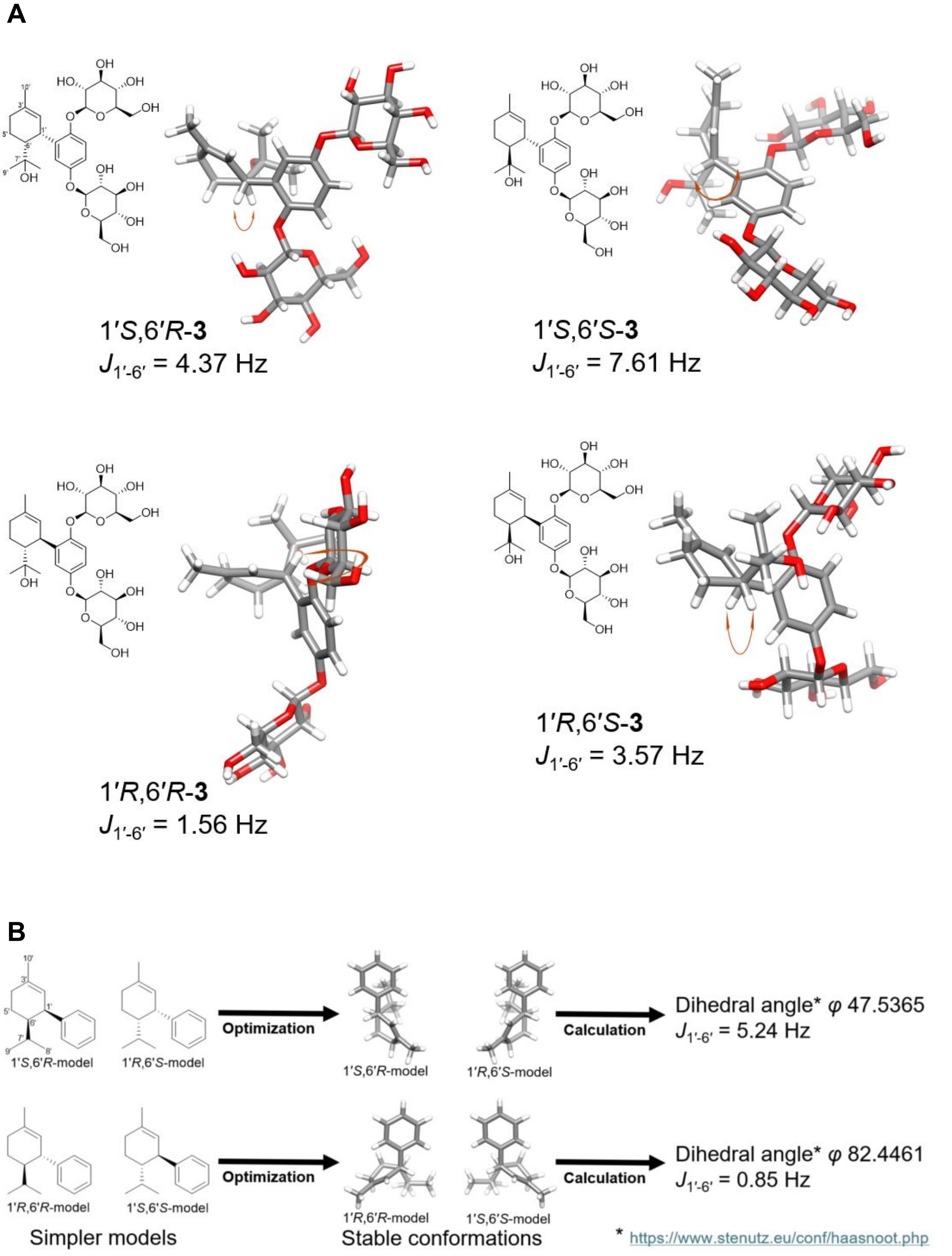

Figure S2 Stable conformations of stereo isomers of compound **3** (**A**) and simplified models (**B**) and their calculated $J_{1'-6'}$ values

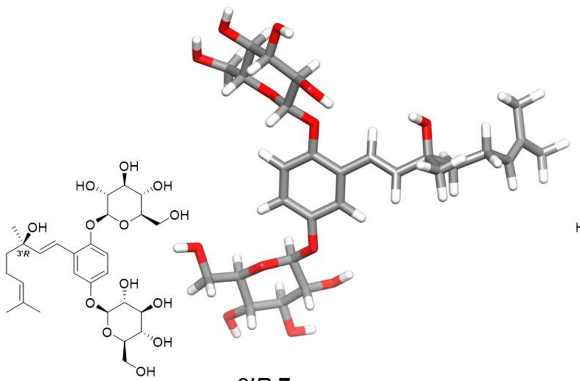

3′*R*-**7**

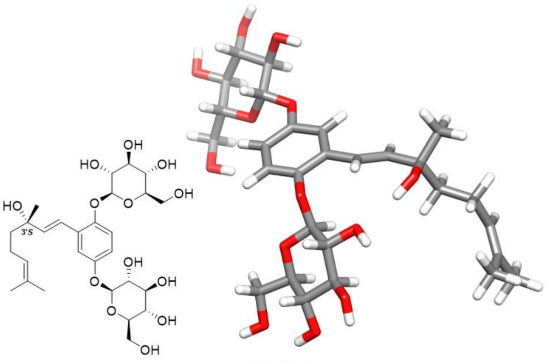

3′*S*-**7**

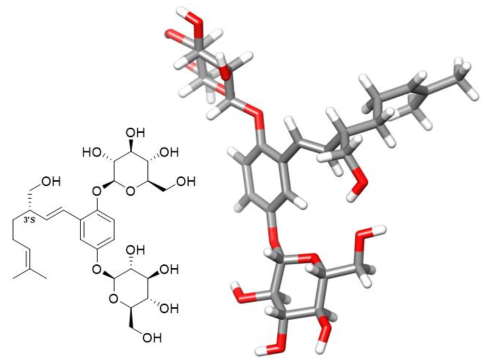

3′*S*-**8**

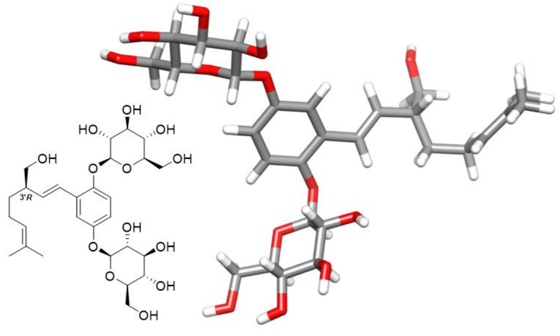

3′*R*-**8**

Figure S3 Stable conformations of stereo isomers of compounds **7** and **8**

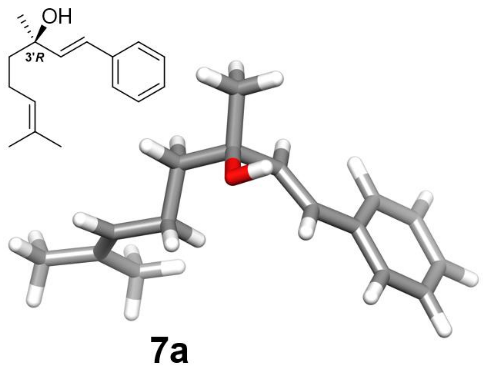

7a

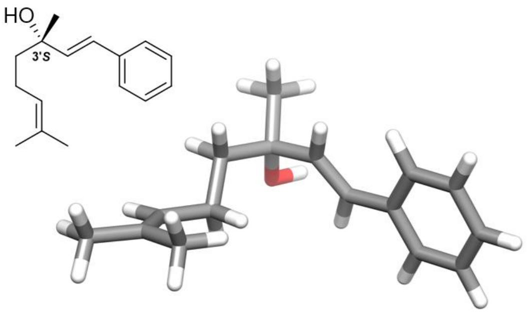

7b

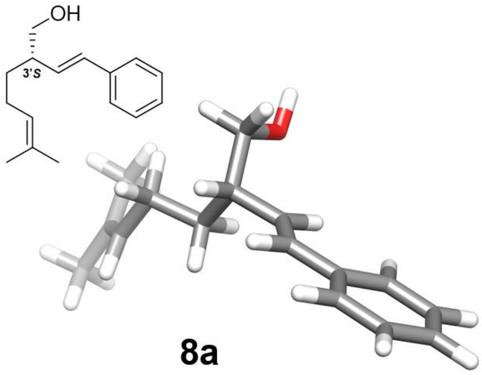

8a

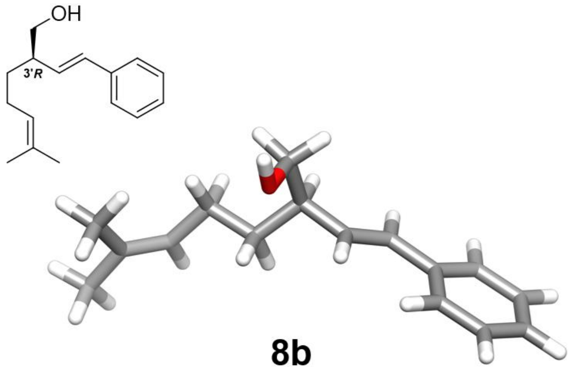

8b

Figure S4 Conformations of simplified models of compounds **7** (**7a** and **7b**) and **8** (**8a** and **8b**).

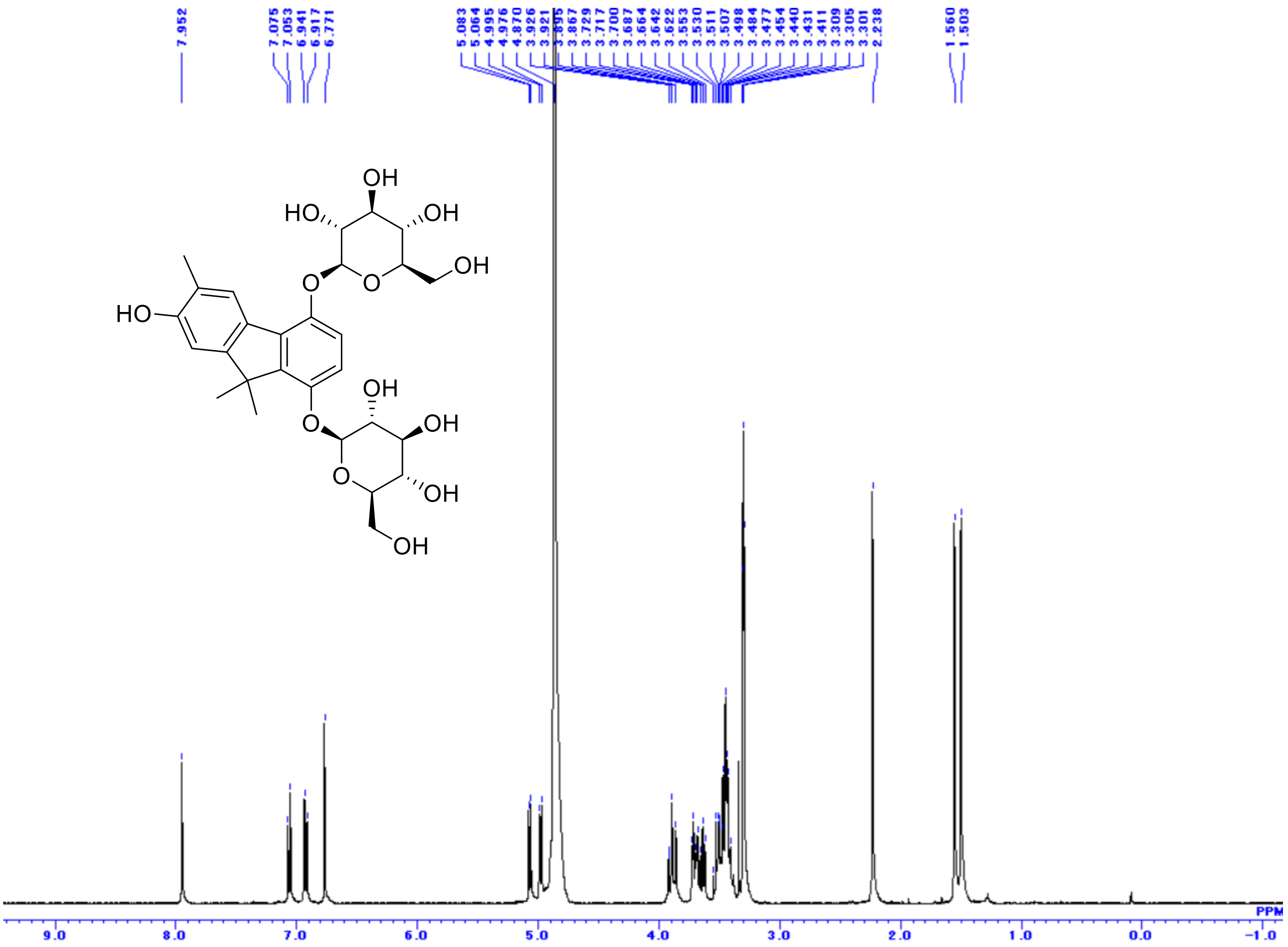

Figure S5 ^{1}H NMR spectrum of compound **1** (in methanol-d_4).

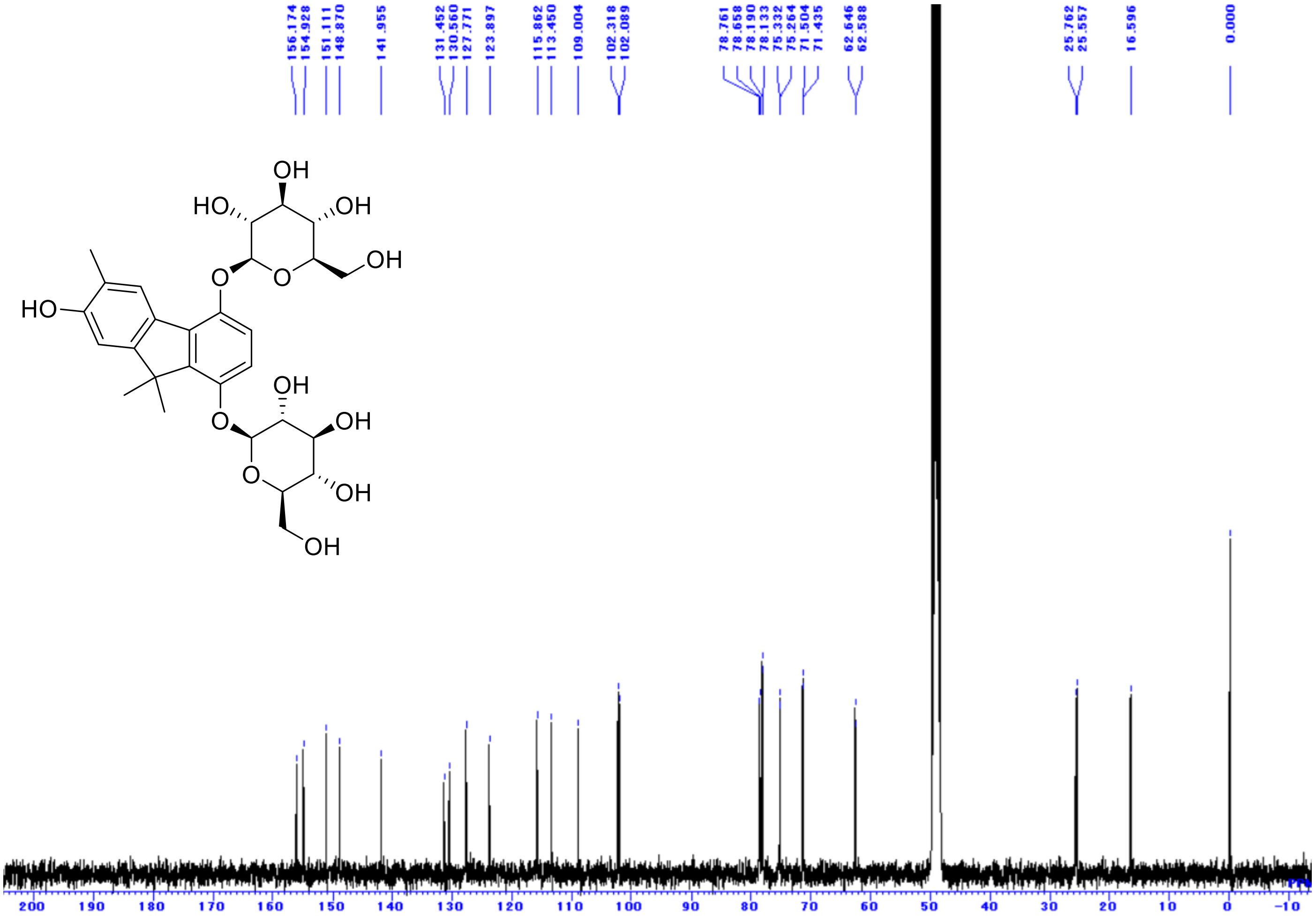

Figure S6 ^{13}C NMR spectrum of compound **1** (in methanol-d_4).

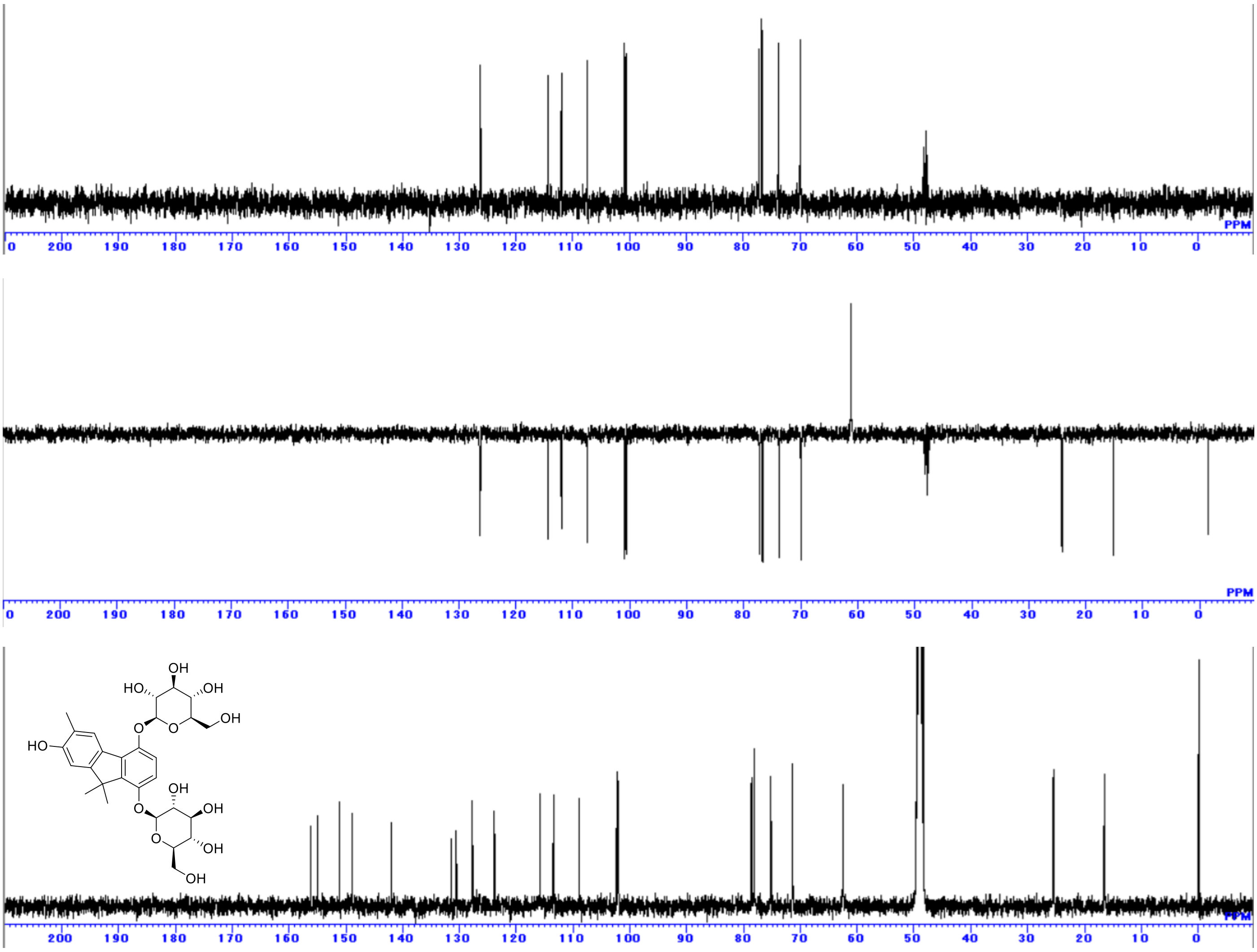

Figure S7 DEPT spectrum of compound **1** (in methanol-d_4).

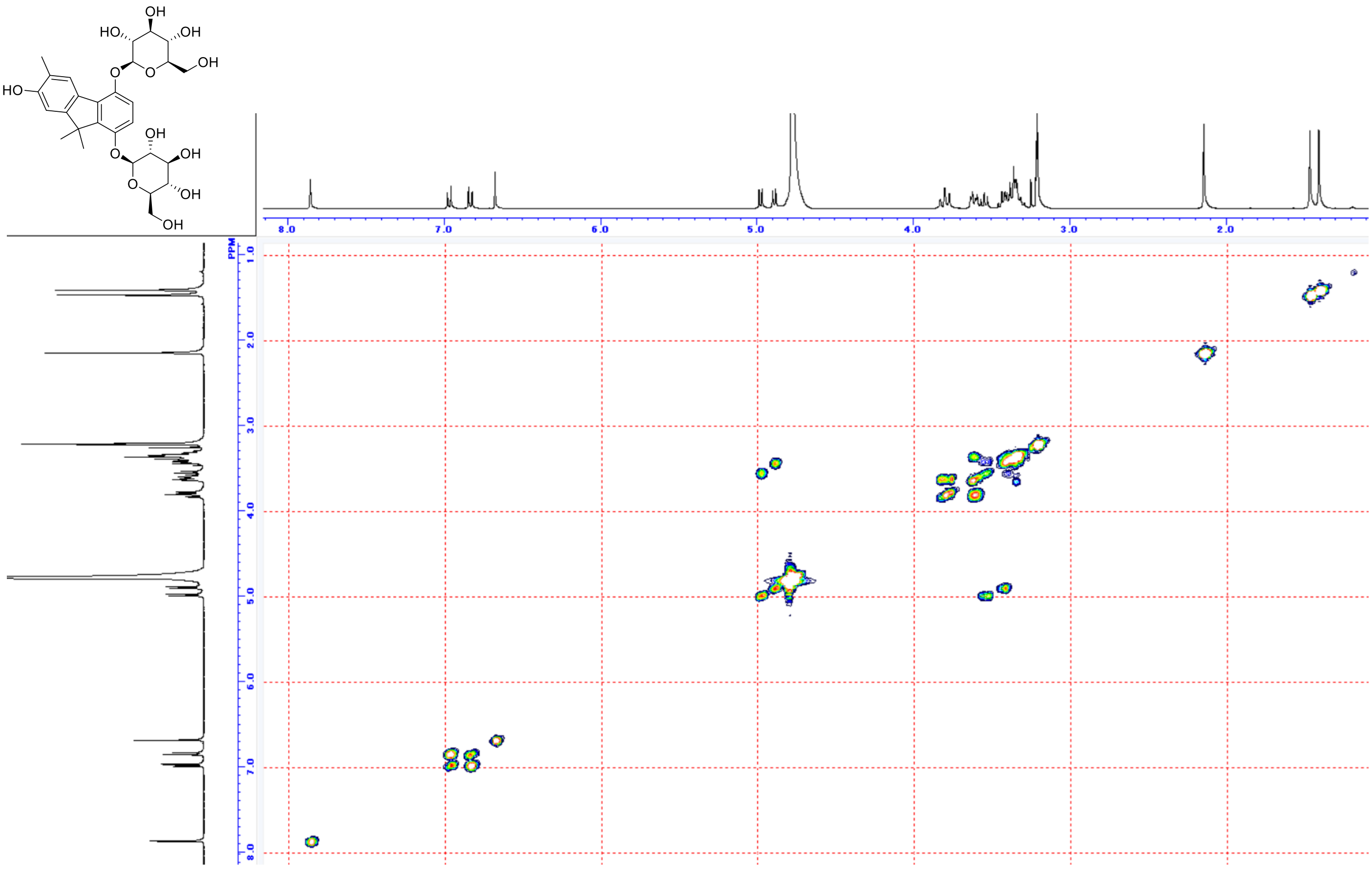

Figure S8 ^{1}H-^{1}H COSY spectrum of compound **1** (in methanol-d_4).

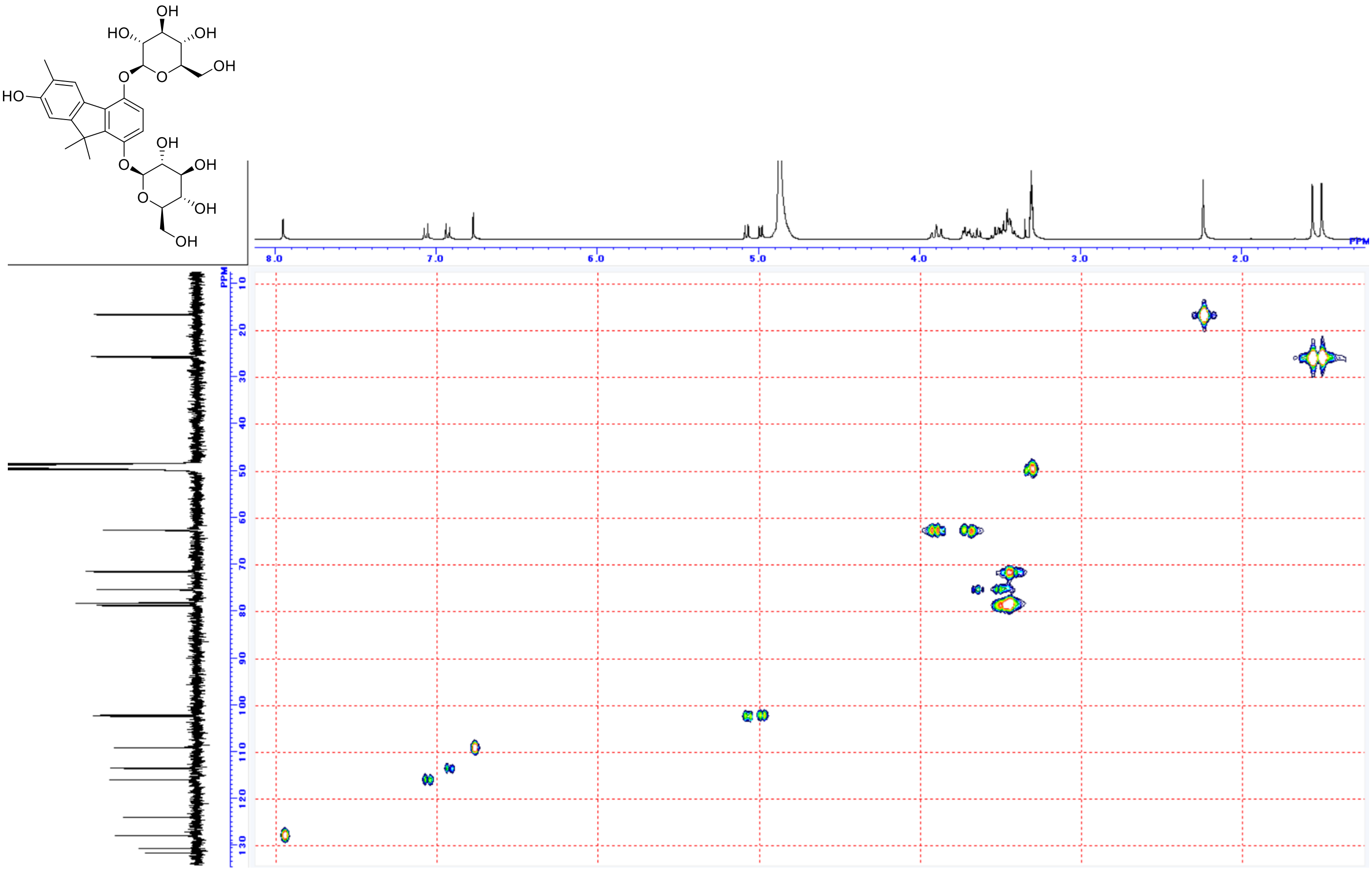

Figure S9 HMQC spectrum of compound **1** (in methanol-d_4).

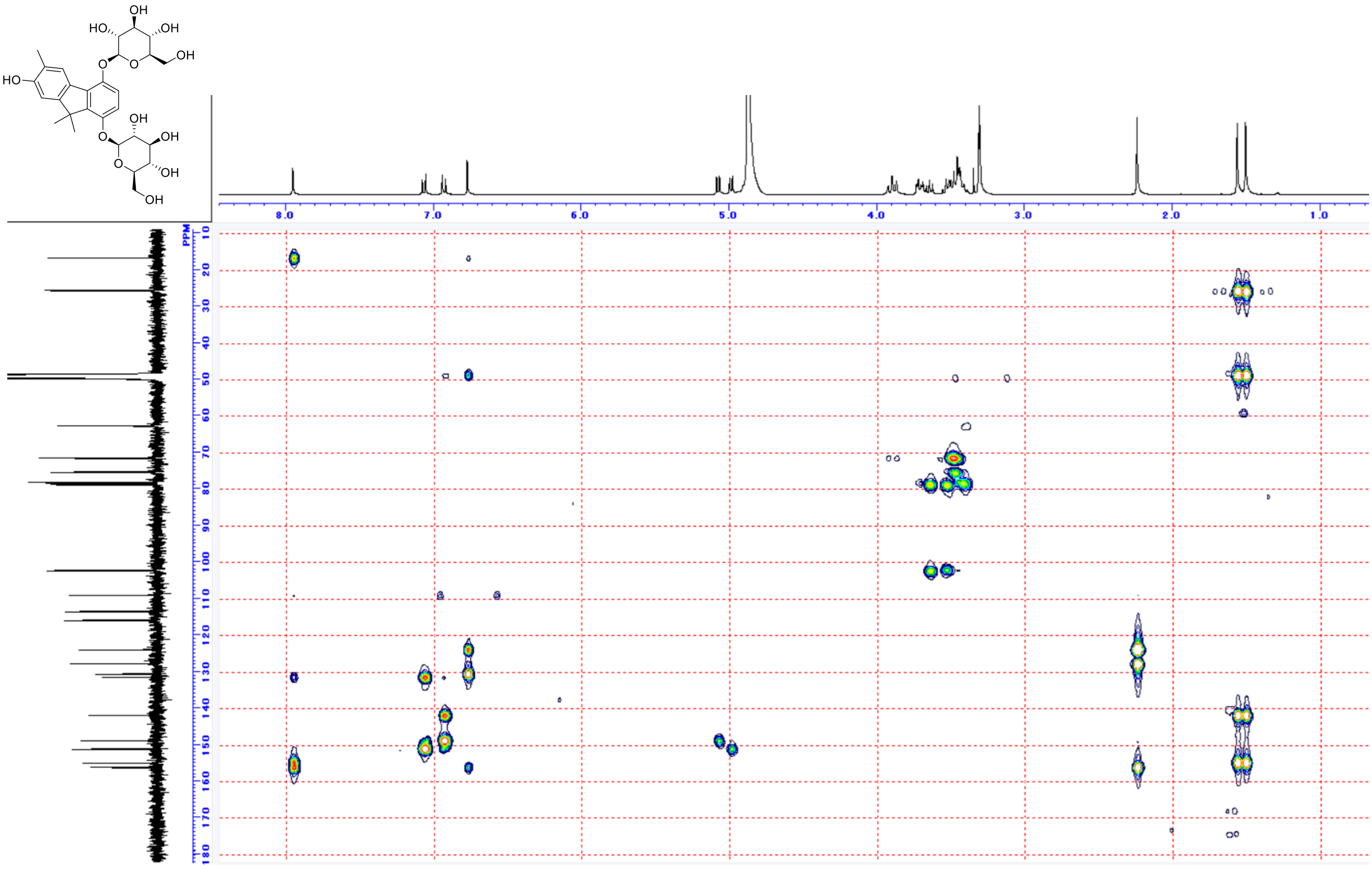

Figure S10 HMBC spectrum of compound **1** (in methanol-d_4).

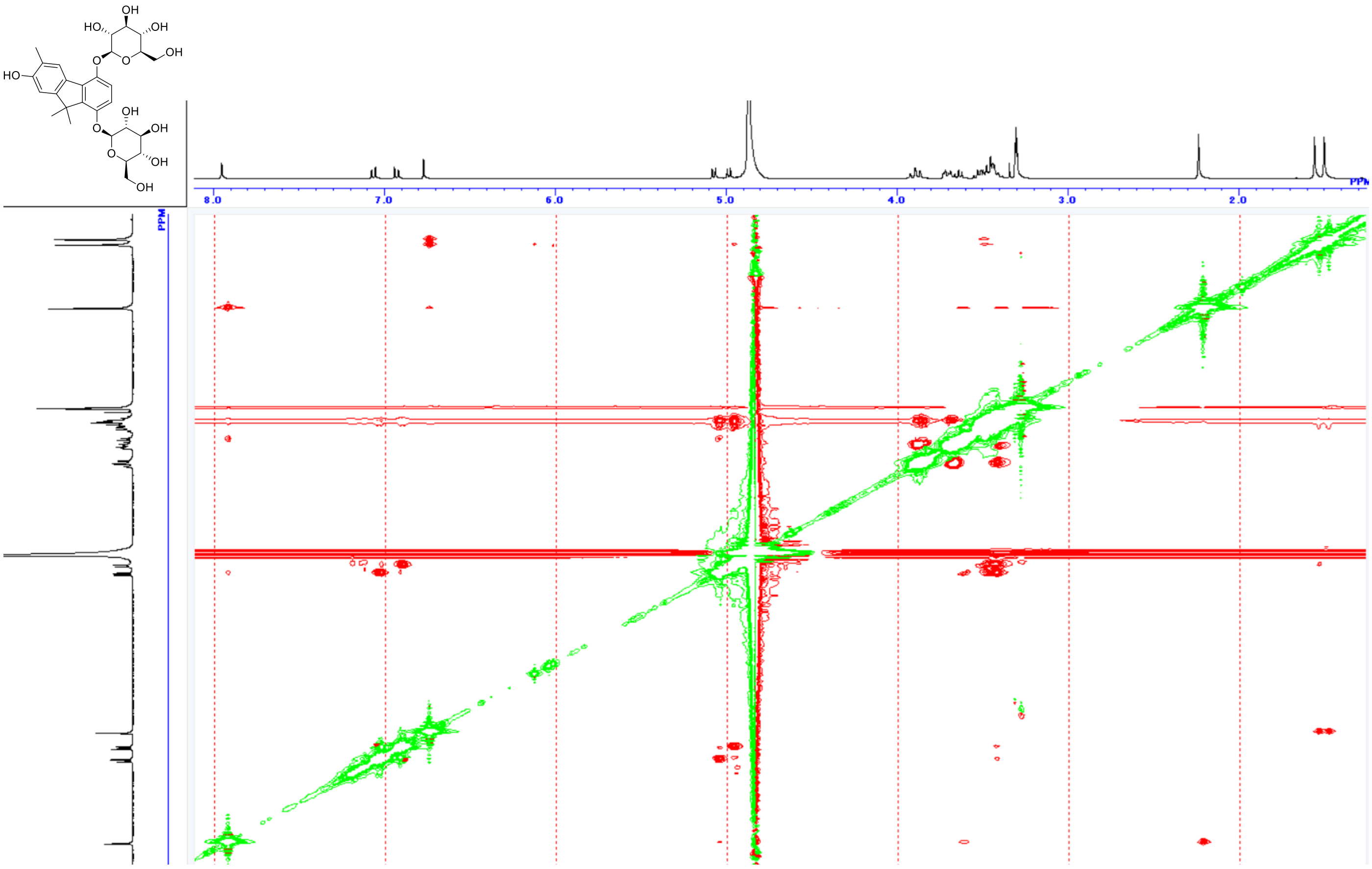

Figure S11 NOESY spectra of compound **1** (in methanol-d_4).

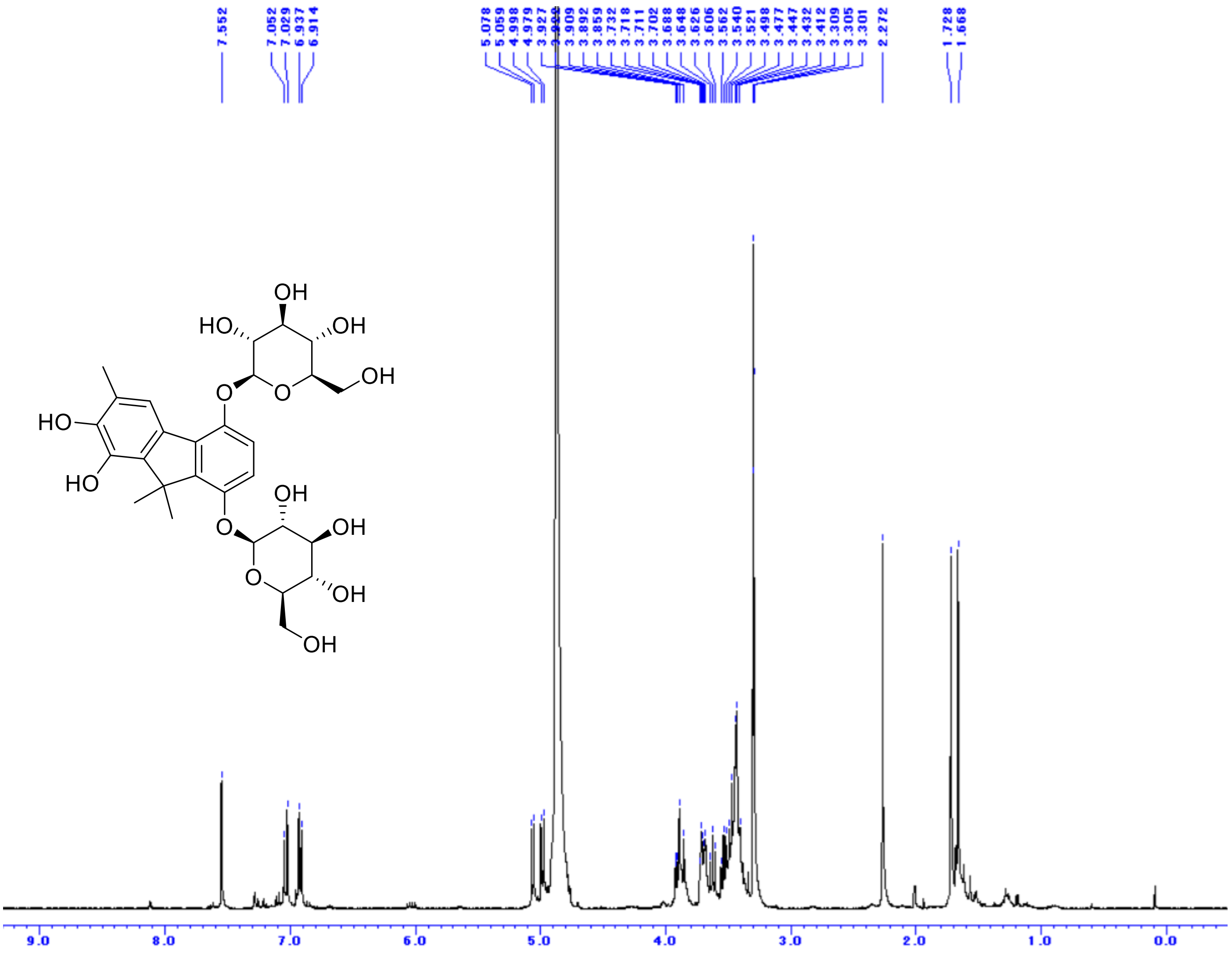

Figure S12 ^{1}H NMR spectrum of compound **2** (in methanol-d_4).

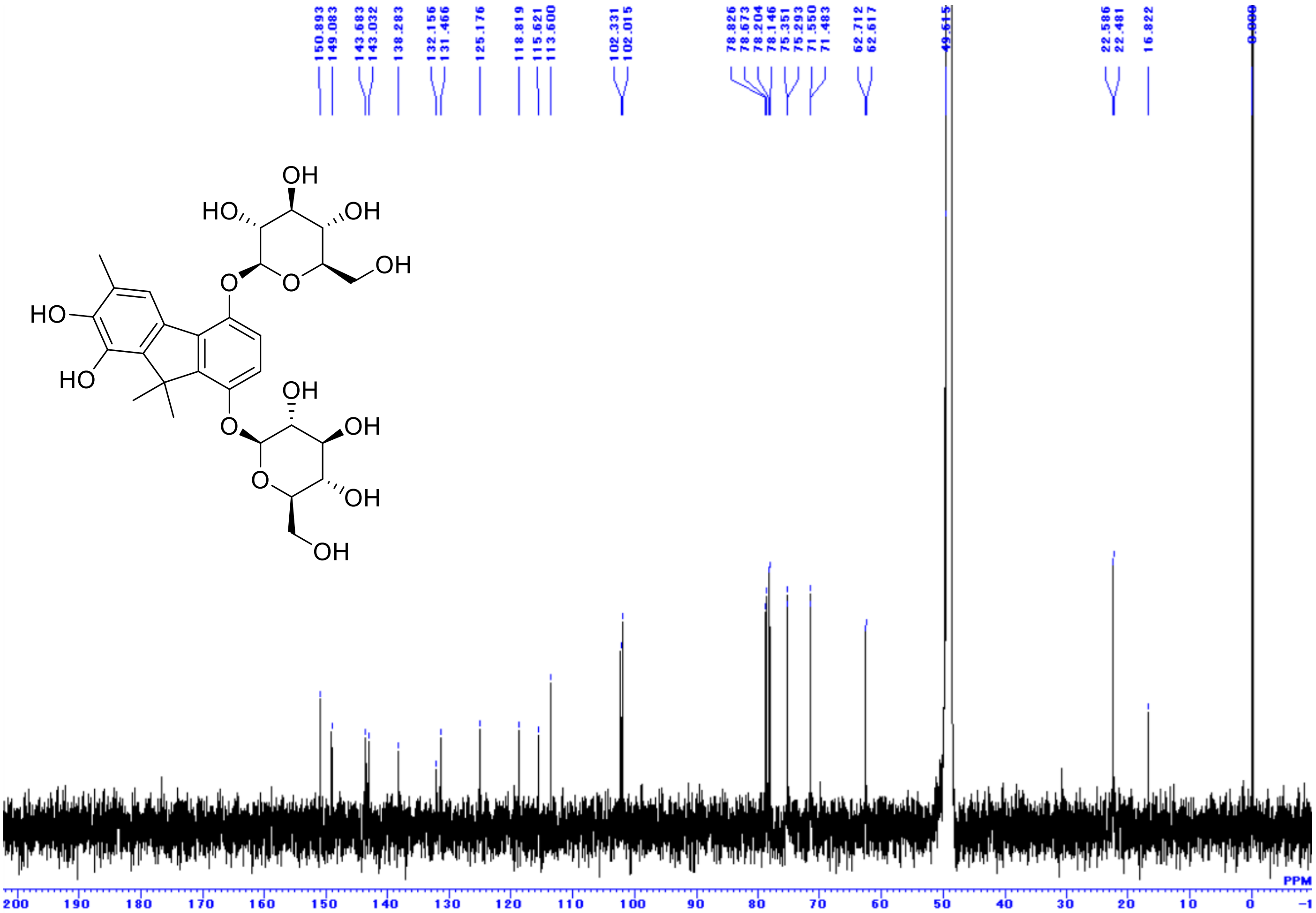

Figure S13 ^{13}C NMR spectrum of compound **2** (in methanol-d_4).

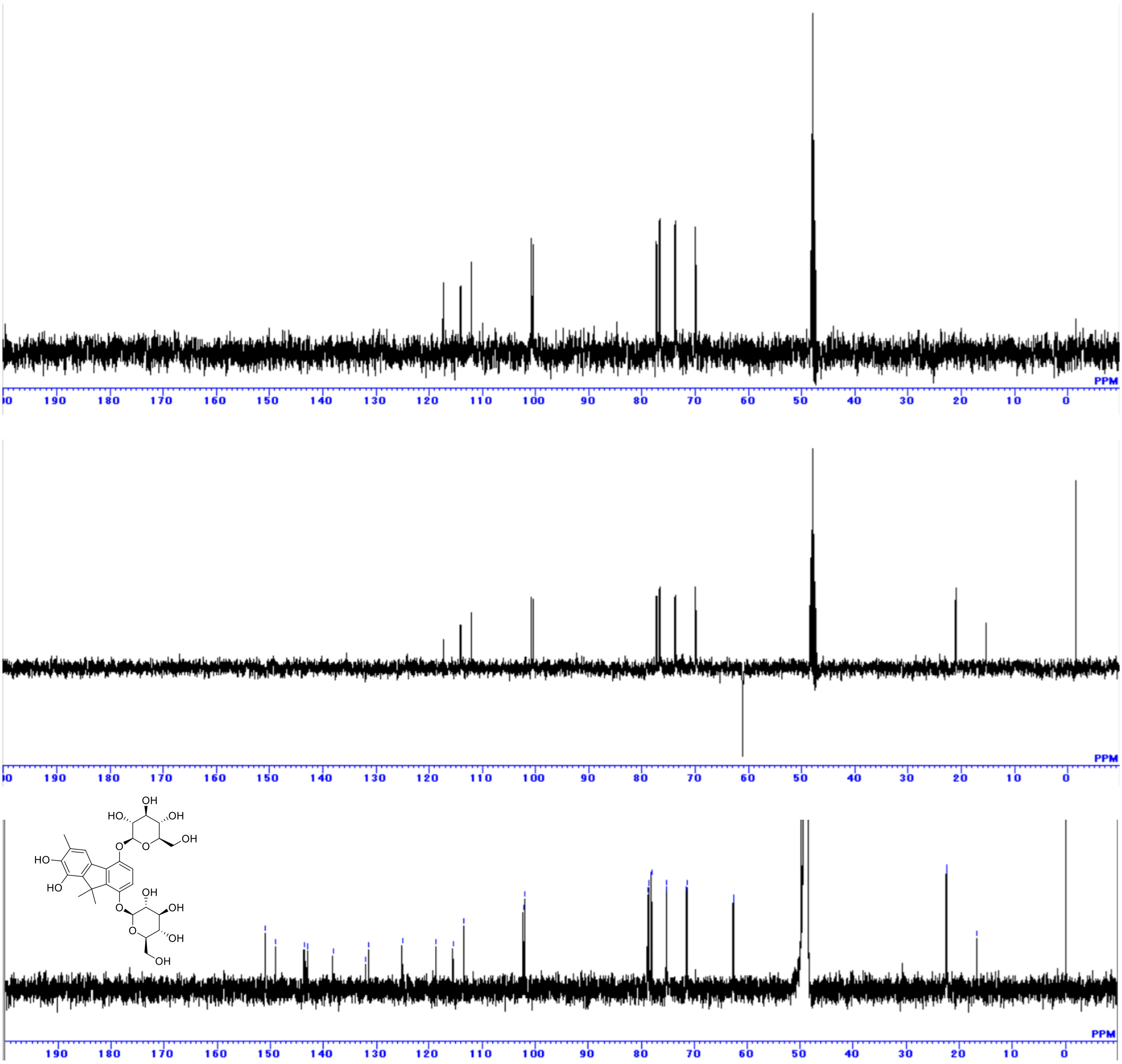

Figure S14 DEPT spectrum of compound **2** (in methanol-d_4).

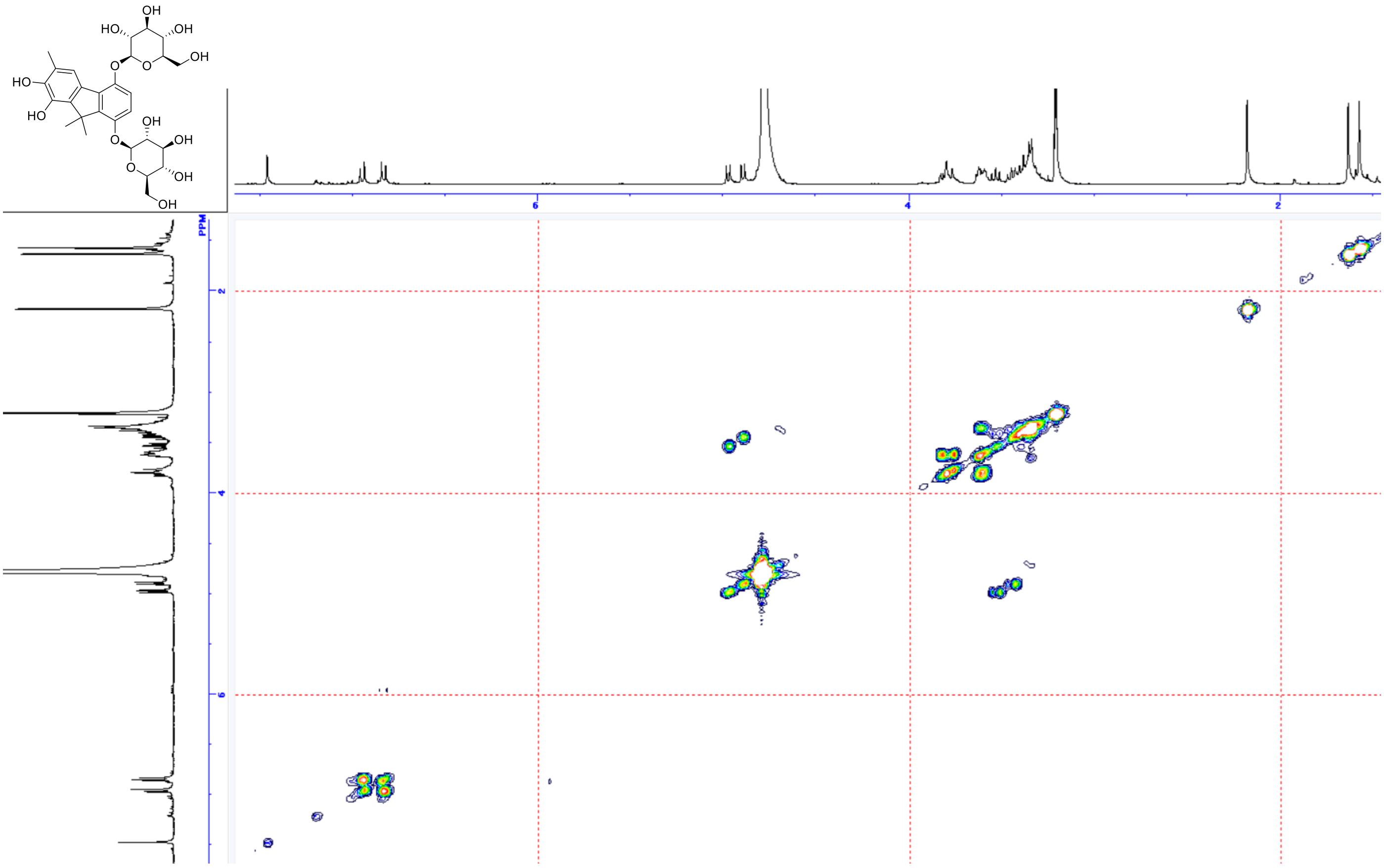

Figure S15 ^{1}H-^{1}H COSY spectrum of compound **2** (in methanol-d_4).

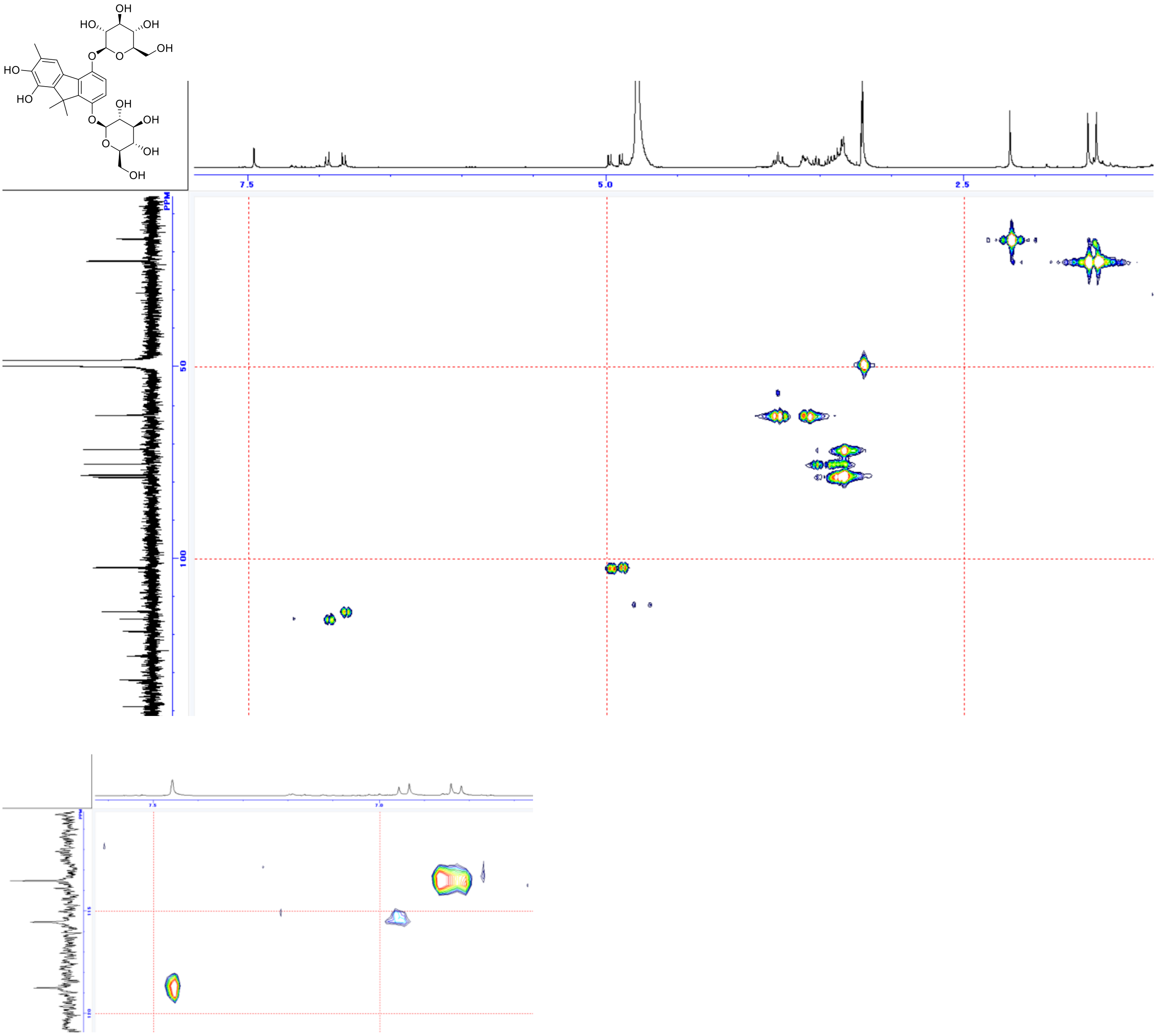

Figure S16 HMQC spectrum of compound **2** (in methanol-d_4).

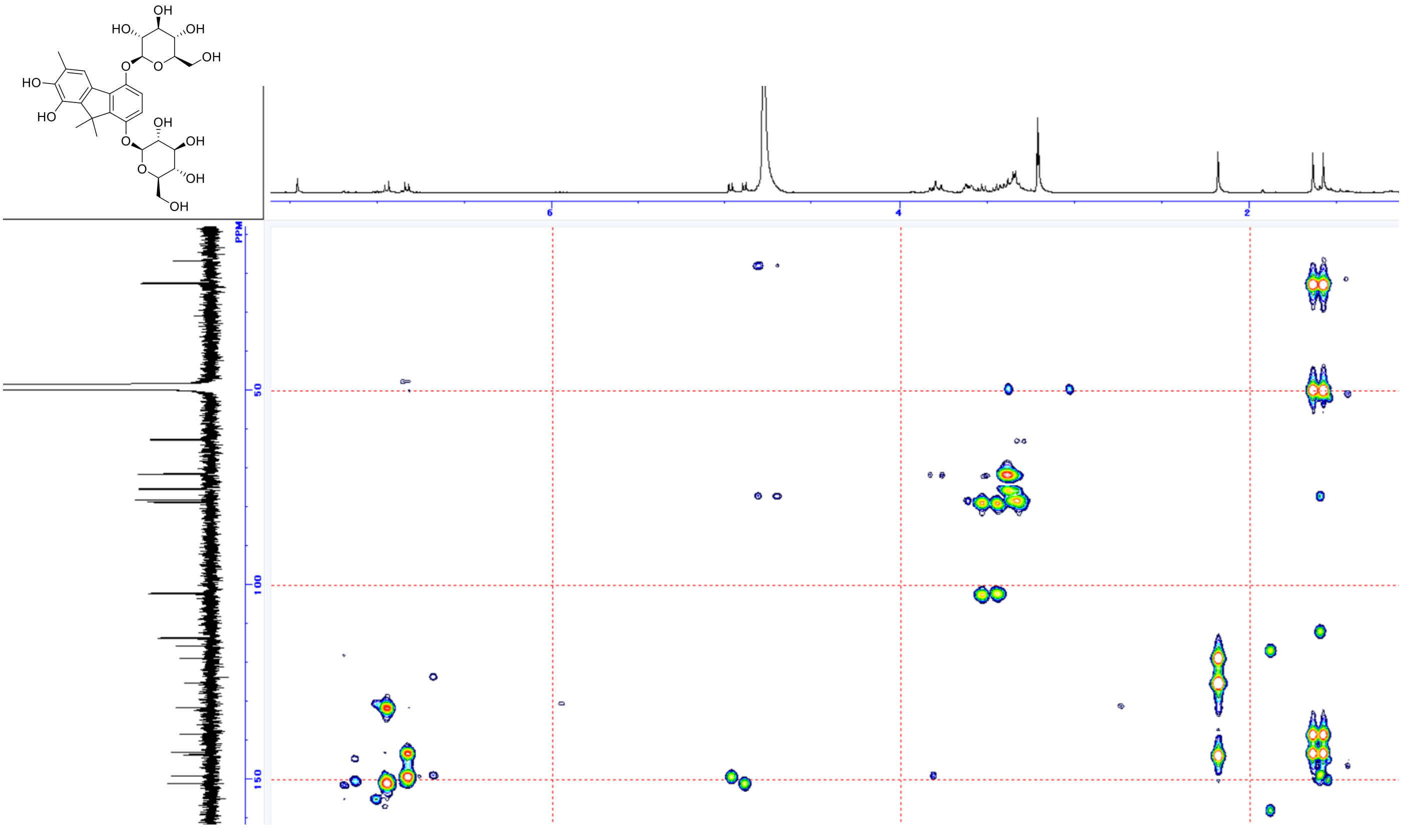

Figure S17 HMBC spectrum of compound **2** (in methanol-d_4).

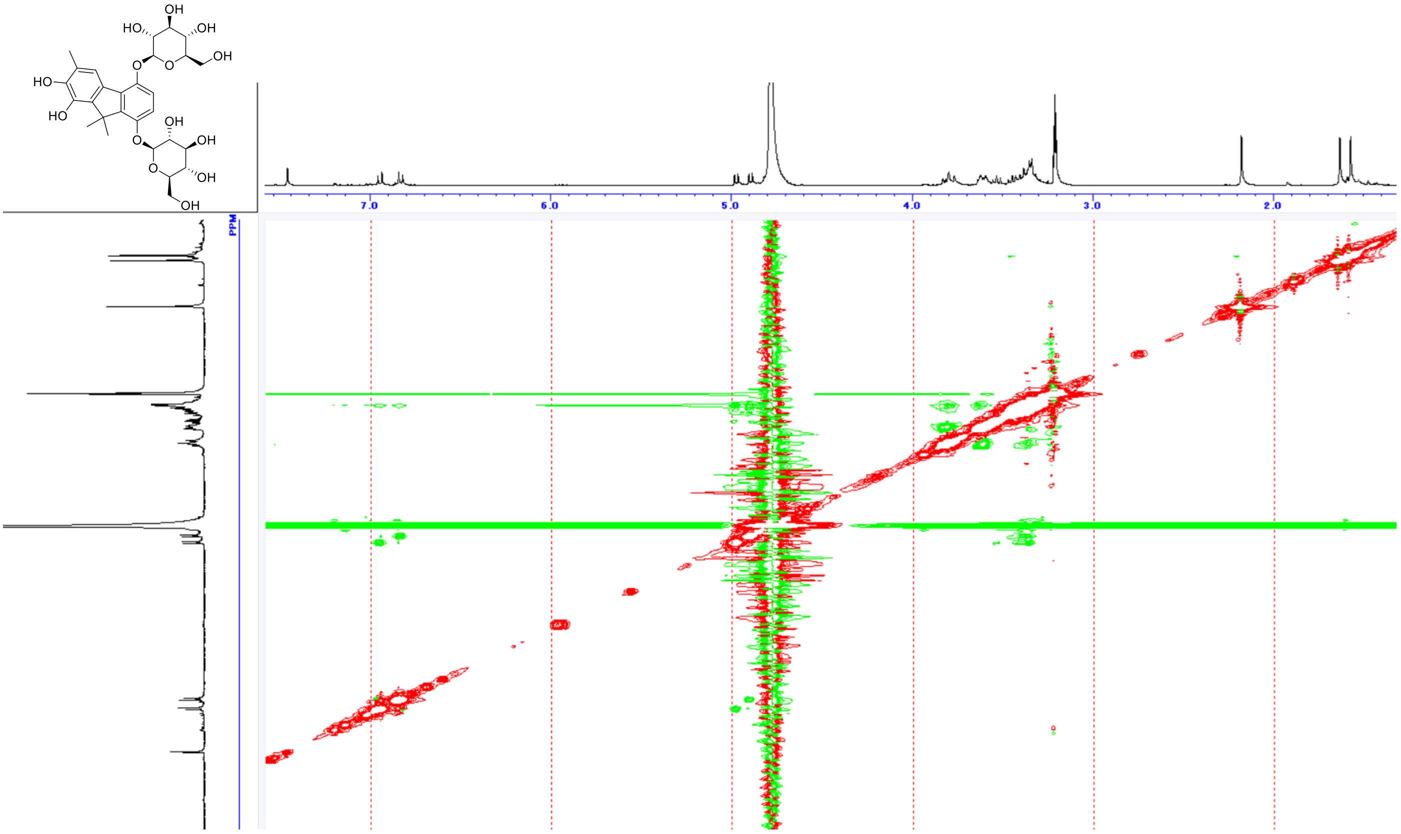

Figure S18 NOESY spectra of compound **2** (in methanol-d_4).

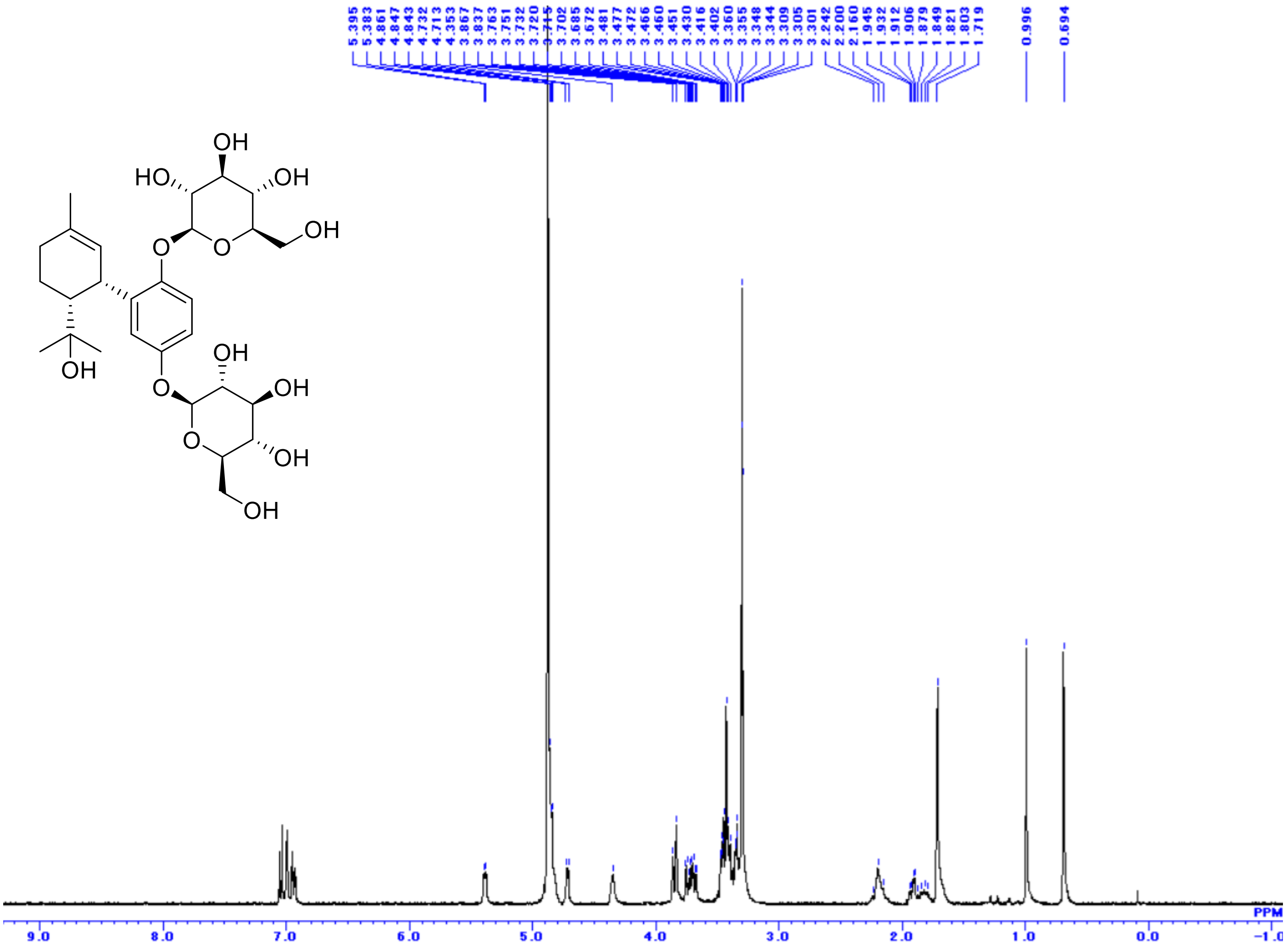

Figure S19 ^{1}H NMR spectrum of compound **3** (in methanol-d_4).

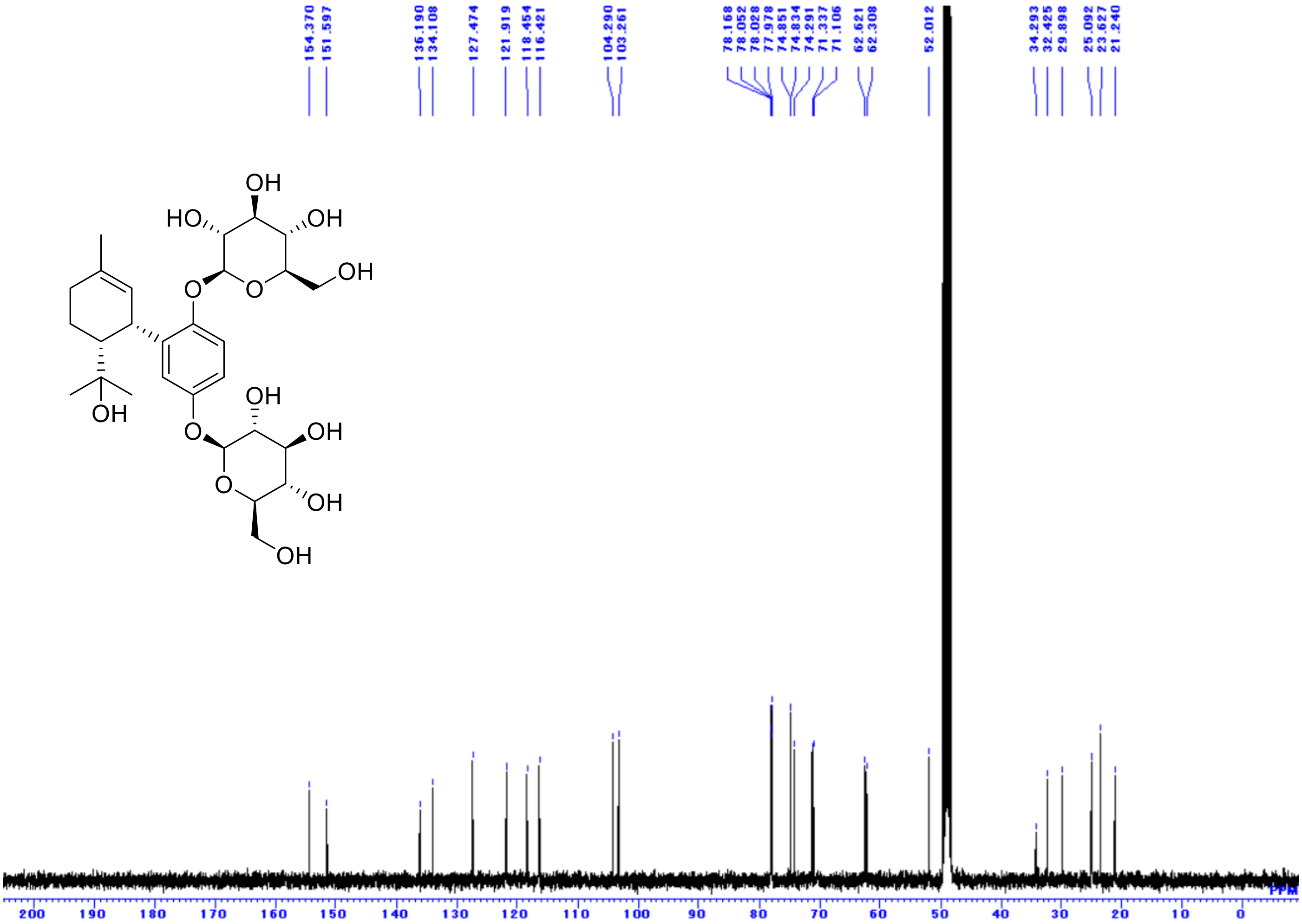

Figure S20 ^{13}C NMR spectrum of compound **3** (in methanol-*d*4).

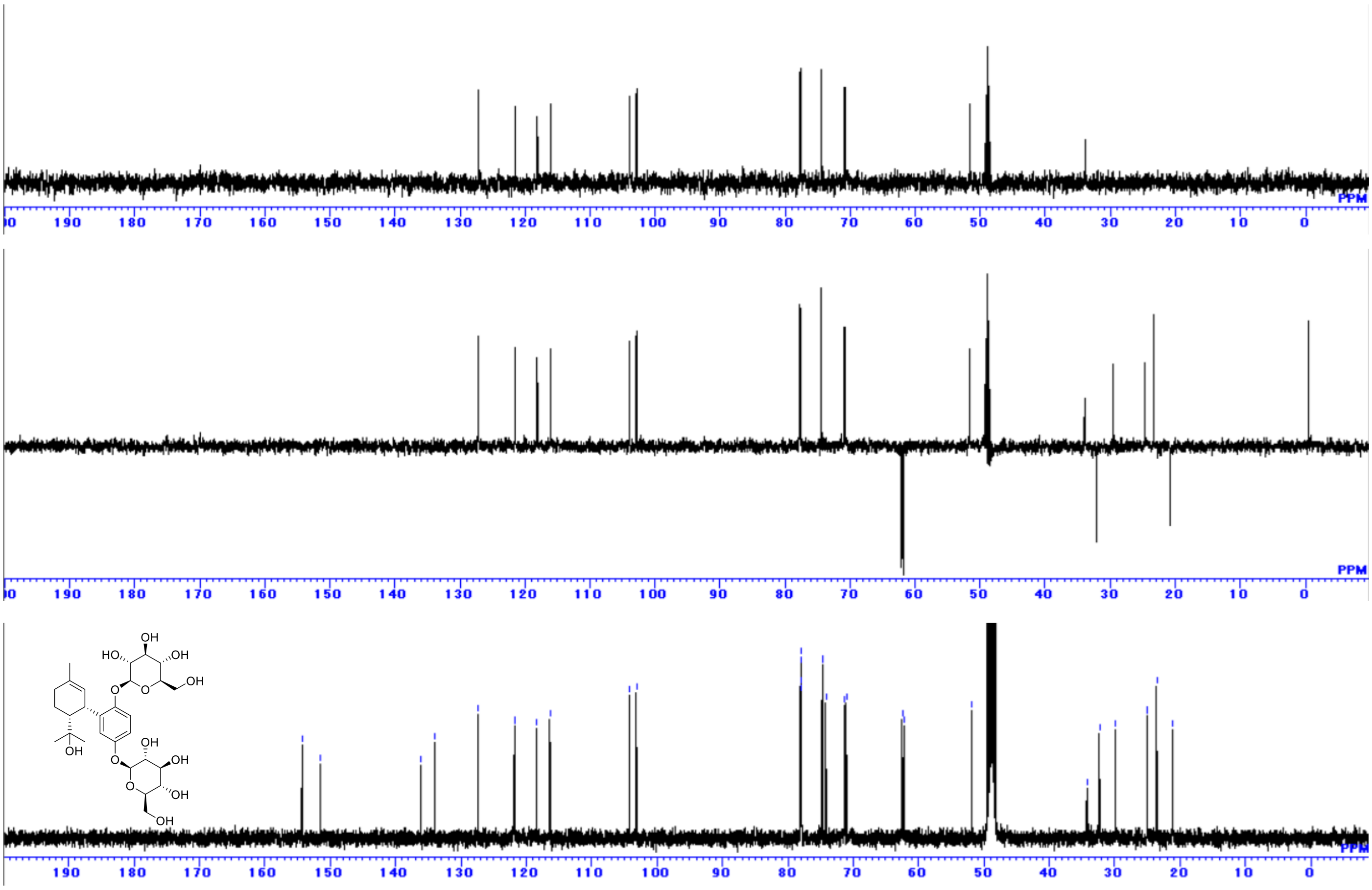

Figure S21 DEPT spectrum of compound **3** (in methanol-d_4).

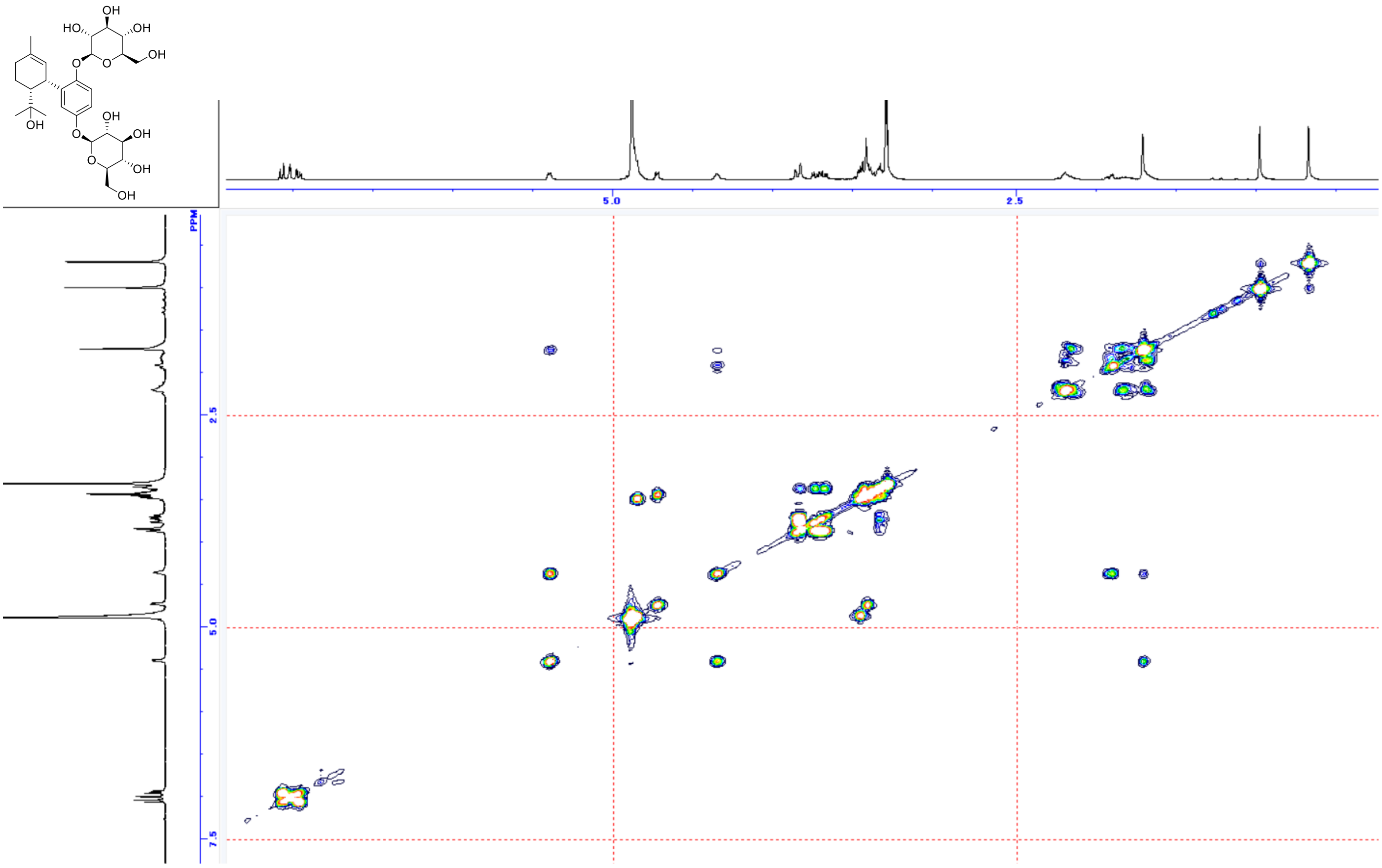

Figure S22 ^{1}H-^{1}H COSY spectrum of compound **3** (in methanol-d_4).

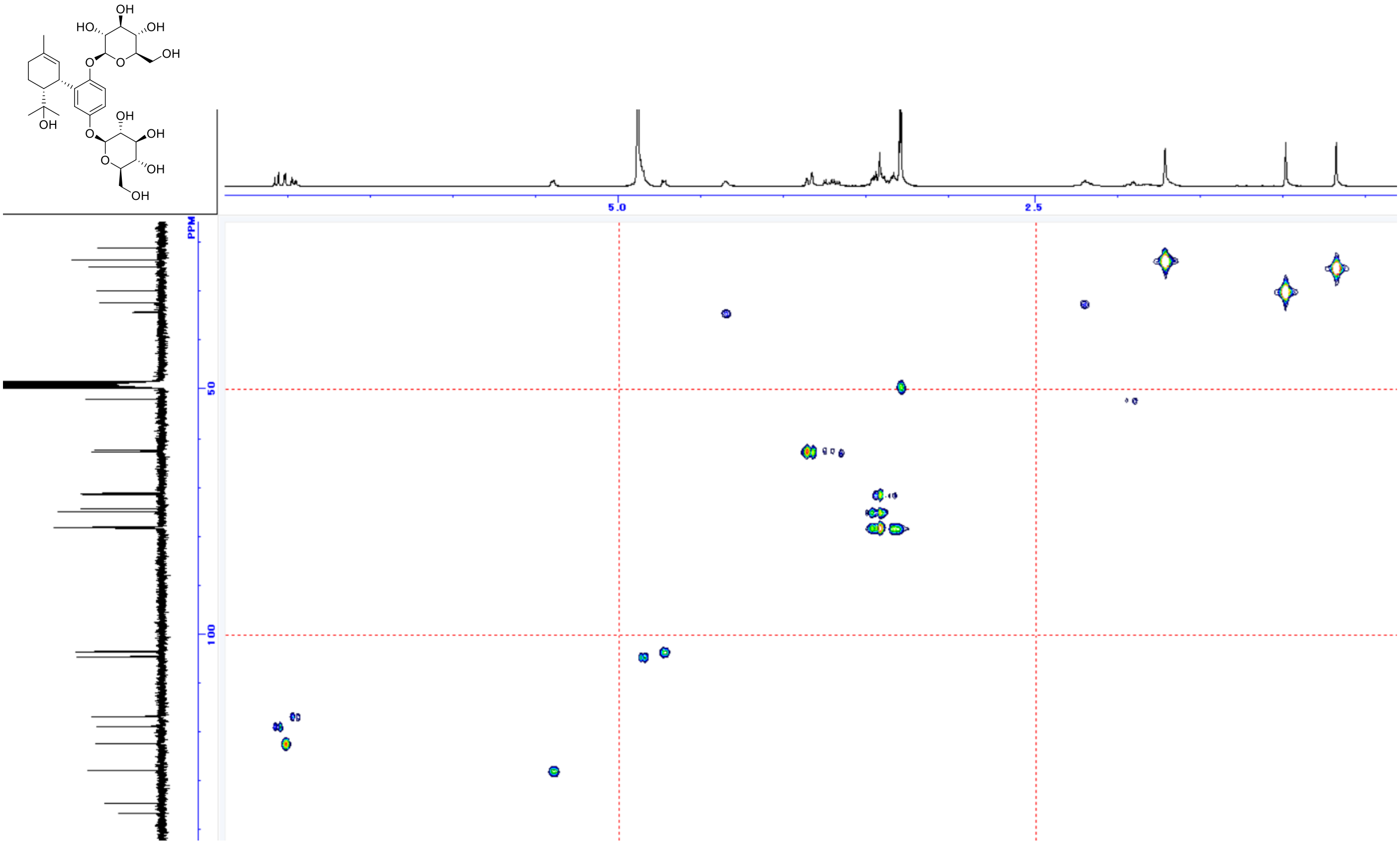

Figure S23 HMQC spectrum of compound **3** (in methanol-d_4).

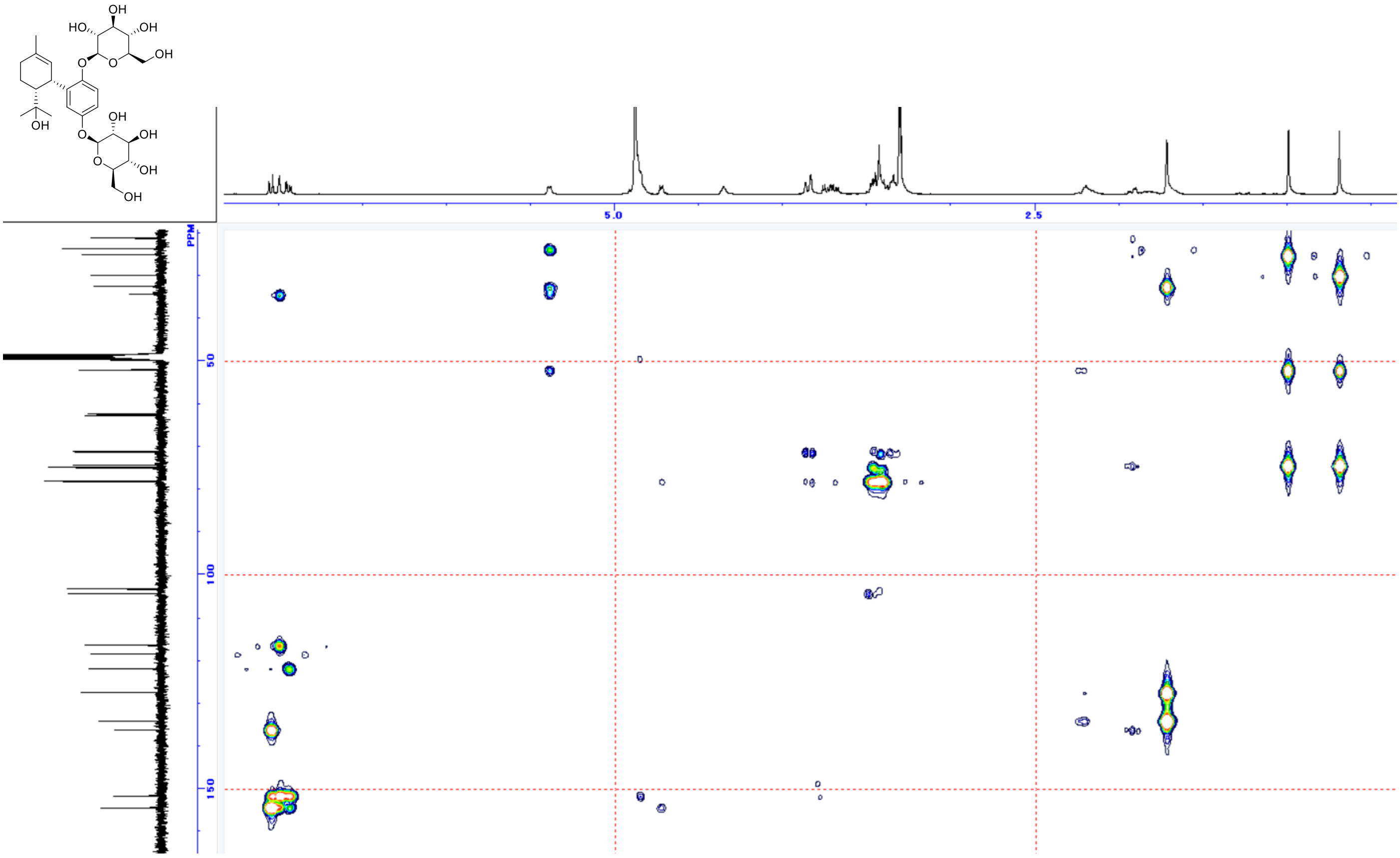

Figure S24 HMBC spectrum of compound **3** (in methanol-*d*4).

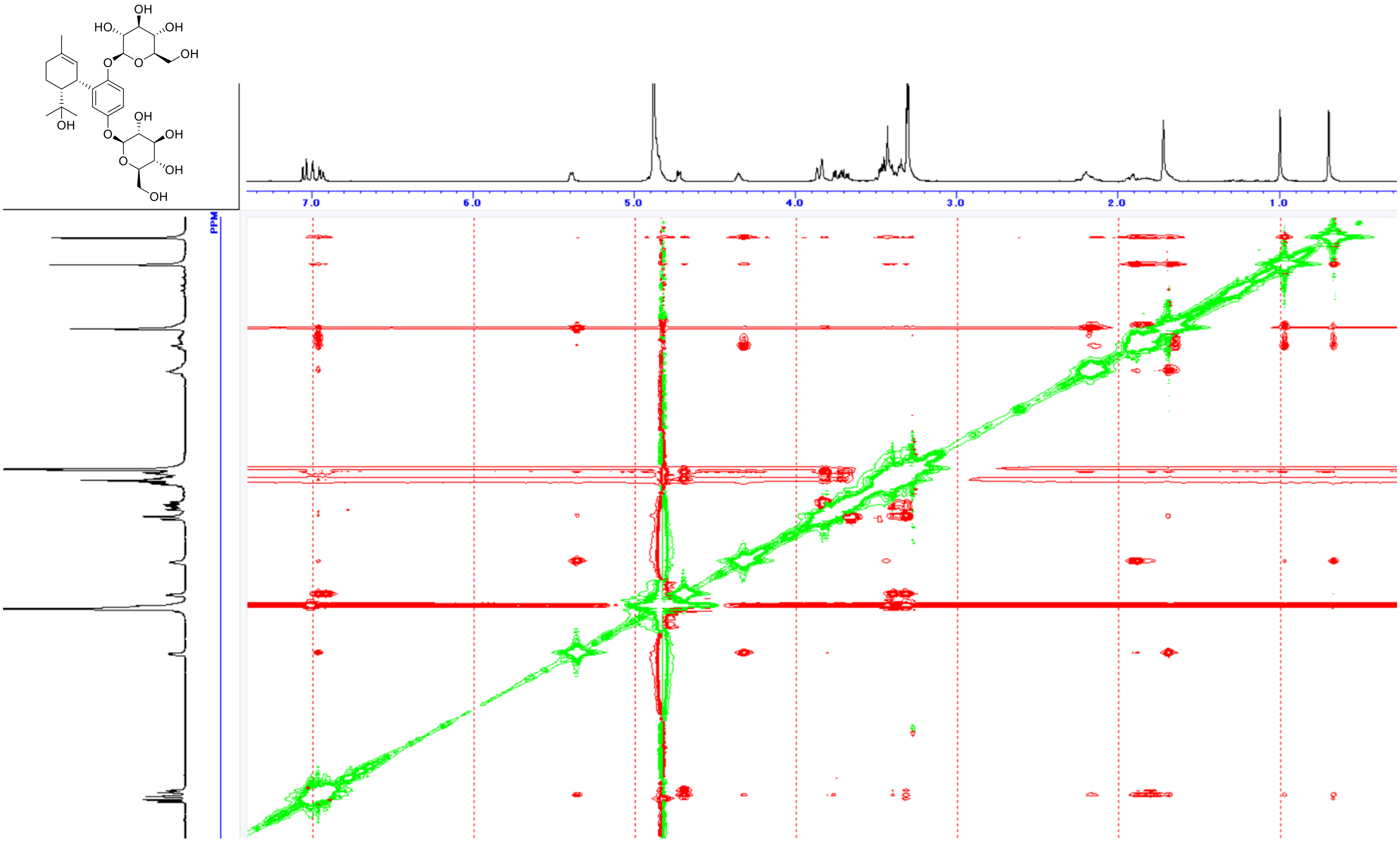

Figure S25 NOESY spectra of compound **3** (in methanol-*d*4).

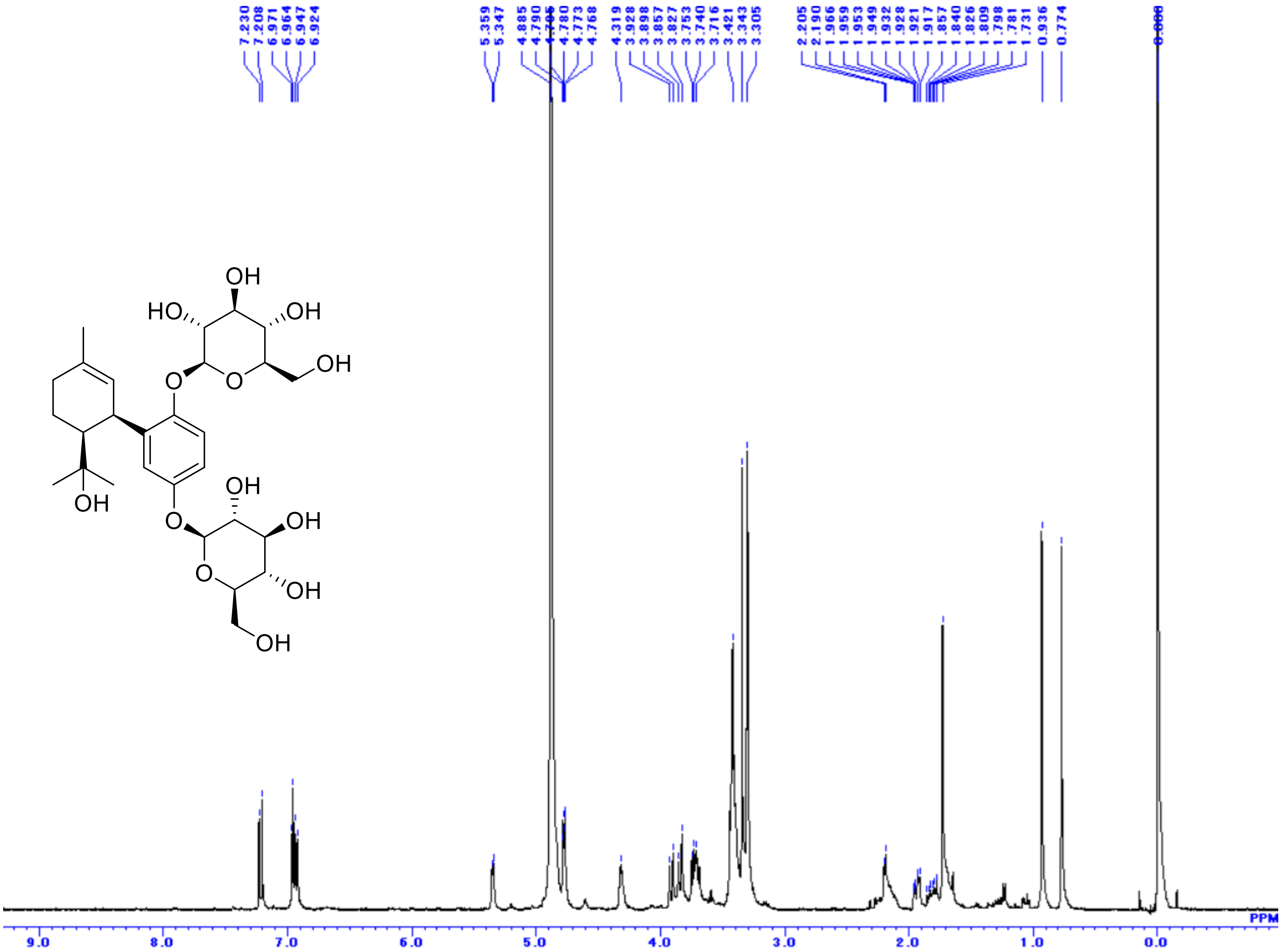

Figure S26 ^{1}H NMR spectrum of compound **4** (in methanol-d_4).

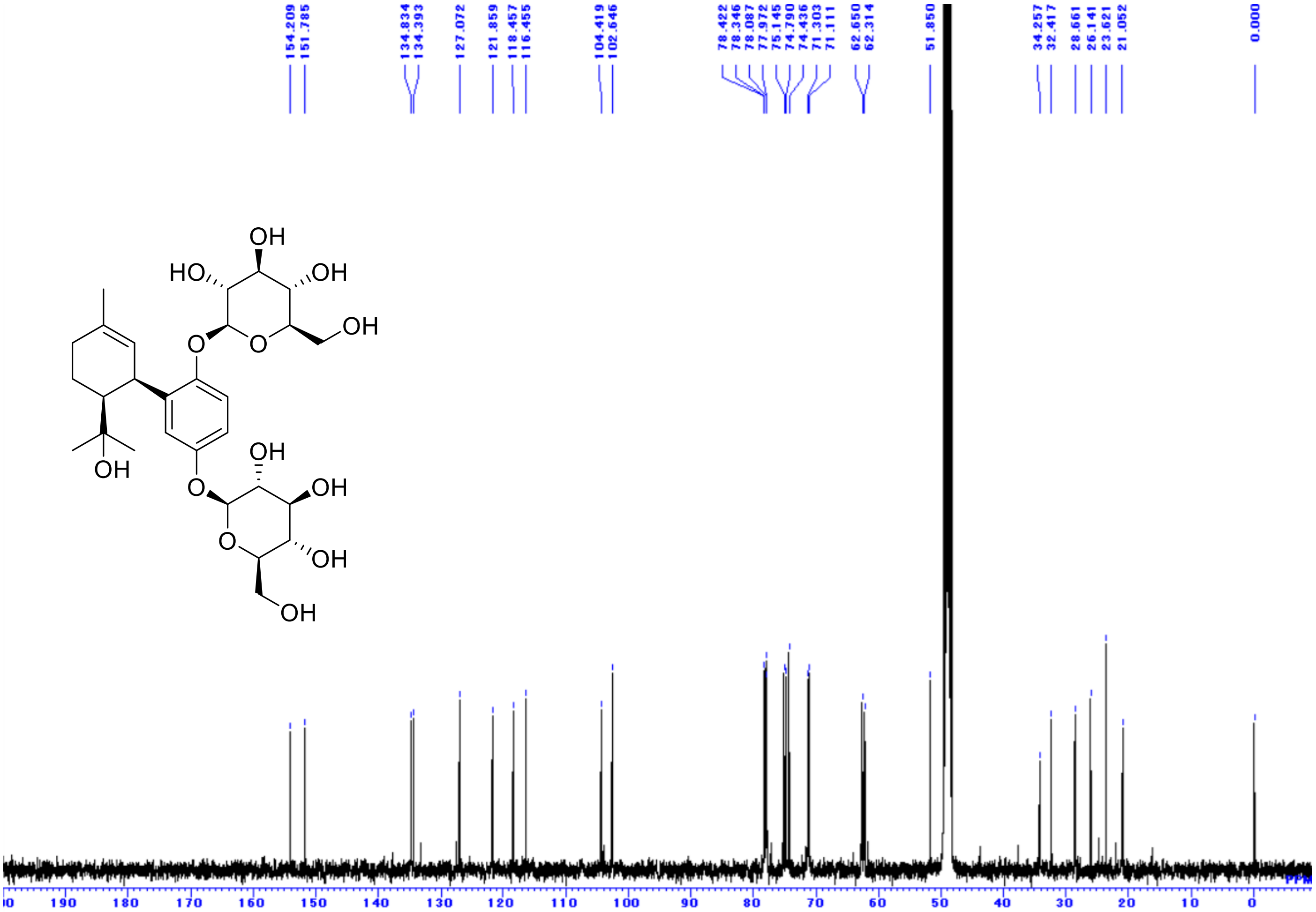

Figure S27 ^{13}C NMR spectrum of compound **4** (in methanol-d_4).

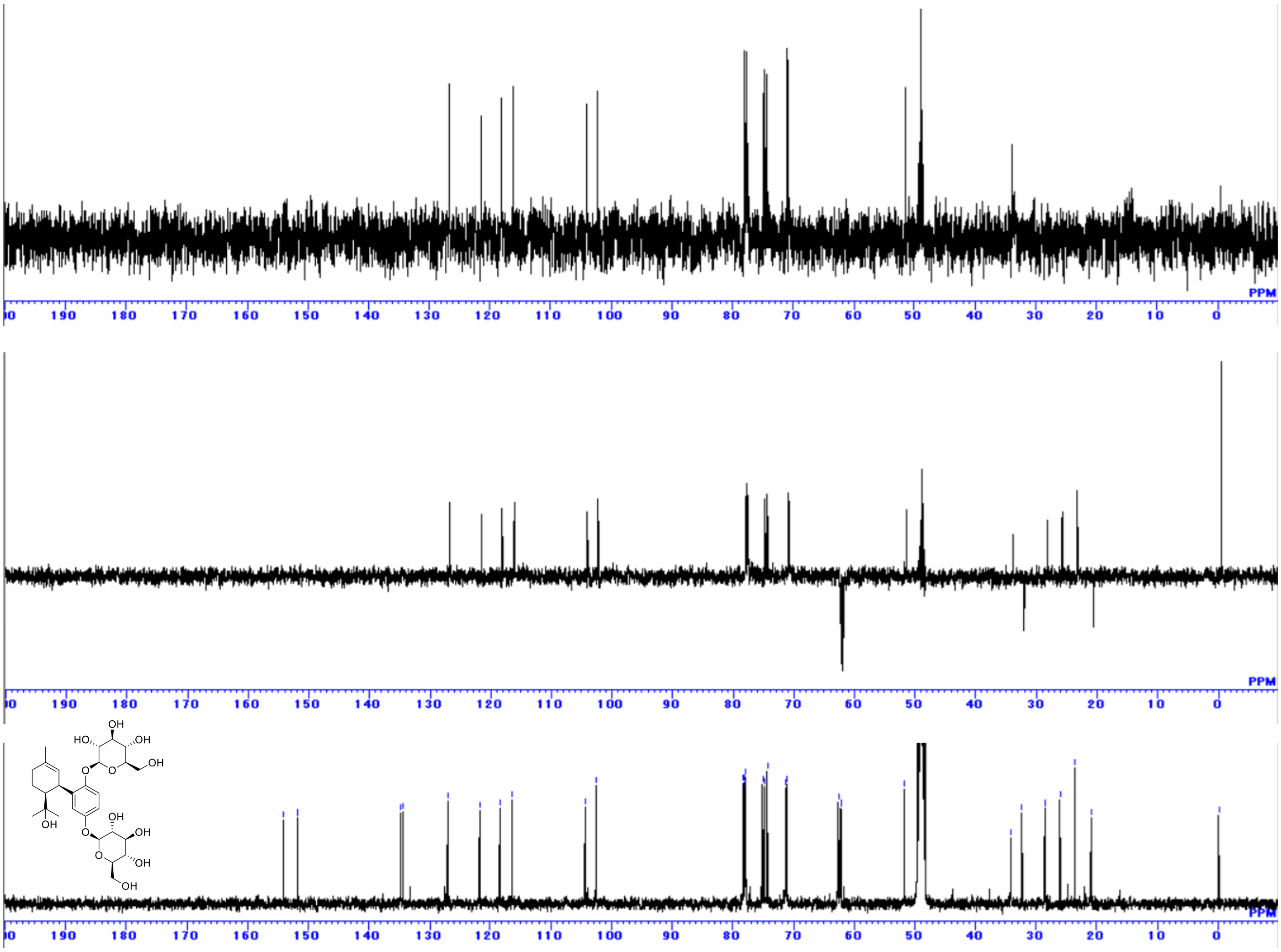

Figure S28 DEPT spectrum of compound **4** (in methanol-d_4).

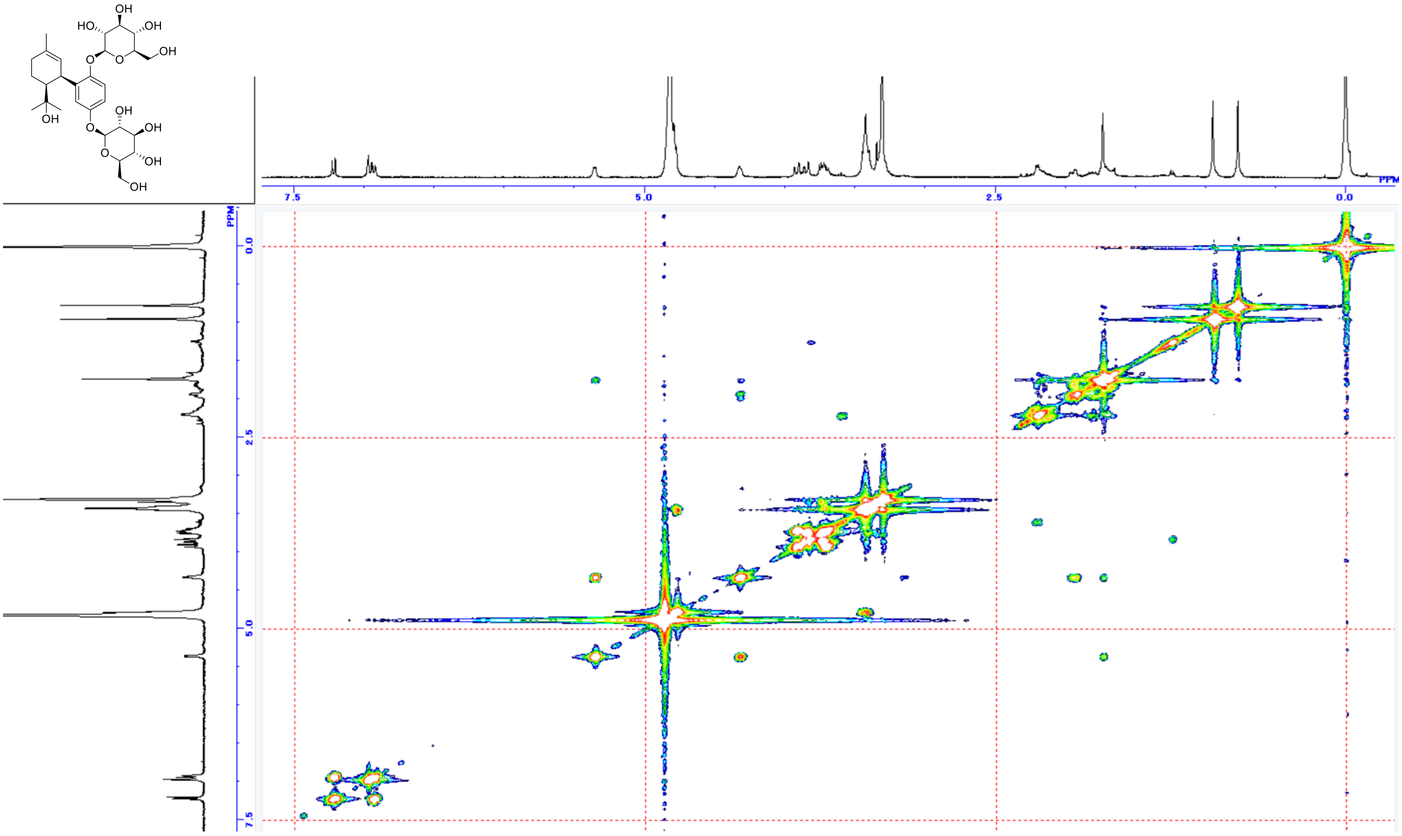

Figure S29 ^{1}H-^{1}H COSY spectrum of compound **4** (in methanol-*d*$_4$).

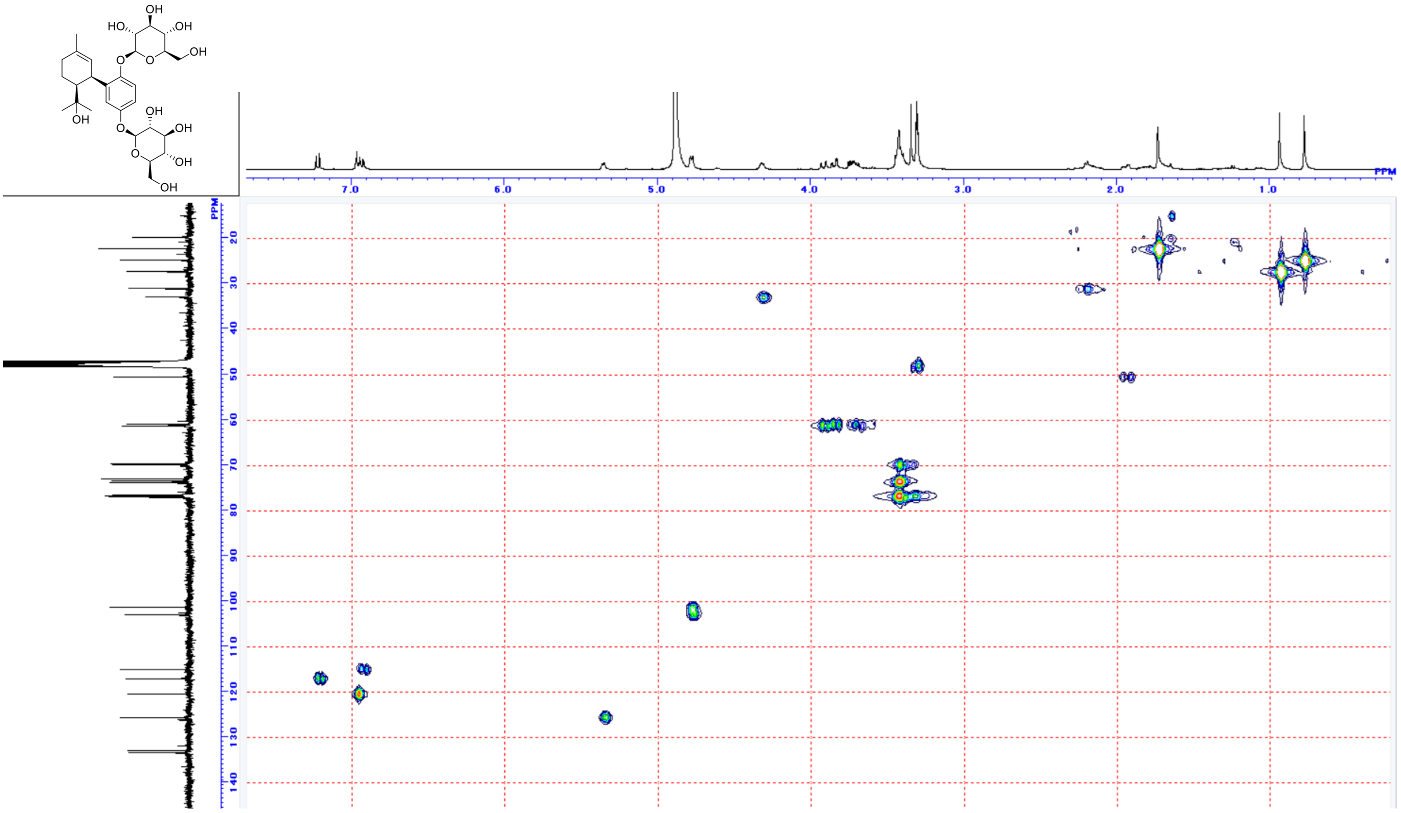

Figure S30 HMQC spectrum of compound **4** (in methanol-*d*$_4$).

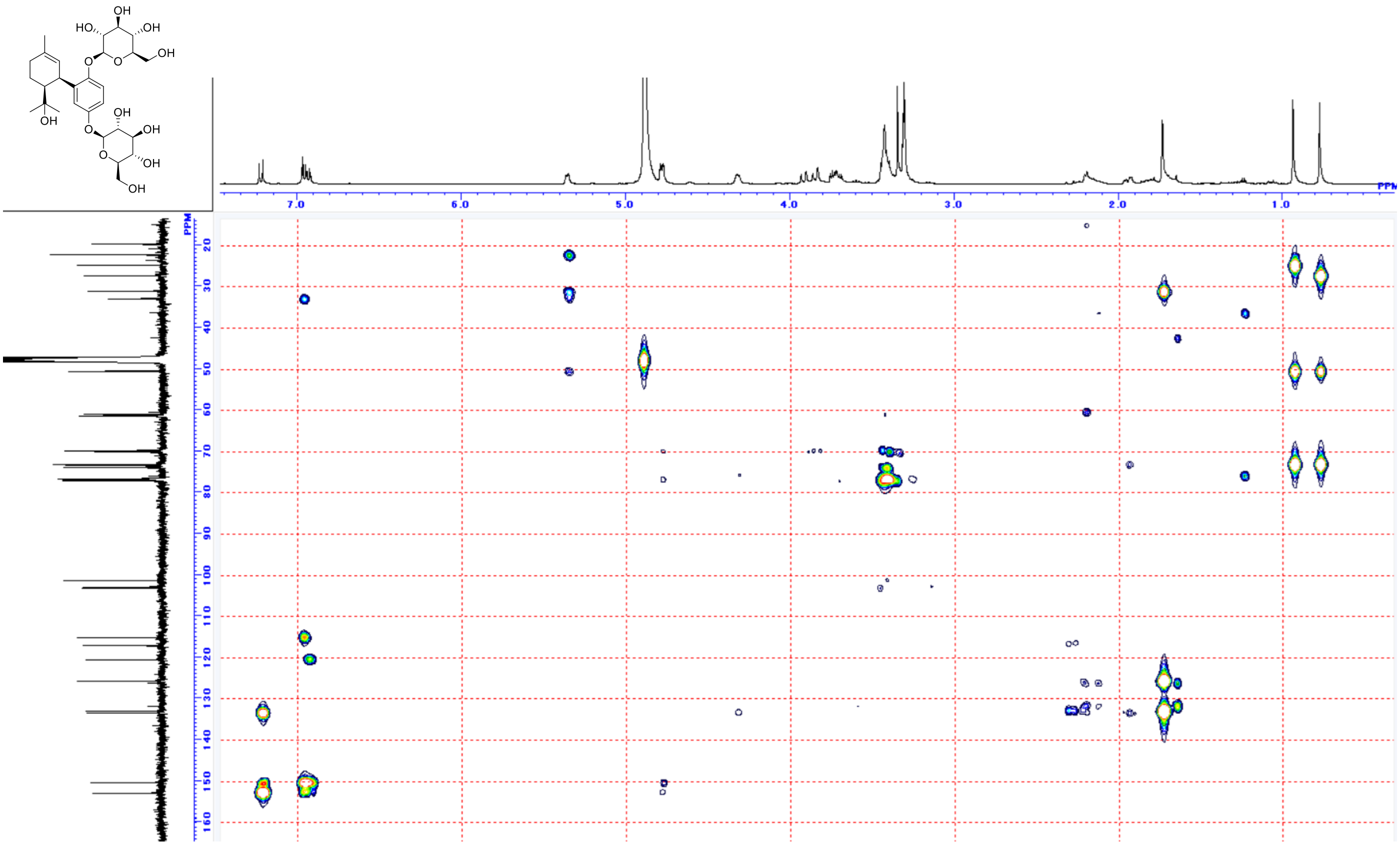

Figure S31 HMBC spectrum of compound **4** (in methanol-d_4).

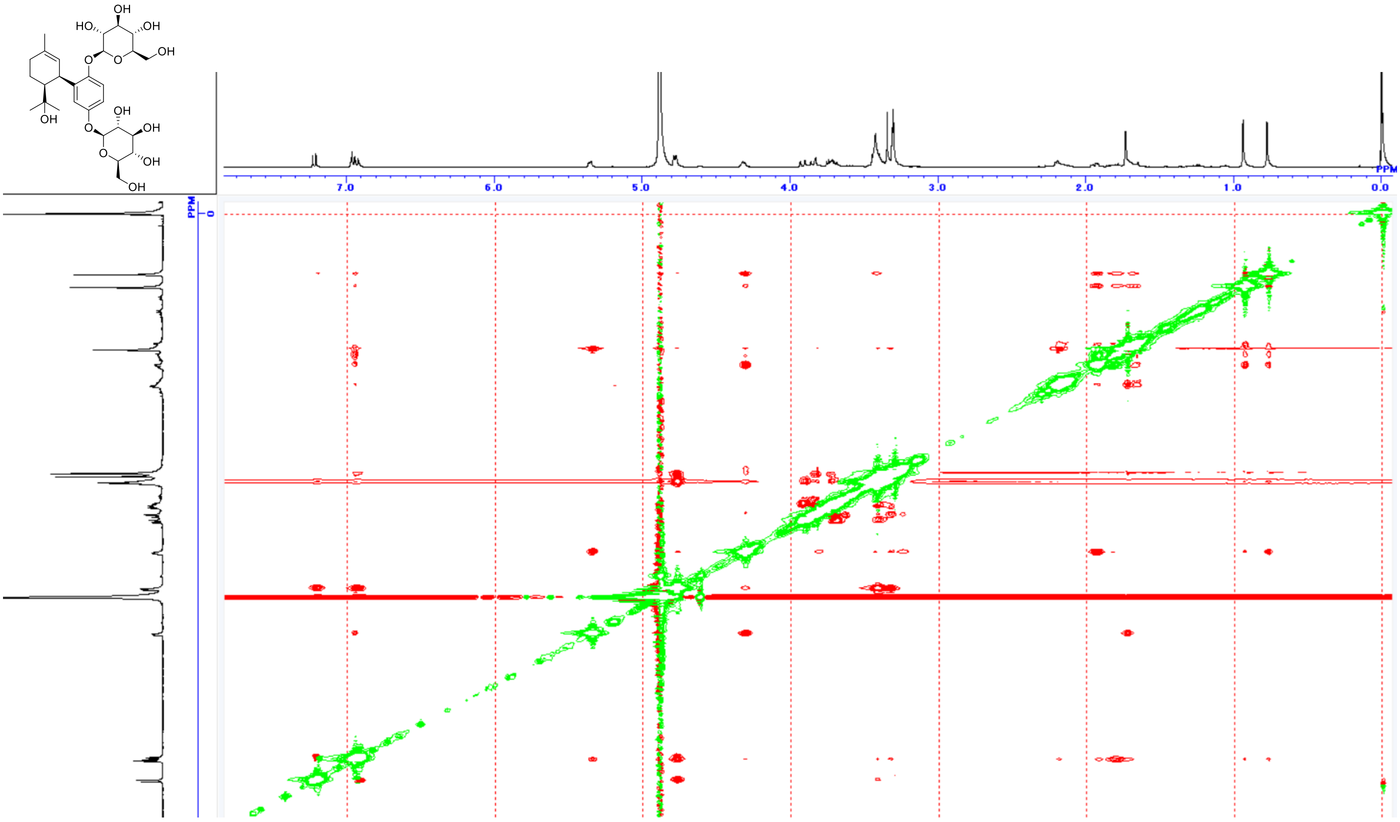

Figure S32 NOESY spectra of compound **4** (in methanol-d_4).

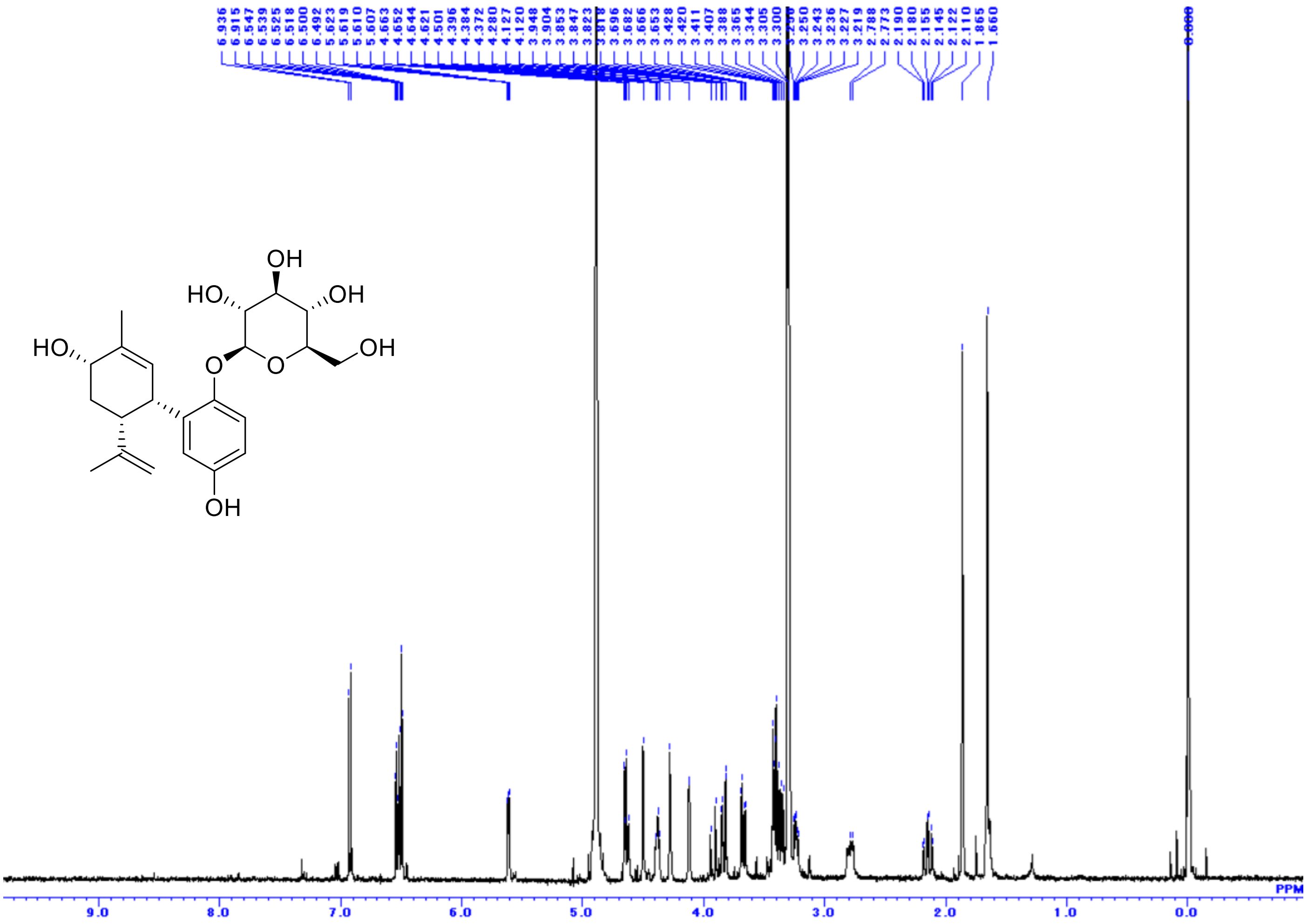

Figure S33 ^{1}H NMR spectrum of compound **5** (in methanol-d_4).

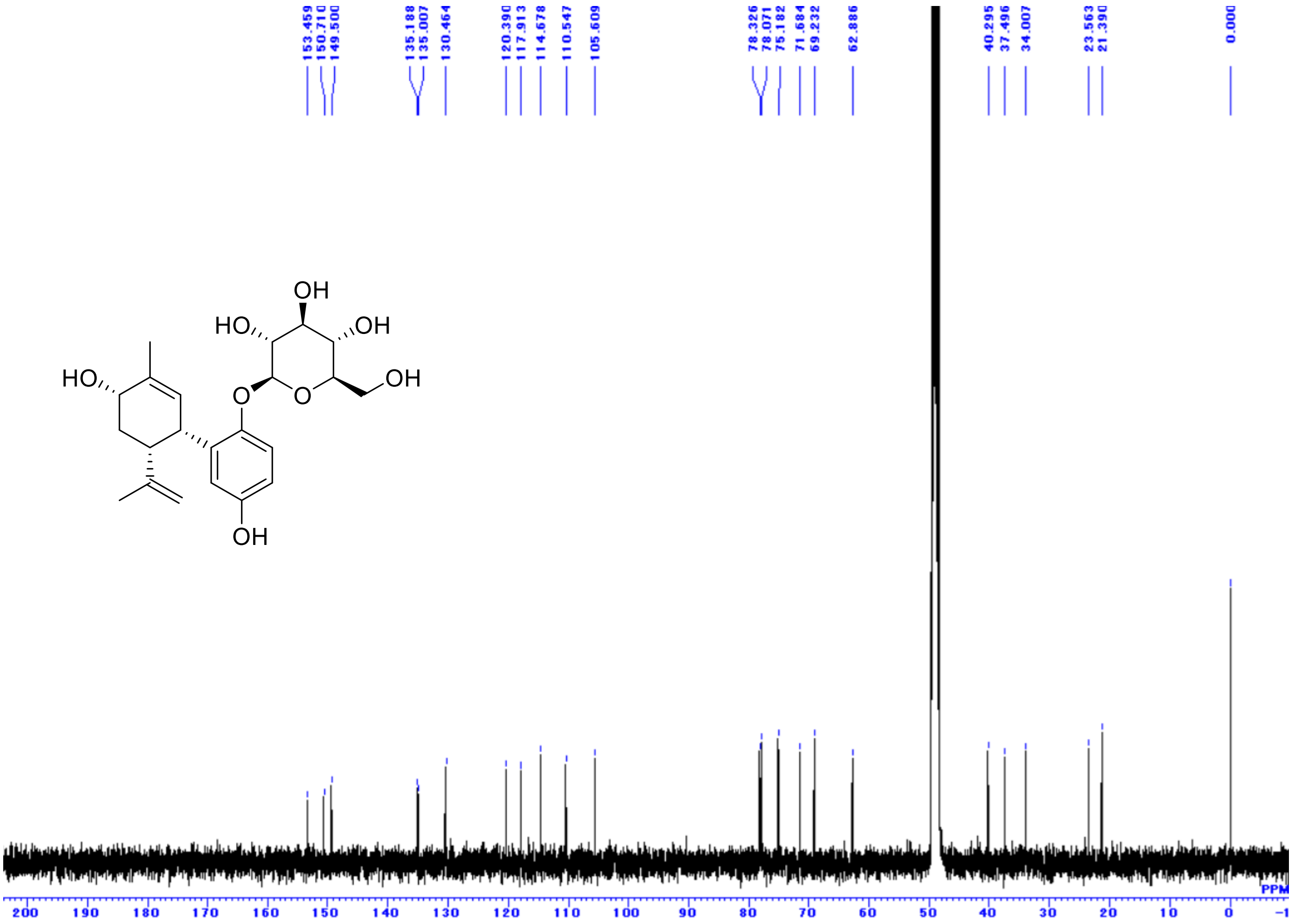

Figure S34 ^{13}C NMR spectrum of compound **5** (in methanol-*d*$_4$).

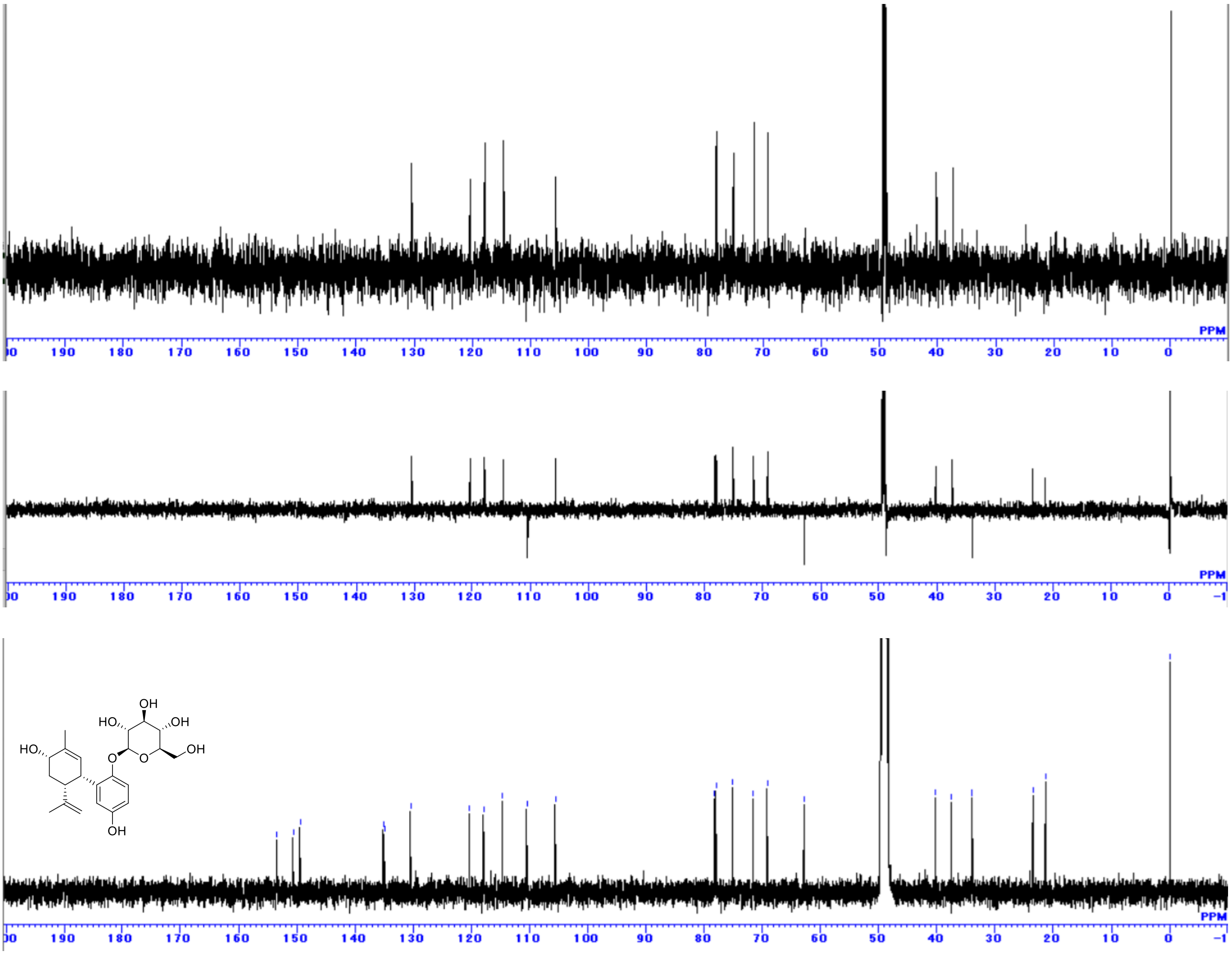

Figure S35 DEPT spectrum of compound **5** (in methanol-*d*$_4$).

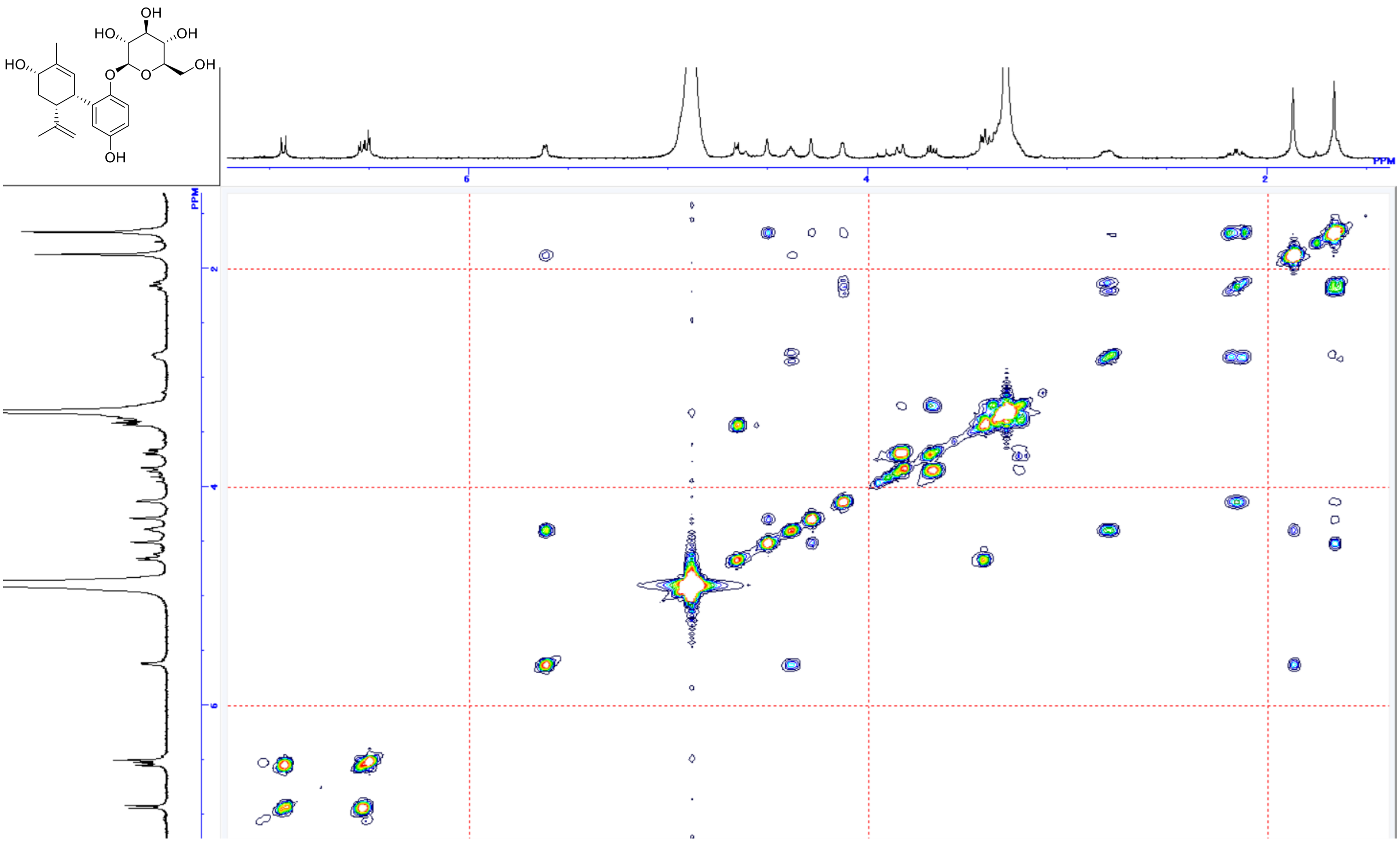

Figure S36 ^{1}H-^{1}H COSY spectrum of compound **5** (in methanol-d_4).

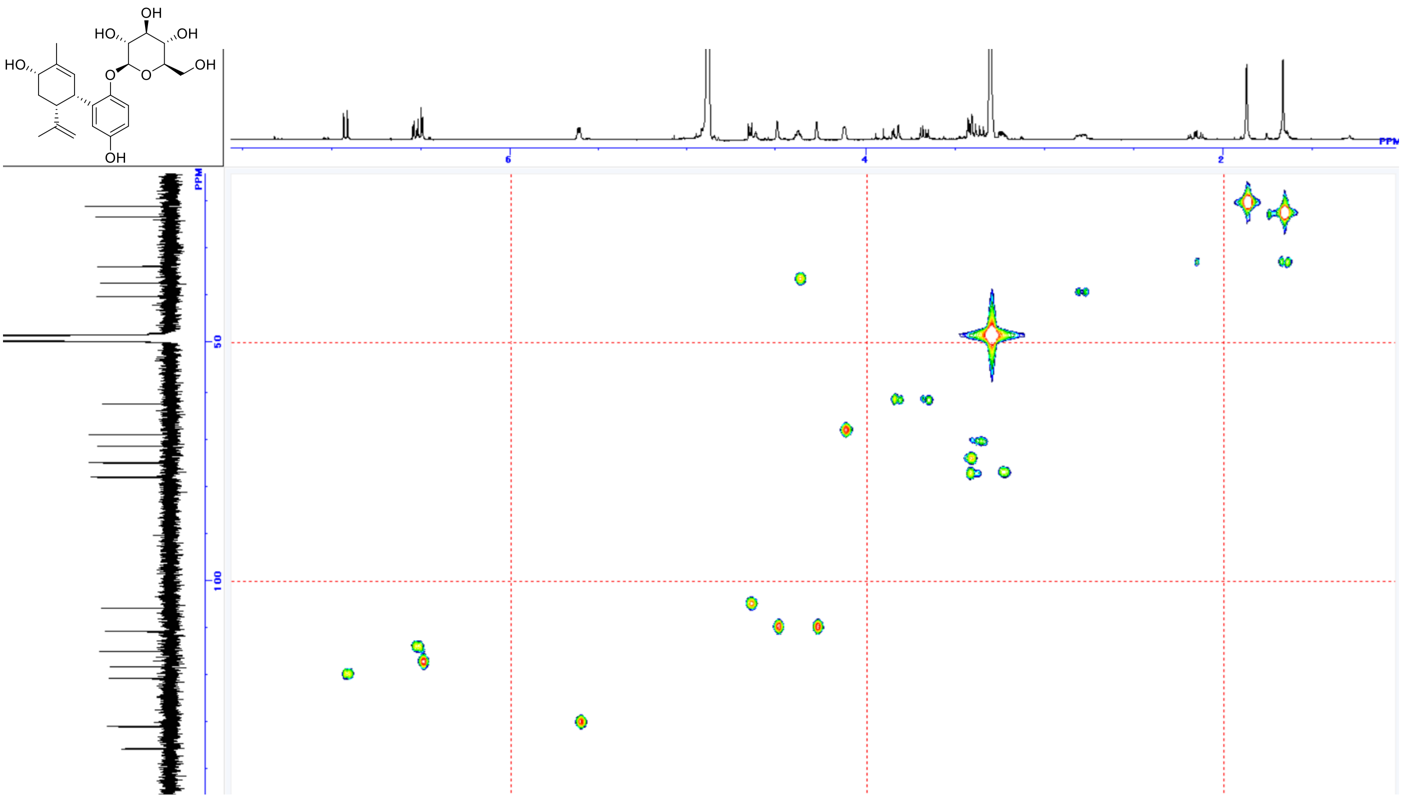

Figure S37 HMQC spectrum of compound **5** (in methanol-d_4).

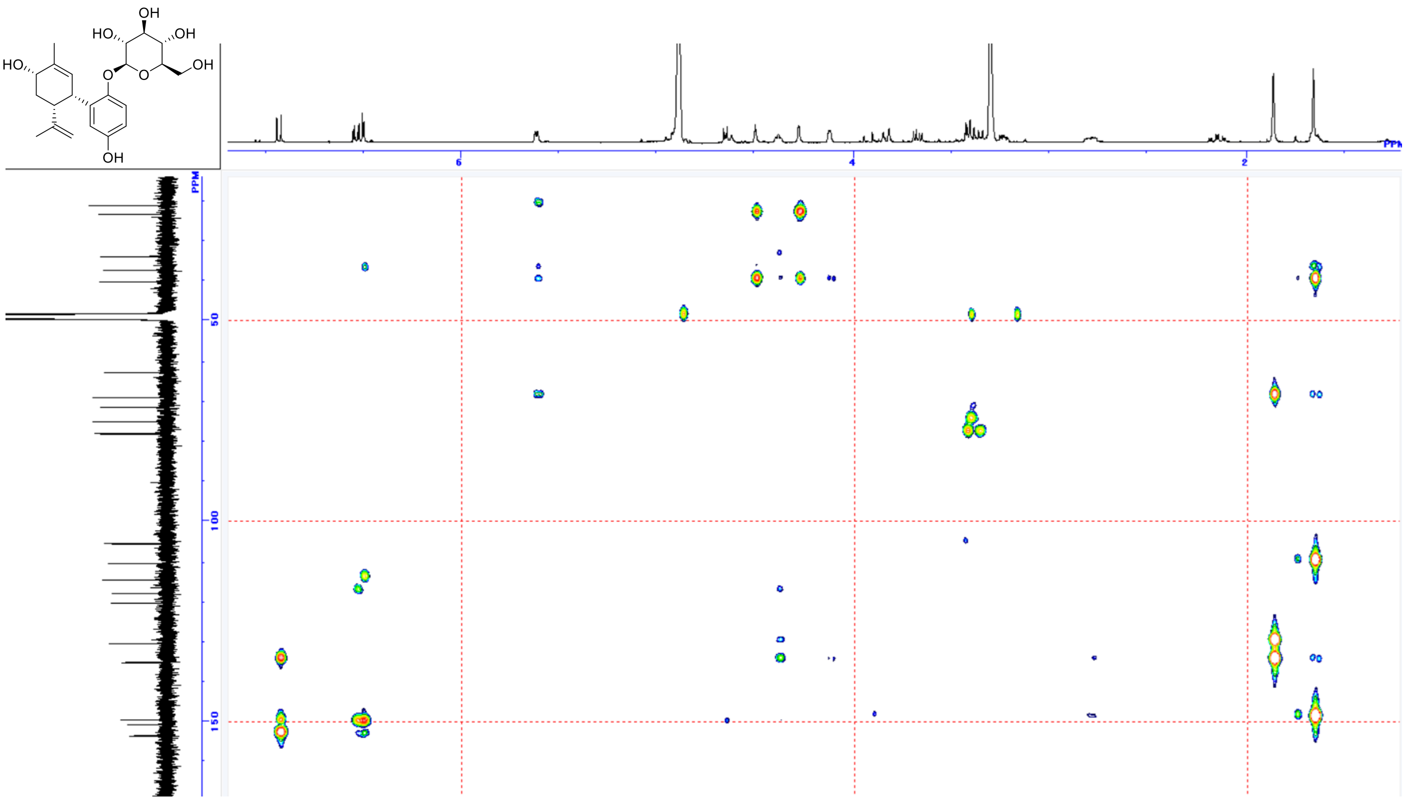

Figure S38 HMBC spectrum of compound **5** (in methanol-*d*4).

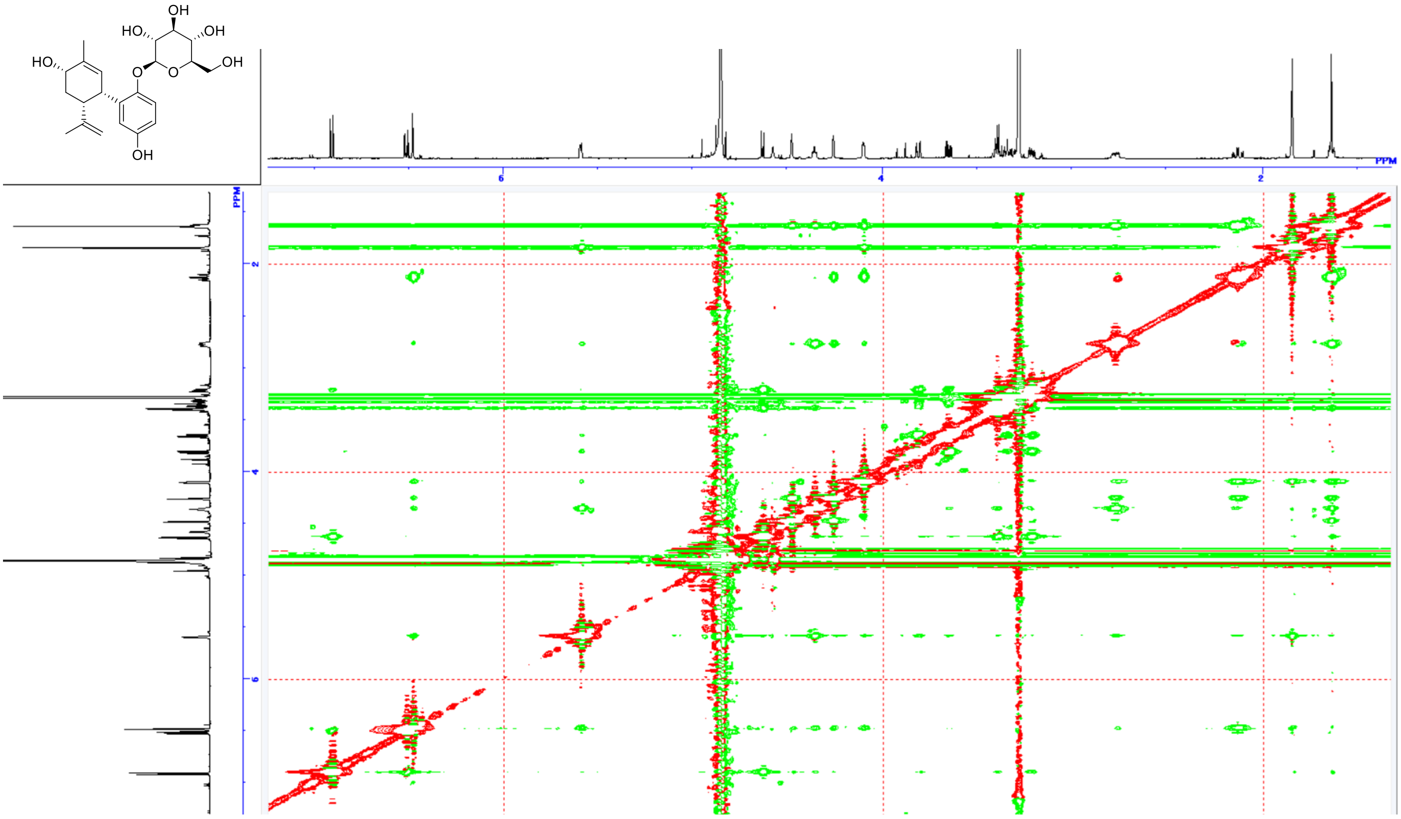

Figure S39 NOESY spectra of compound **5** (in methanol-*d*4).

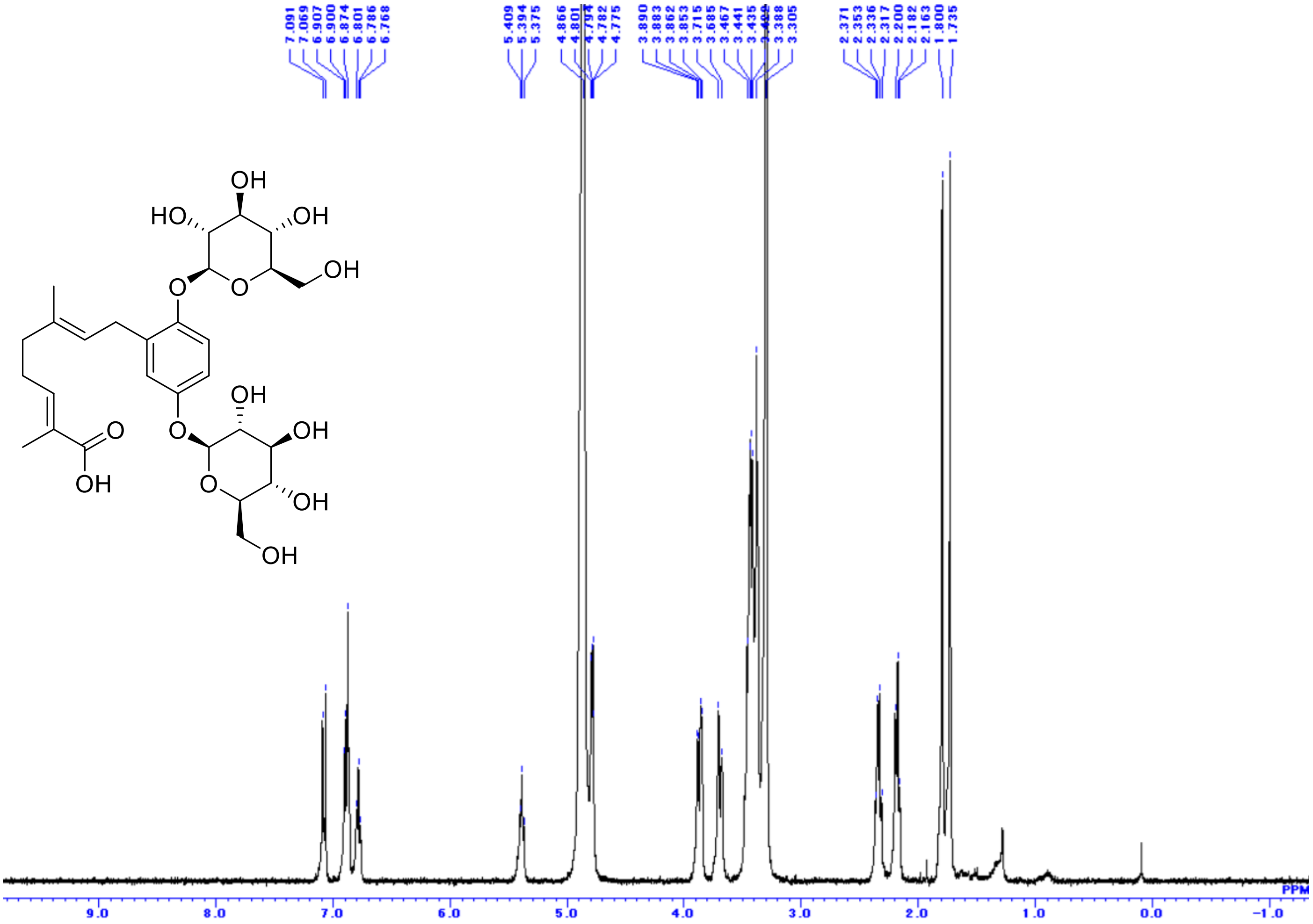

Figure S40 ^{1}H NMR spectrum of compound **6** (in methanol-d_4).

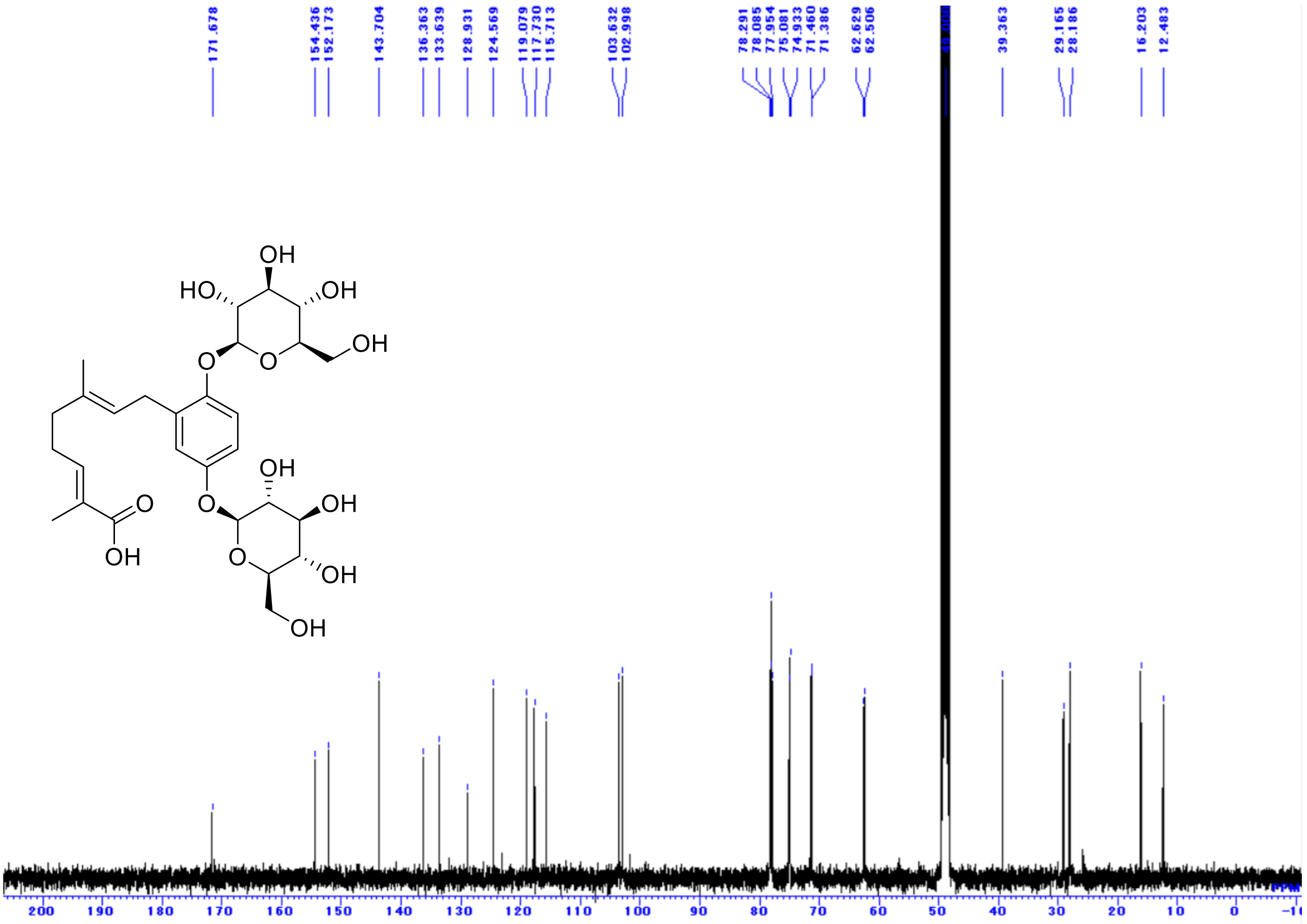

Figure S41 ^{13}C NMR spectrum of compound **6** (in methanol-d_4).

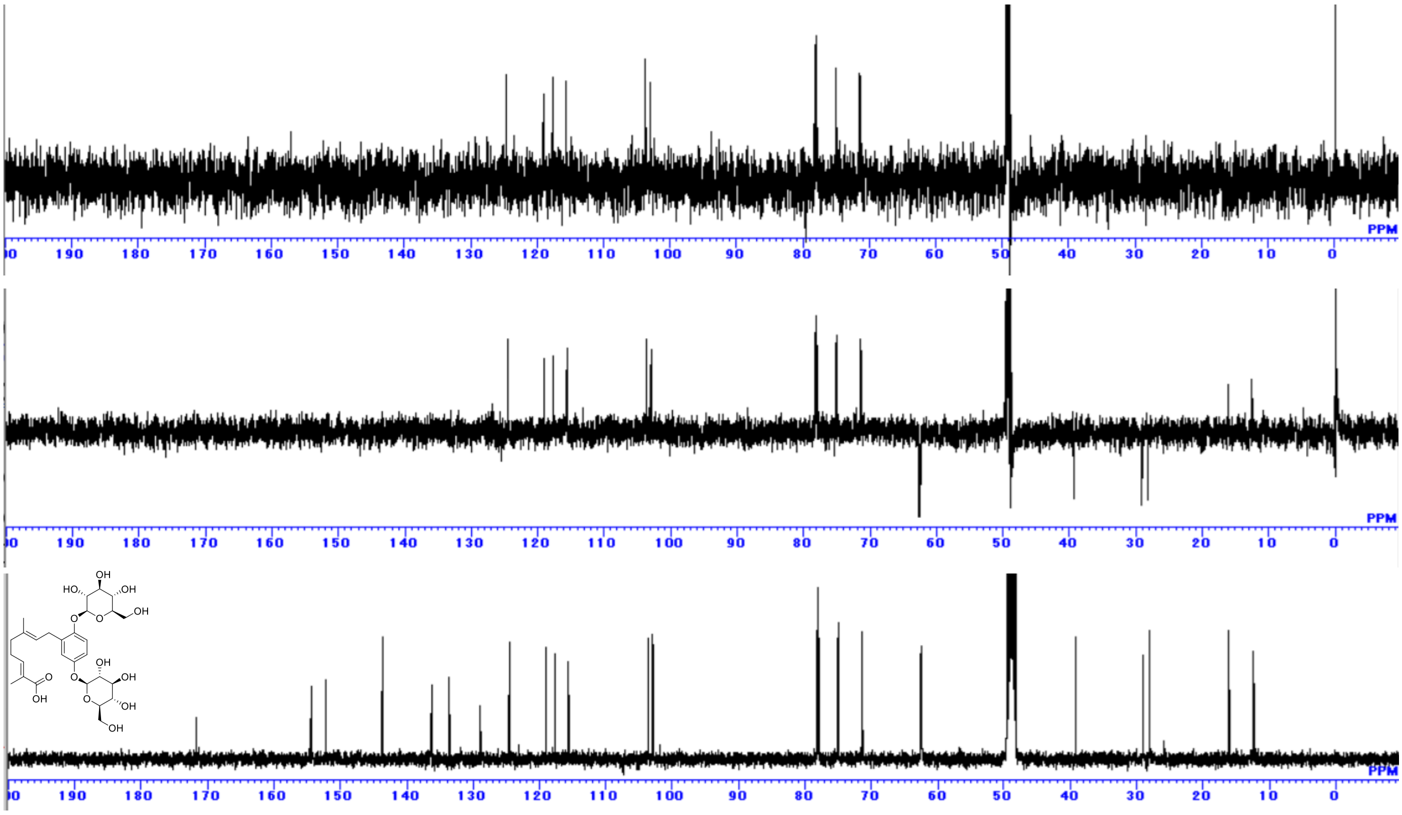

Figure S42 DEPT spectrum of compound **6** (in methanol-d_4).

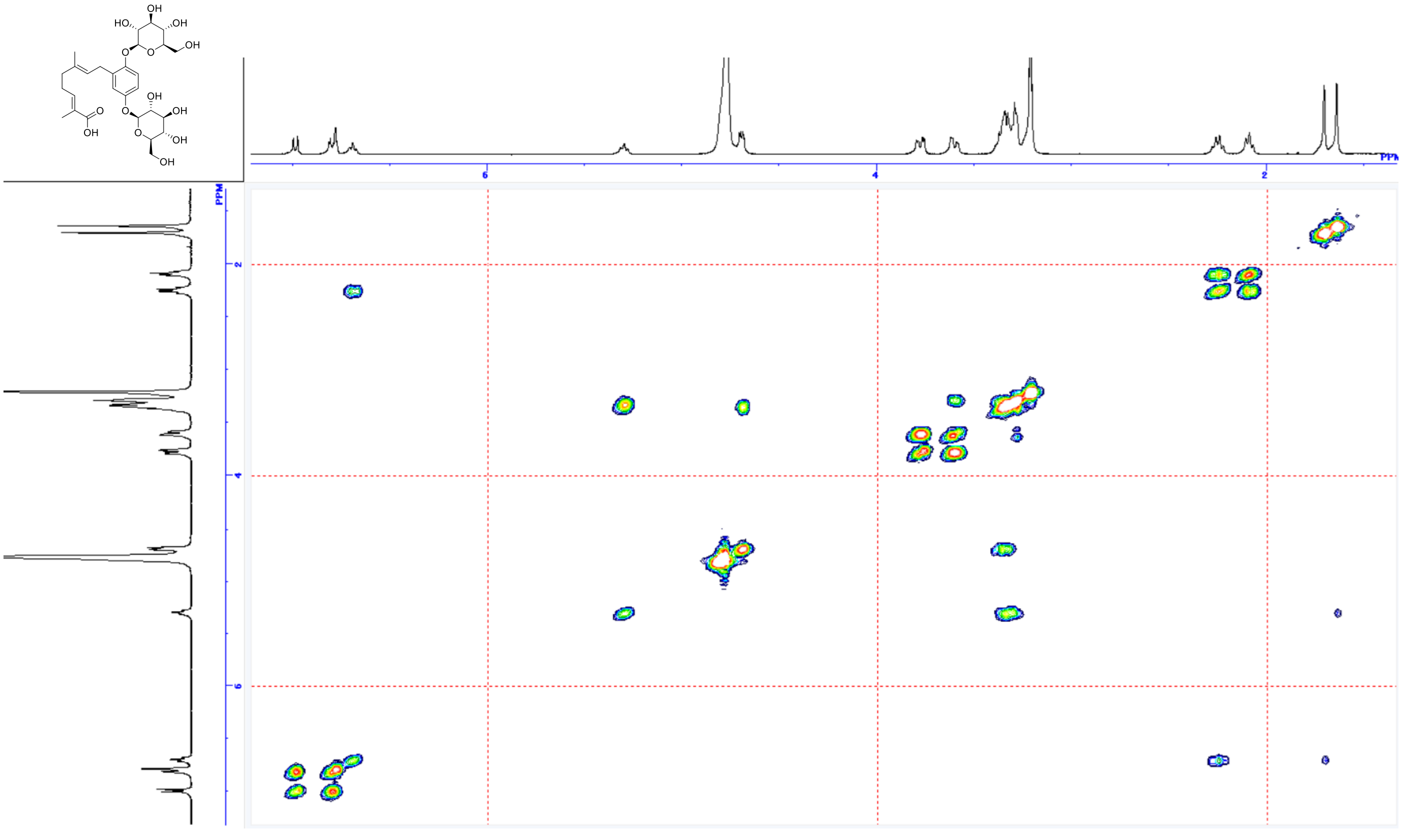

Figure S43 ^{1}H-^{1}H COSY spectrum of compound **6** (in methanol-d_4).

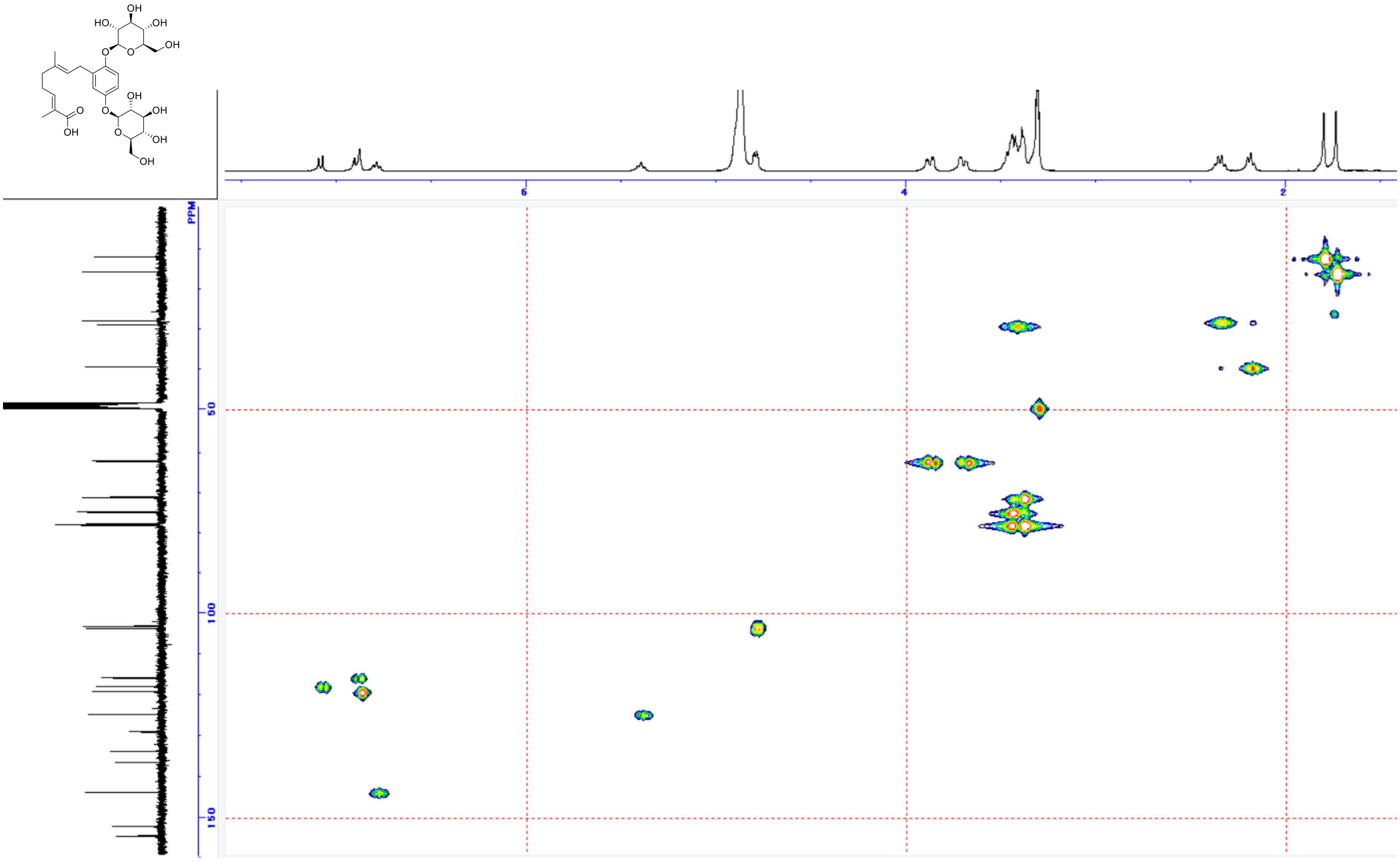

Figure S44 HMQC spectrum of compound **6** (in methanol-d_4).

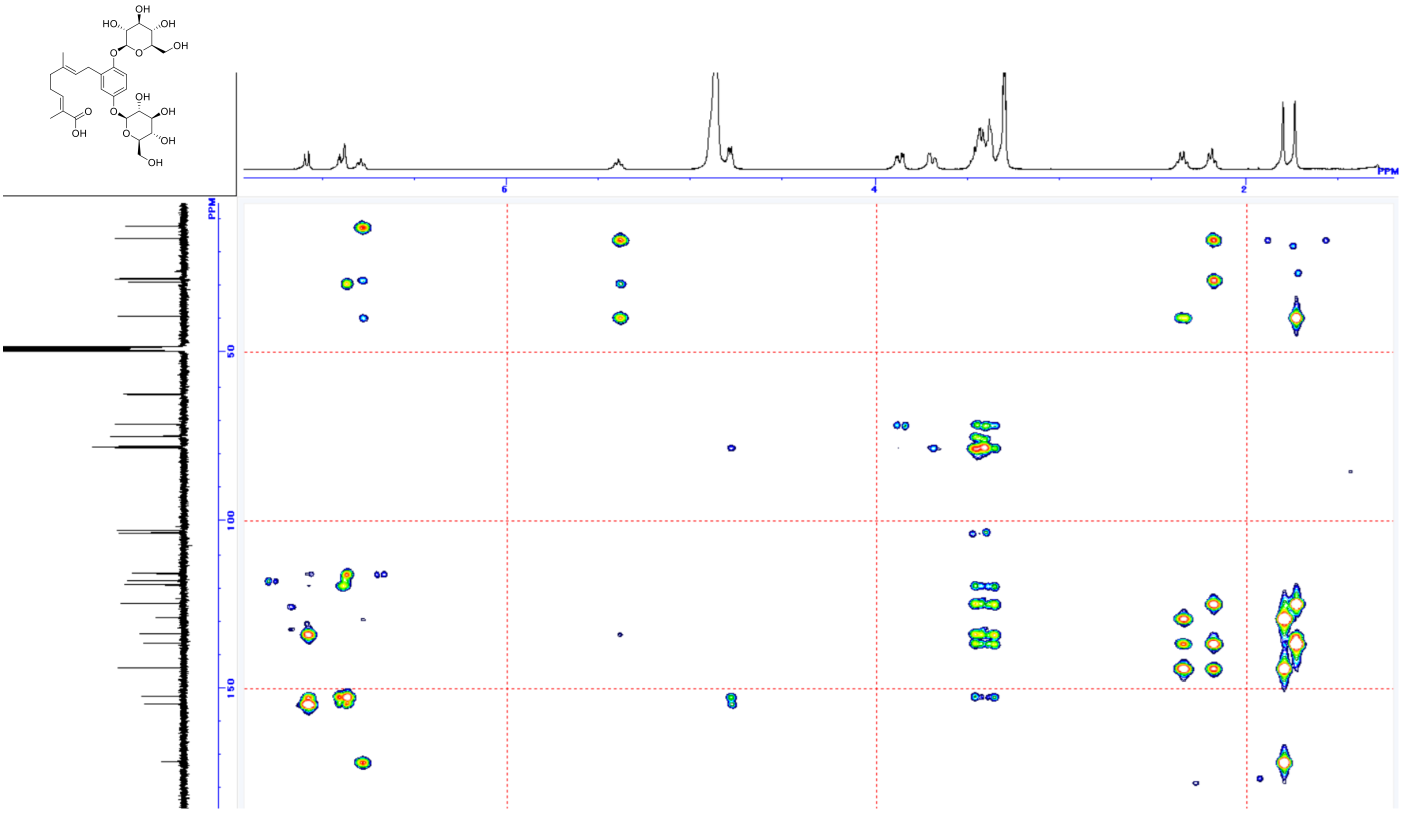

Figure S45 HMBC spectrum of compound **6** (in methanol-d_4).

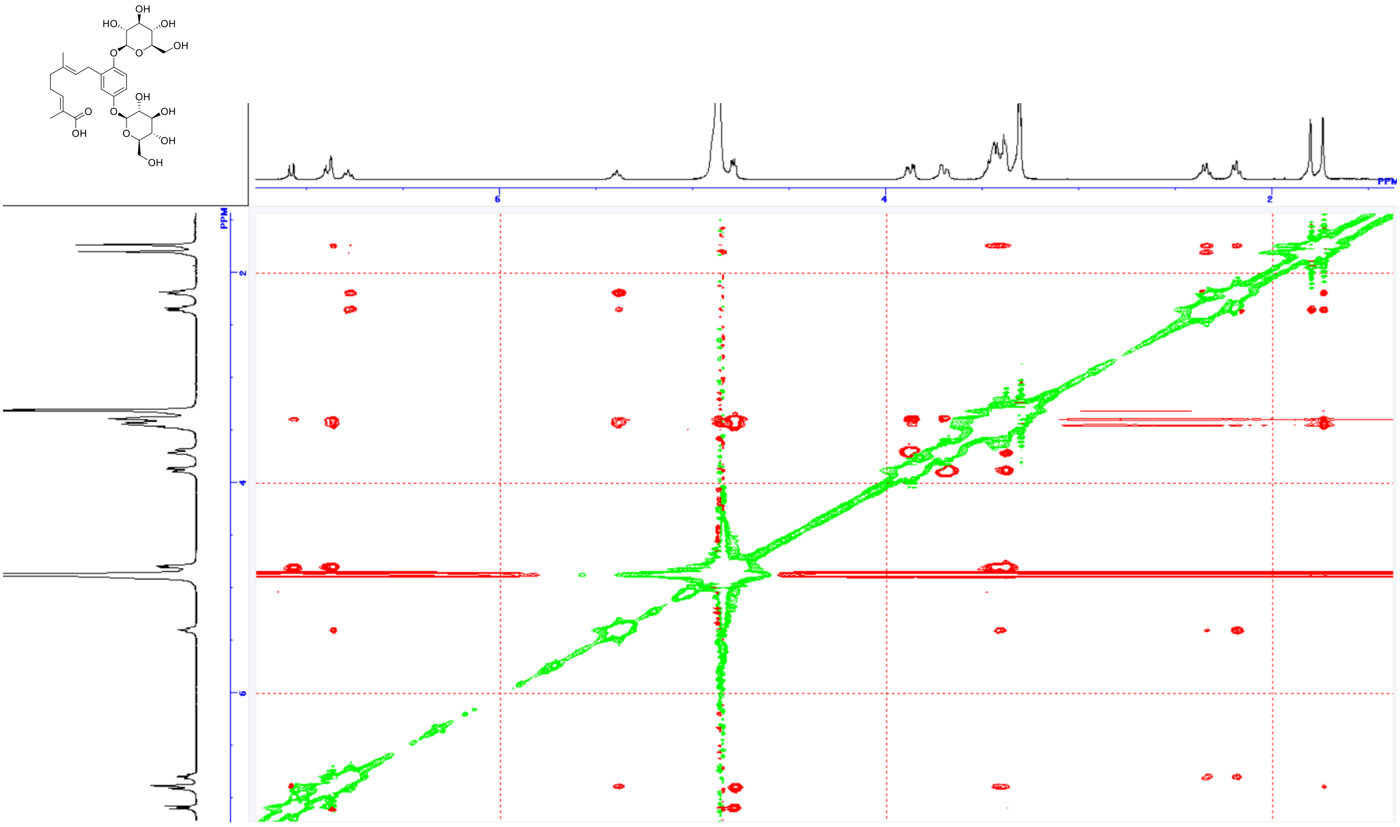

Figure S46 NOESY spectra of compound **6** (in methanol-d_4).

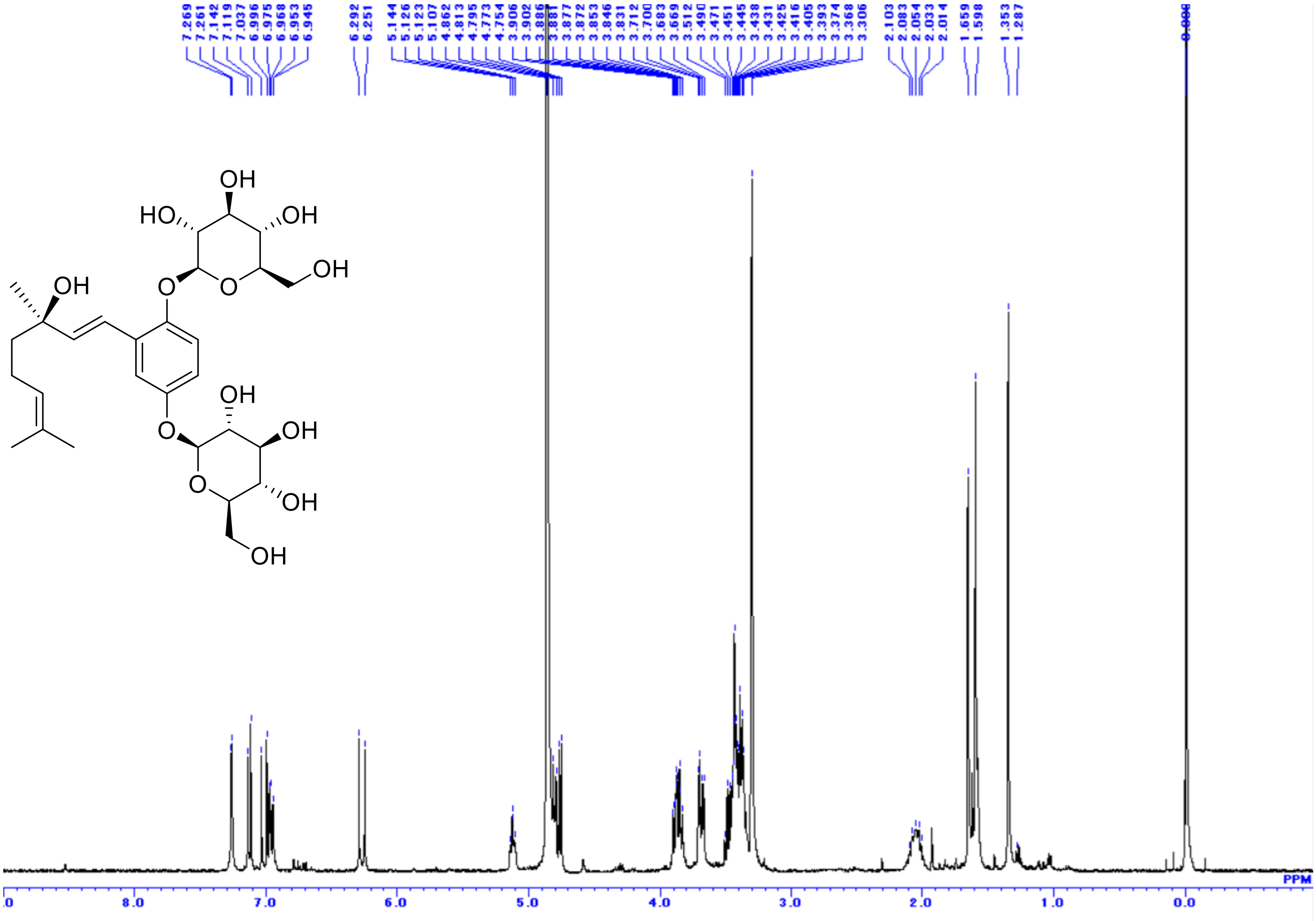

Figure S47 ^{1}H NMR spectrum of compound **7** (in methanol-d_4).

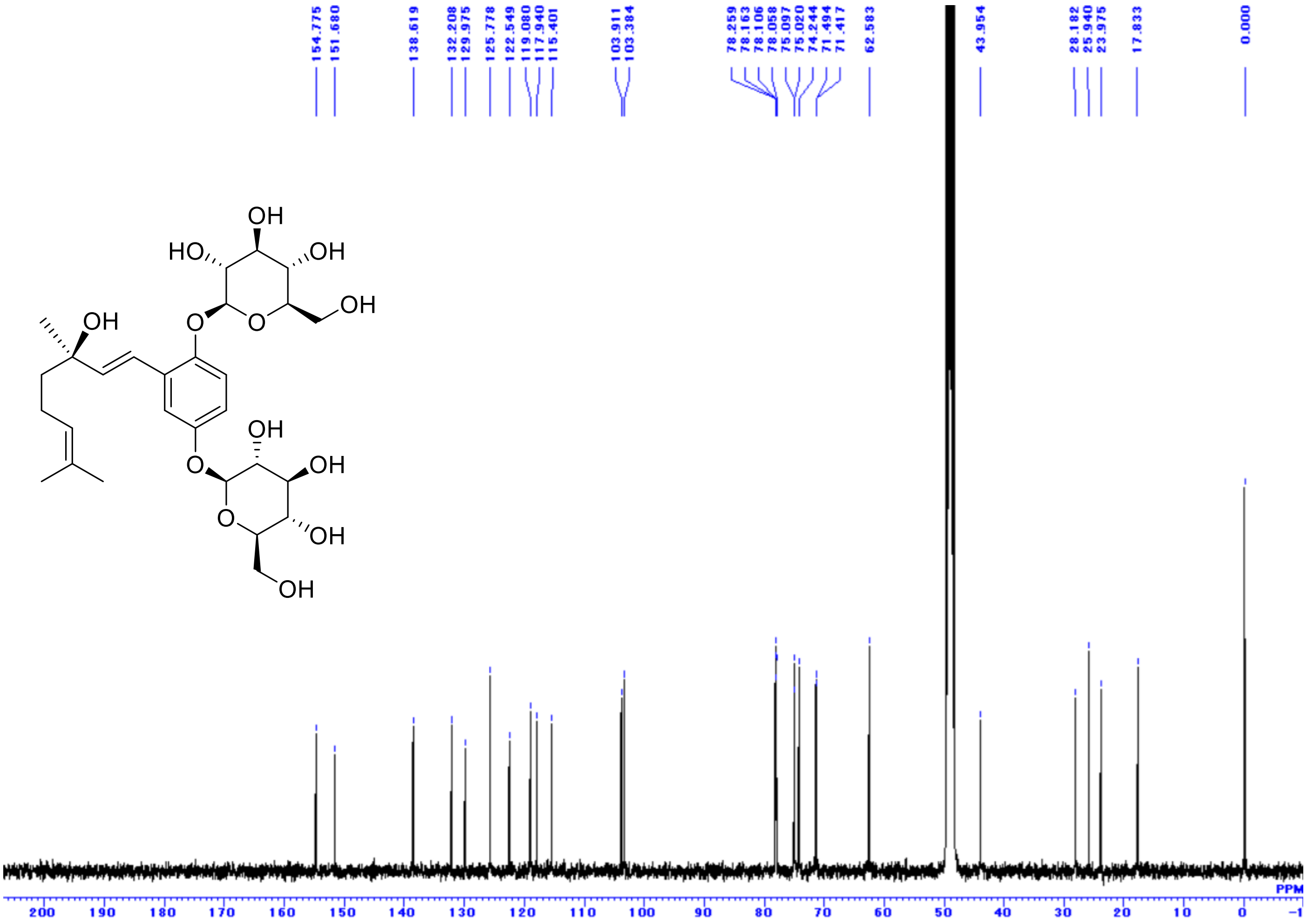

Figure S48 ^{13}C NMR spectrum of compound **7** (in methanol-d_4).

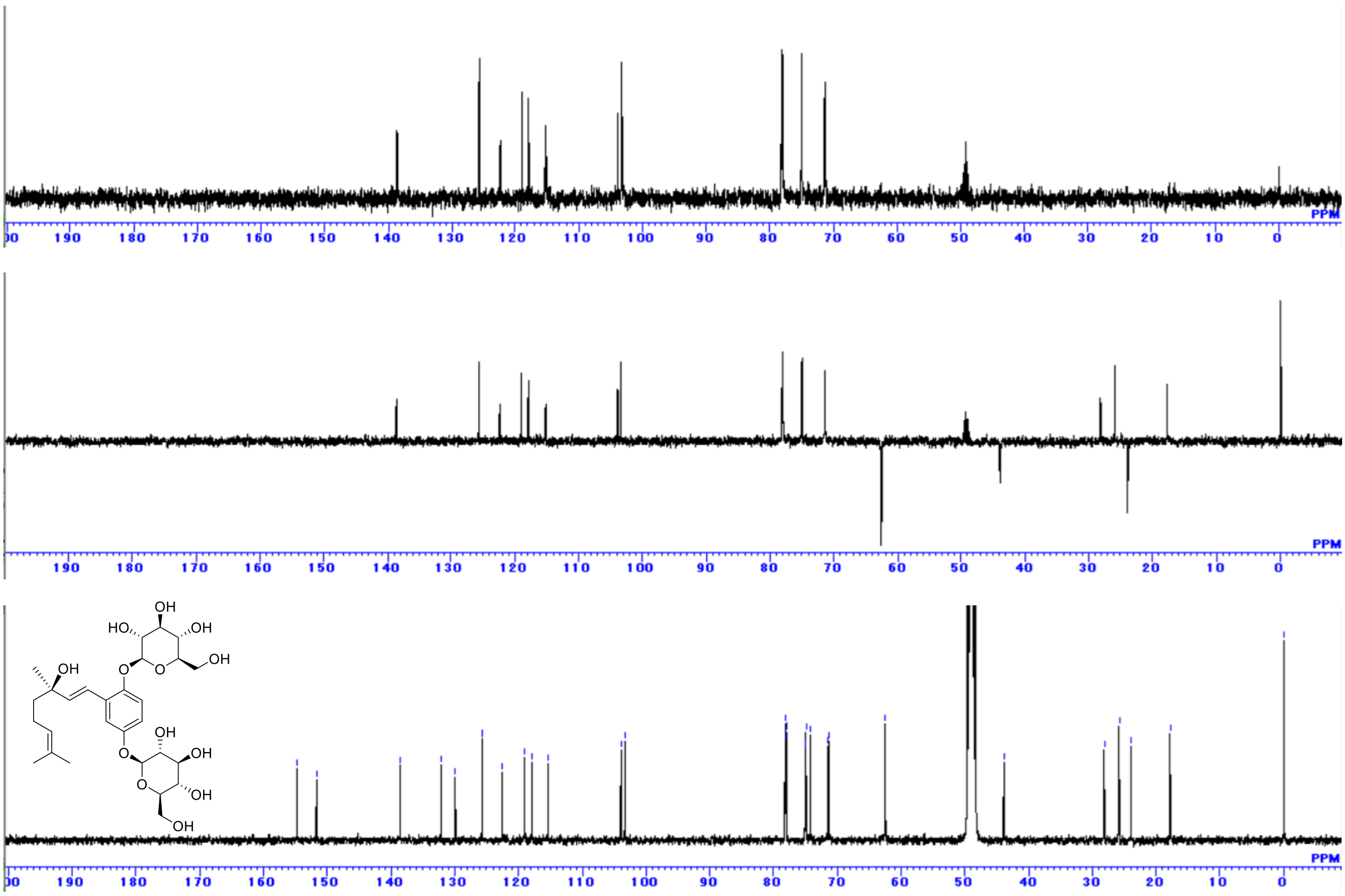

Figure S49 DEPT spectrum of compound **7** (in methanol-*d*4).

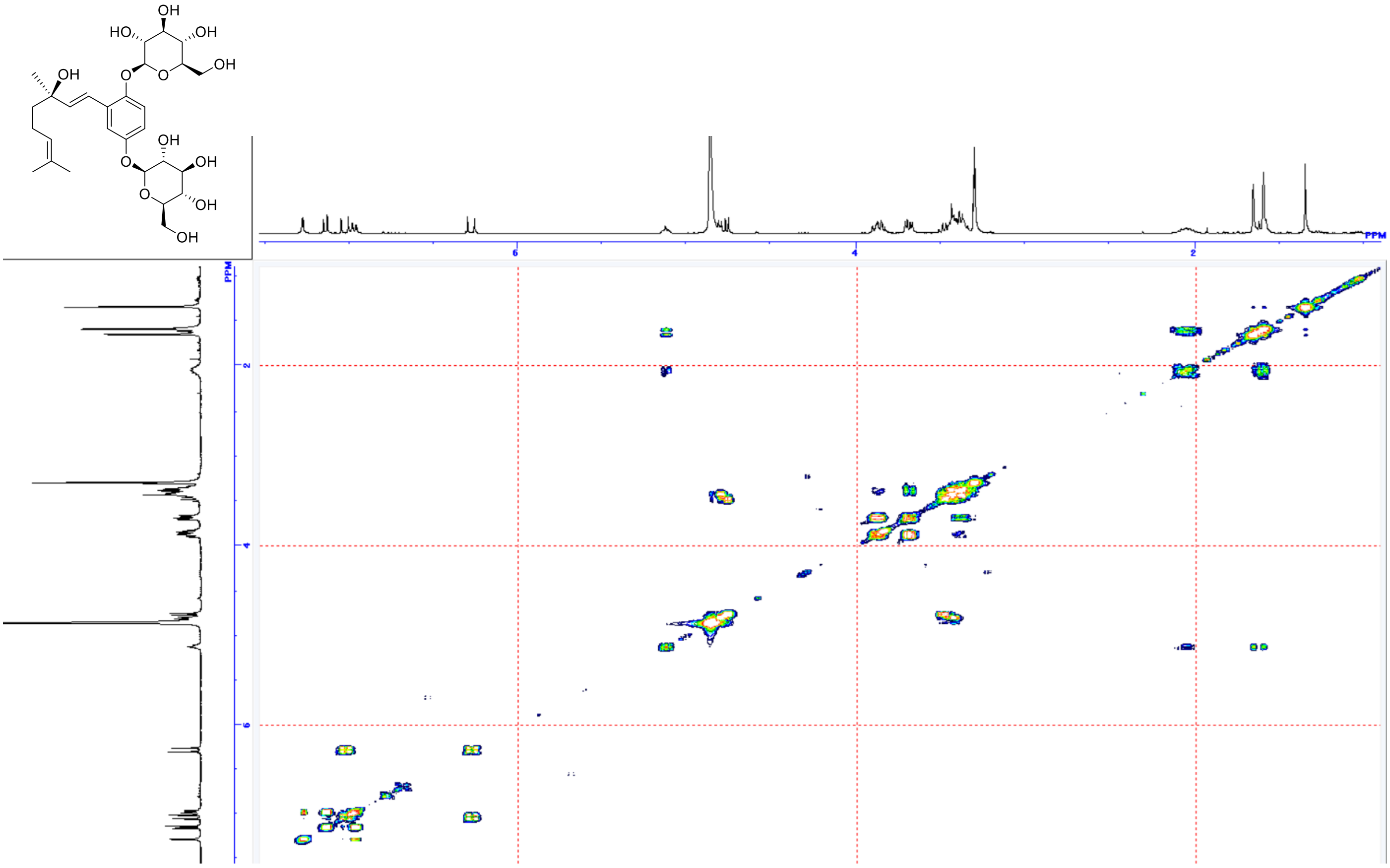

Figure S50 ^{1}H-^{1}H COSY spectrum of compound **7** (in methanol-d_4).

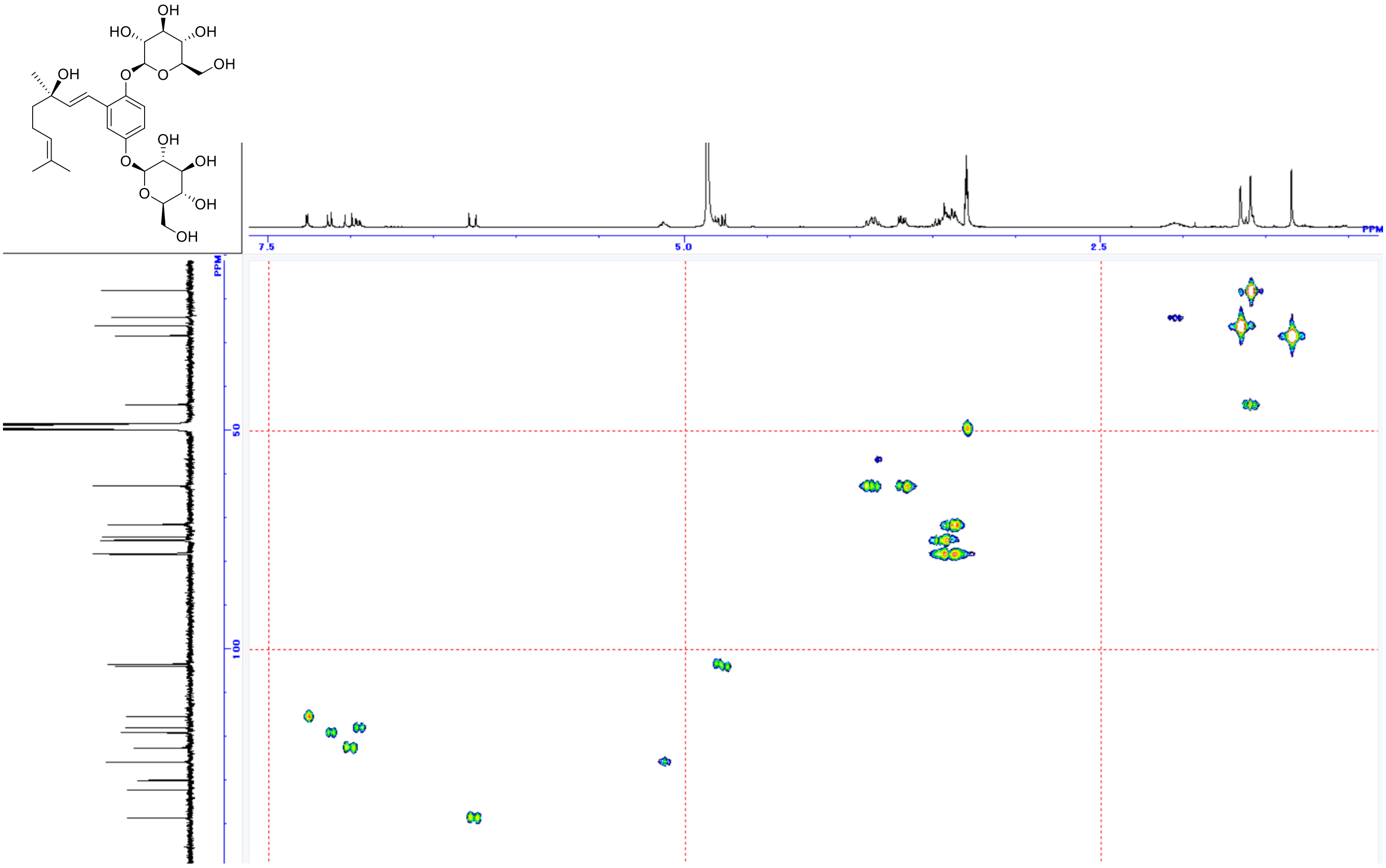

Figure S51 HMQC spectrum of compound **7** (in methanol-d_4).

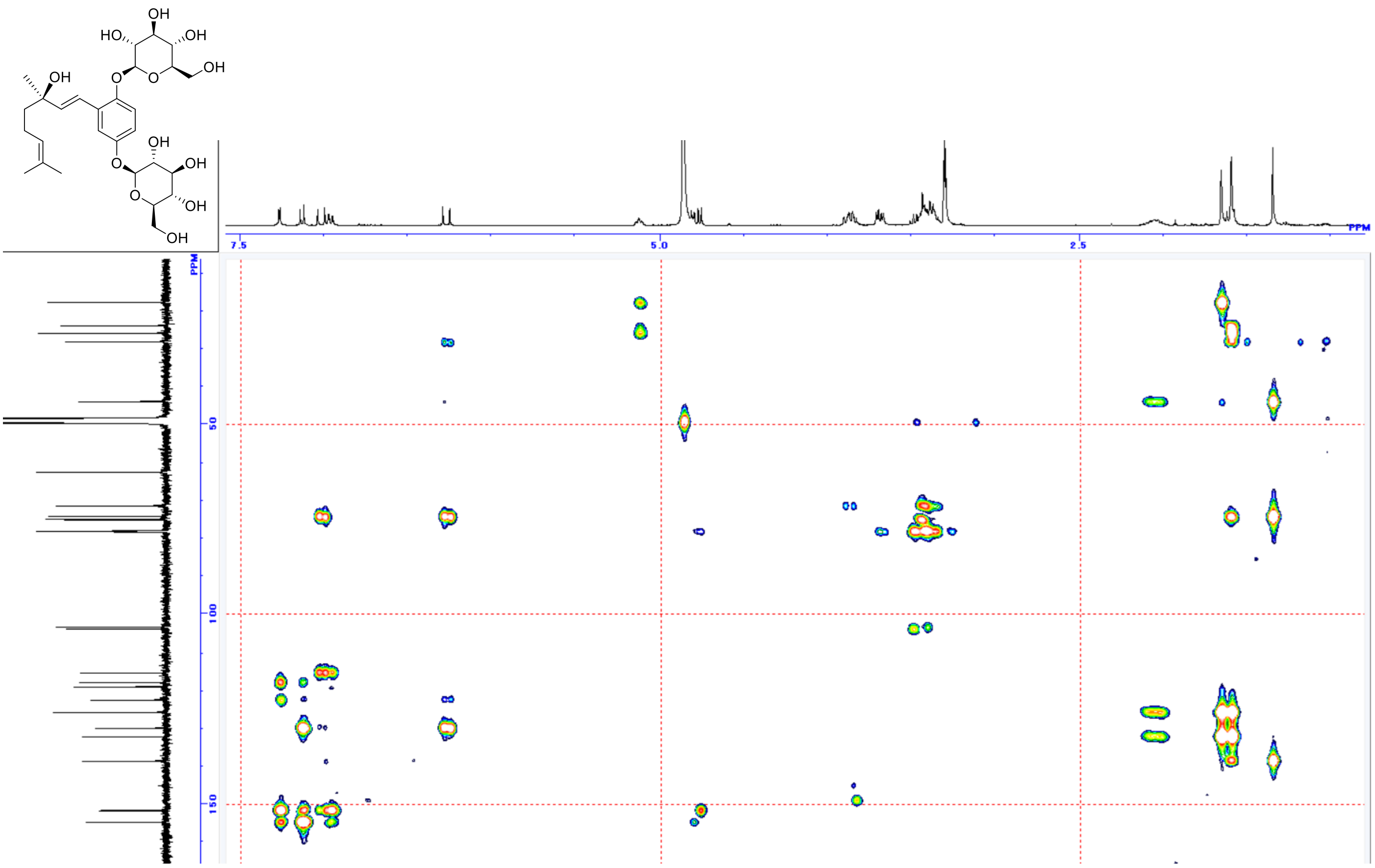

Figure S52 HMBC spectrum of compound **7** (in methanol-d_4).

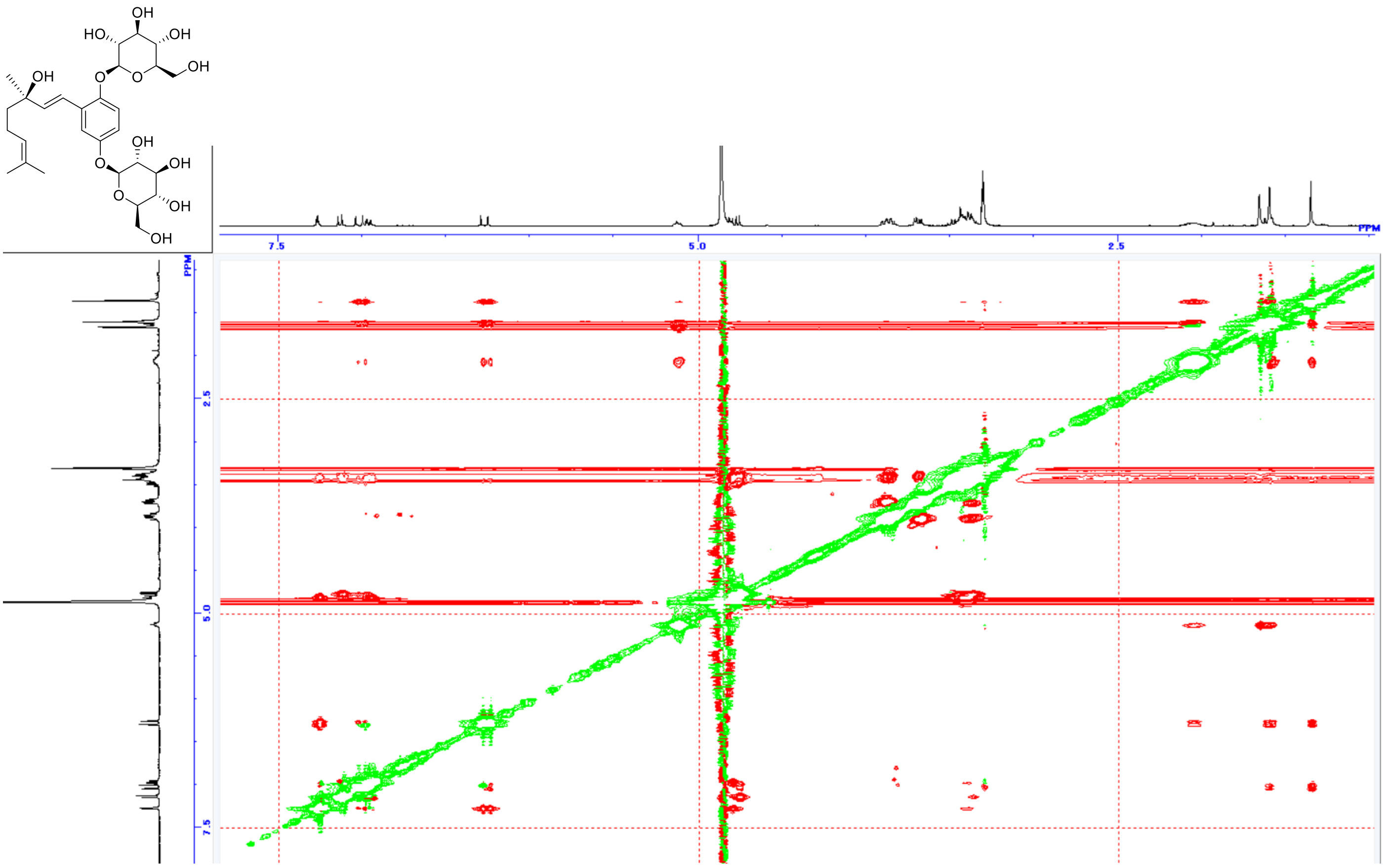

Figure S53 NOESY spectra of compound **7** (in methanol-d_4).

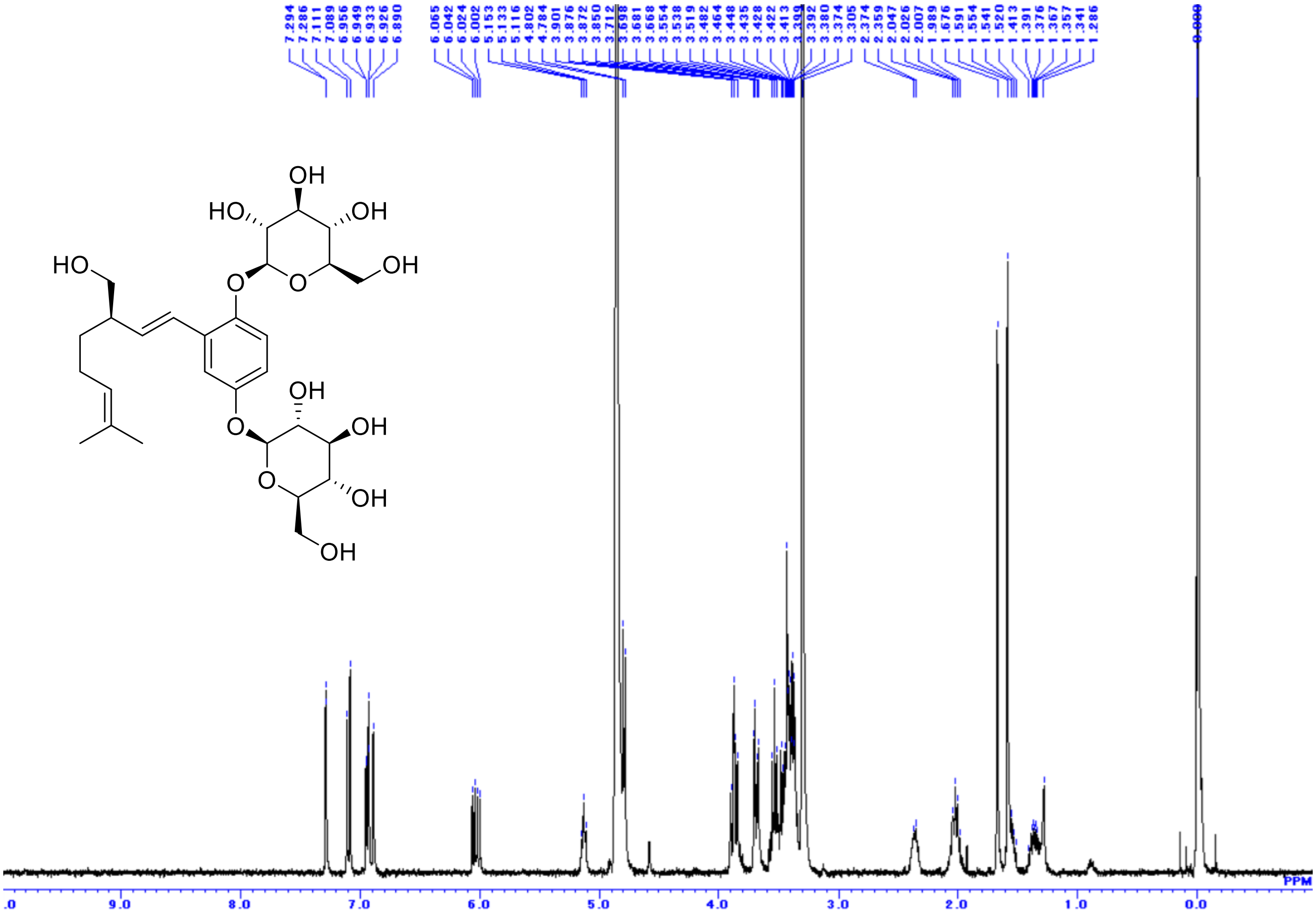

Figure S54 ^{1}H NMR spectrum of compound **8** (in methanol-d_4).

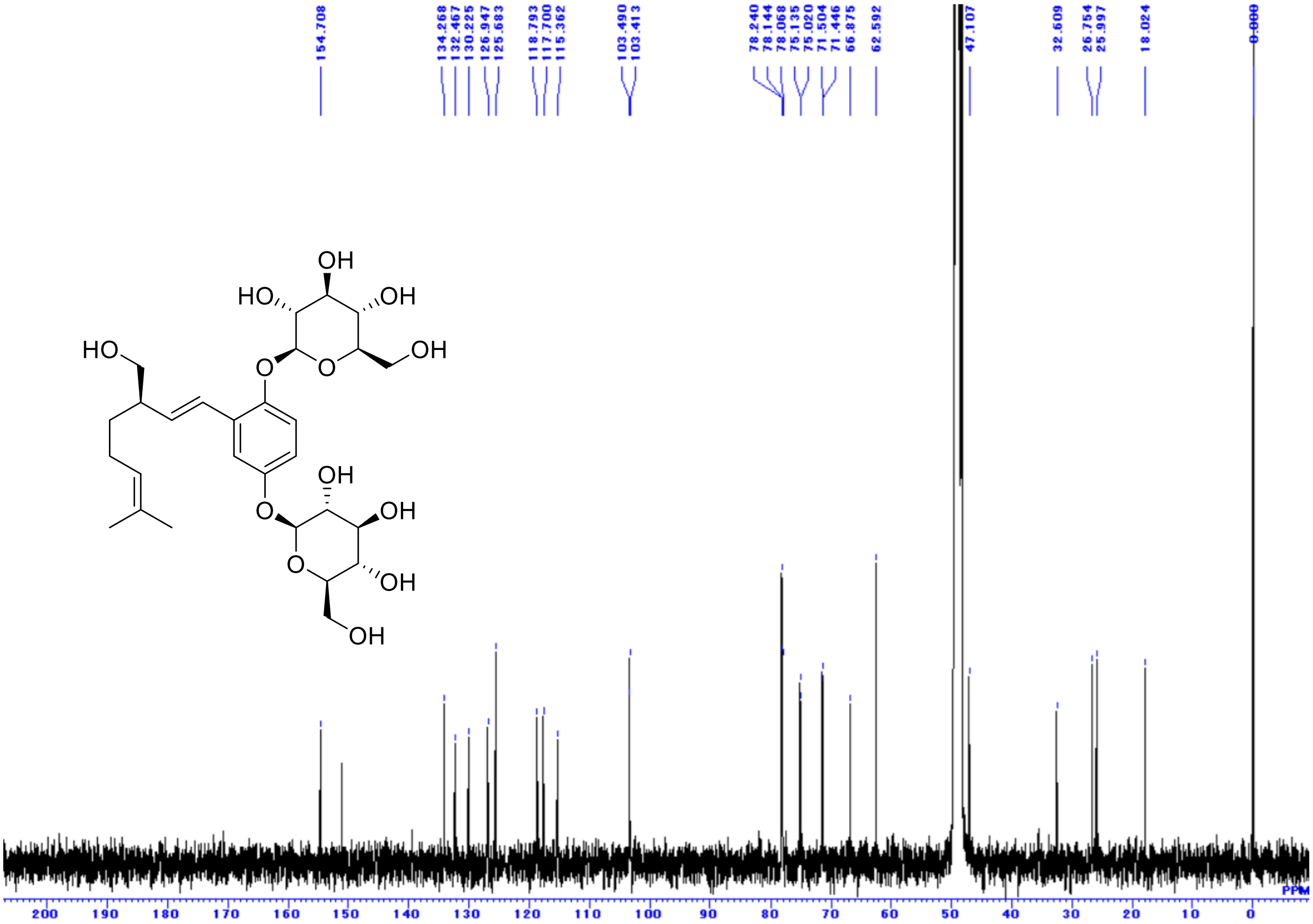

Figure S55 ^{13}C NMR spectrum of compound **8** (in methanol-*d*4).

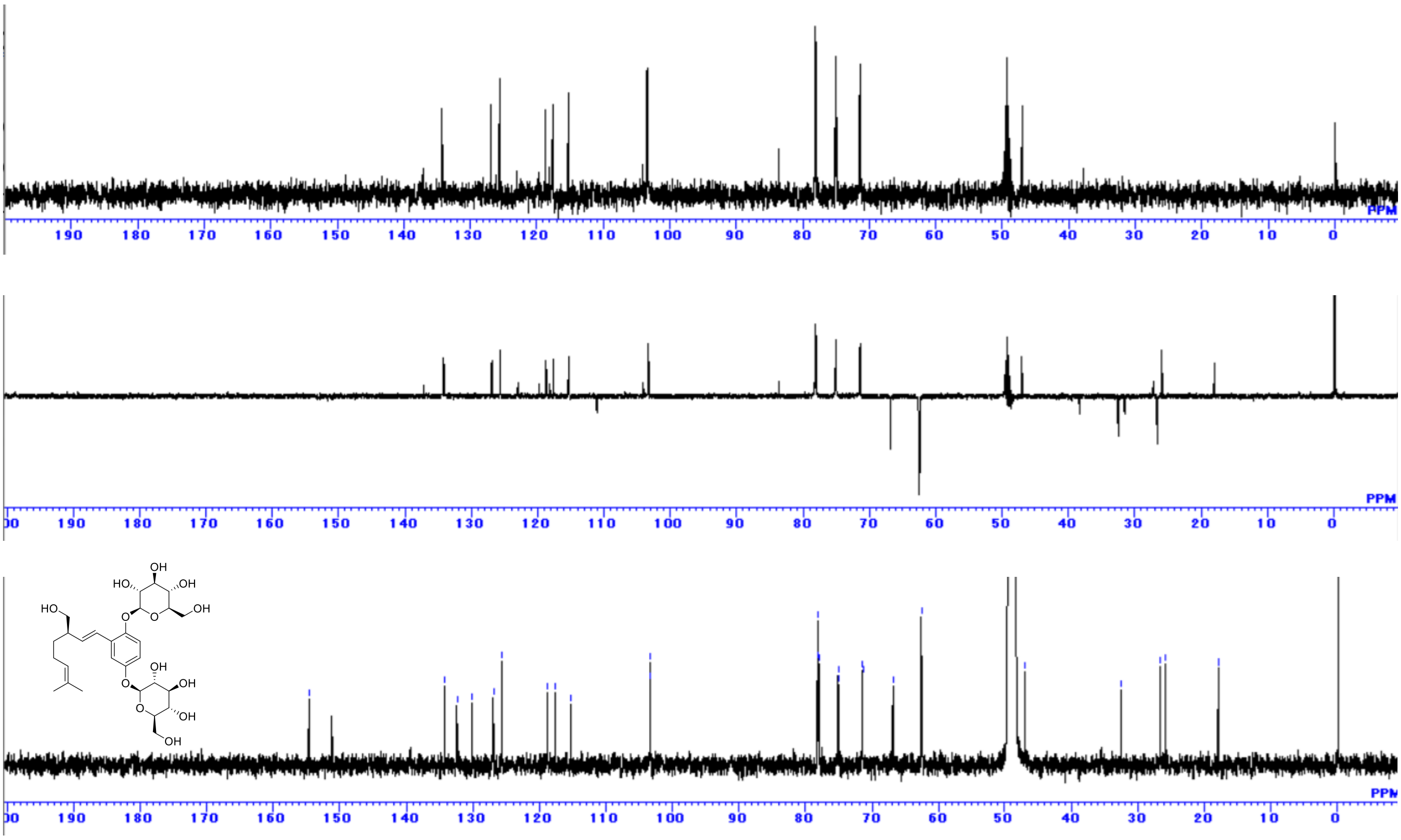

Figure S56 DEPT spectrum of compound **8** (in methanol-*d*4).

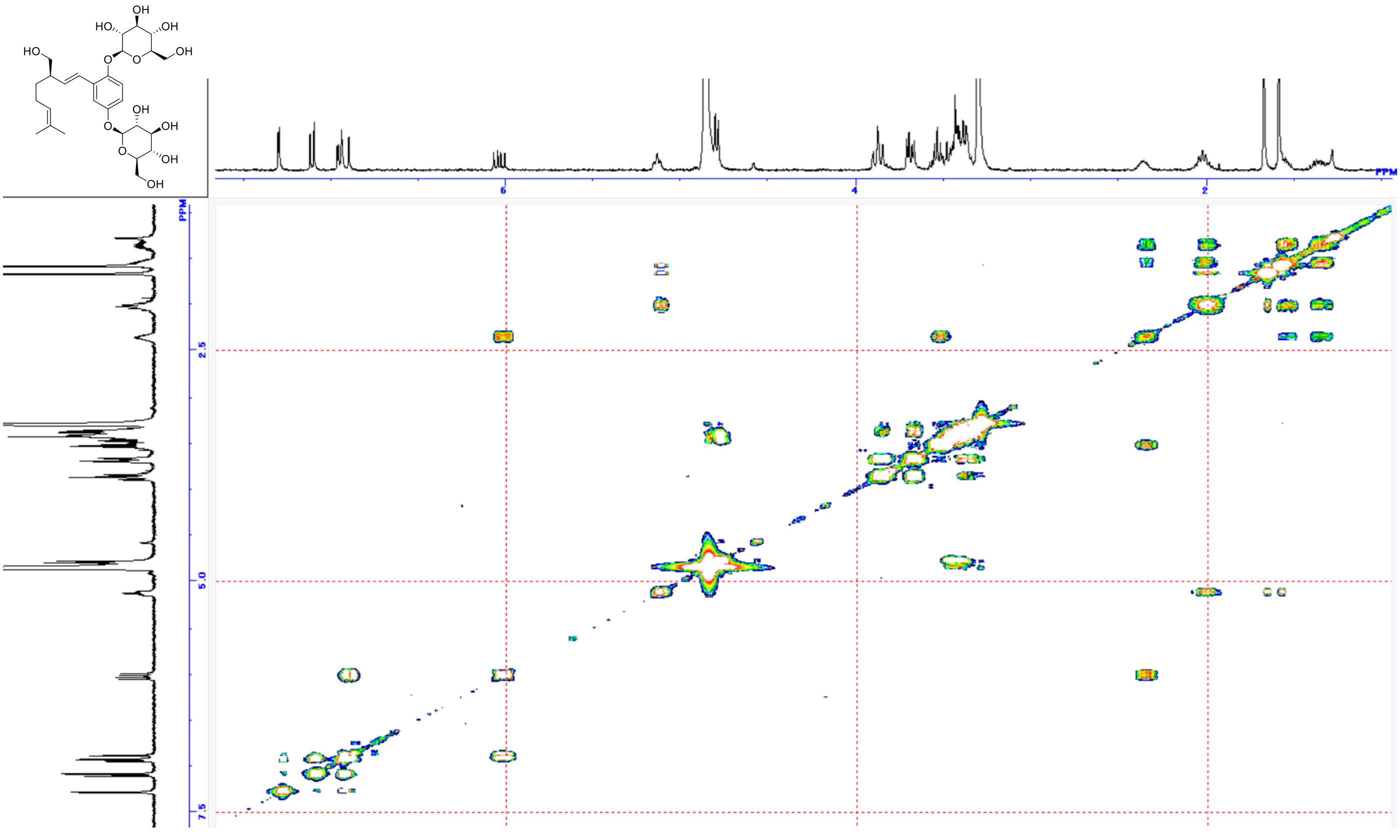

Figure S57 ^{1}H-^{1}H COSY spectrum of compound **8** (in methanol-*d*4).

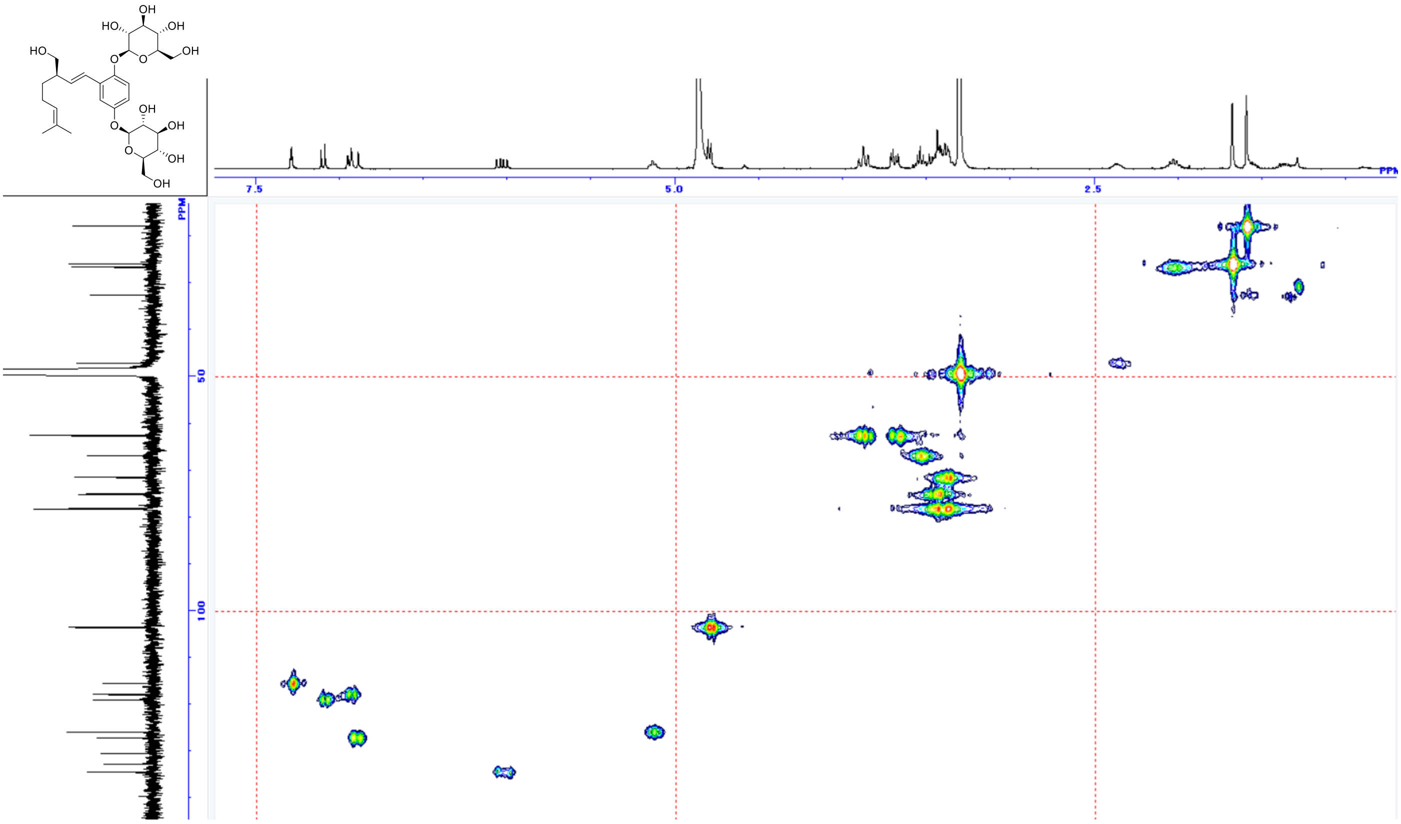

Figure S58 HMQC spectrum of compound **8** (in methanol-*d*4).

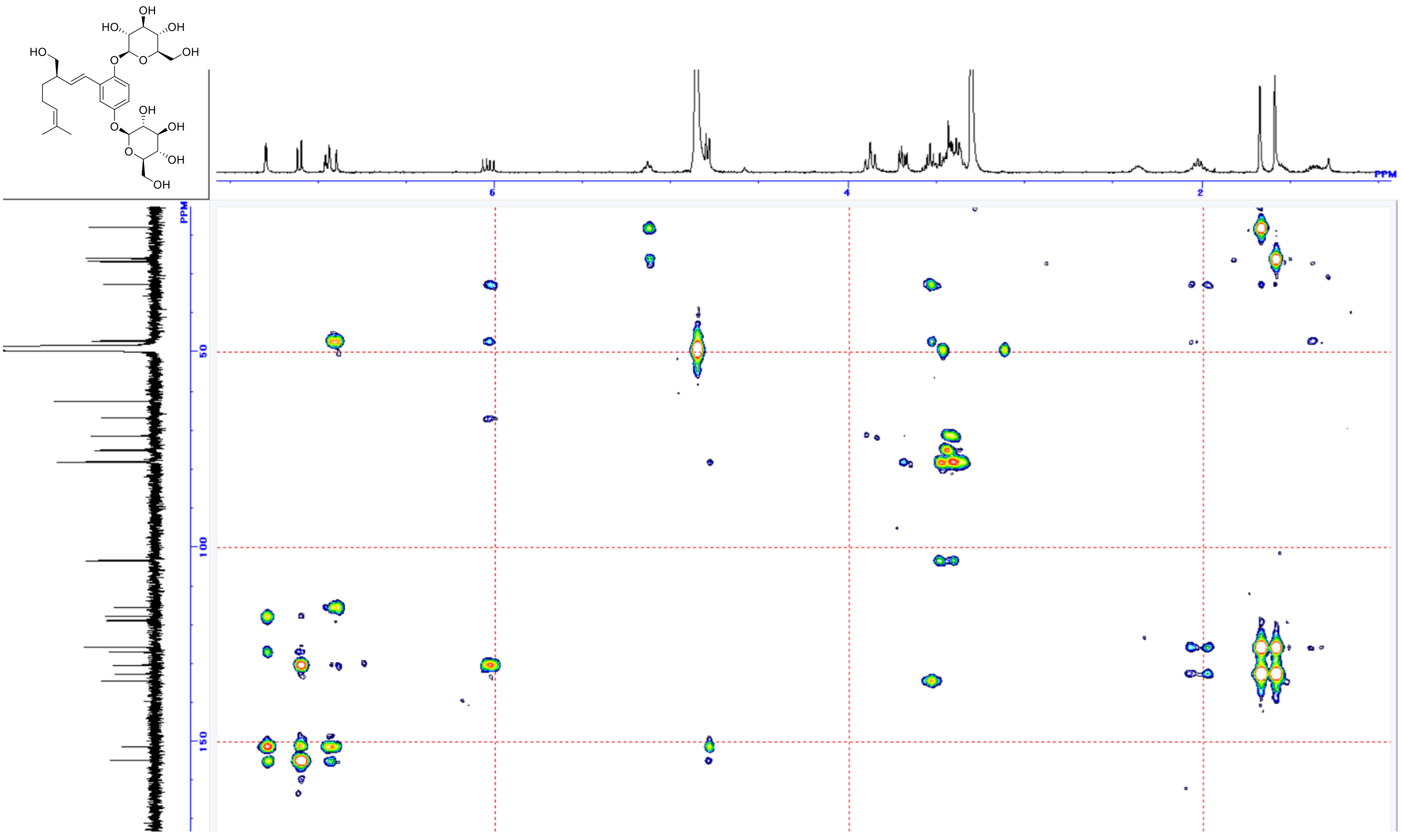

Figure S59 HMBC spectrum of compound **8** (in methanol-*d*4).

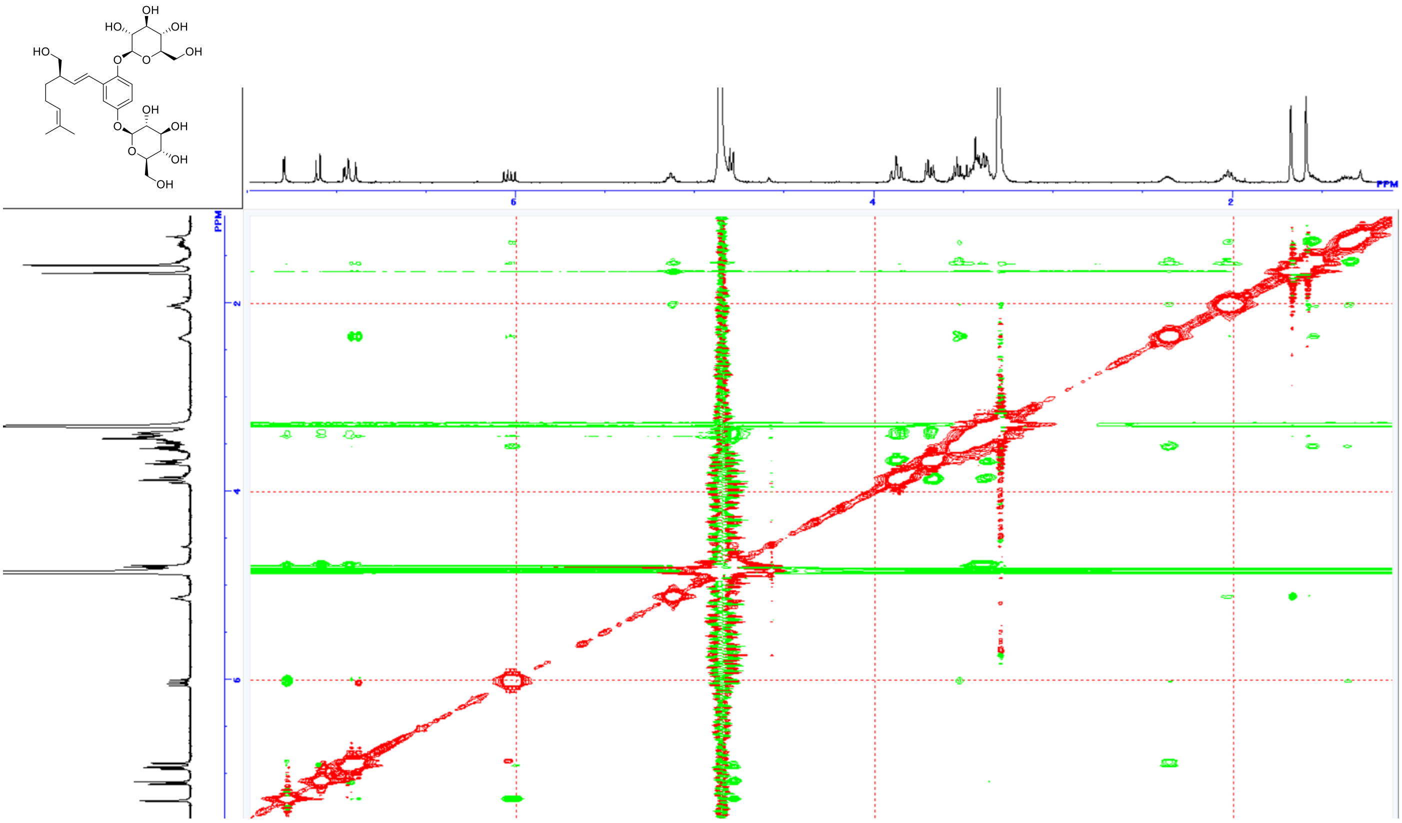

Figure S60 NOESY spectra of compound **8** (in methanol-d_4).

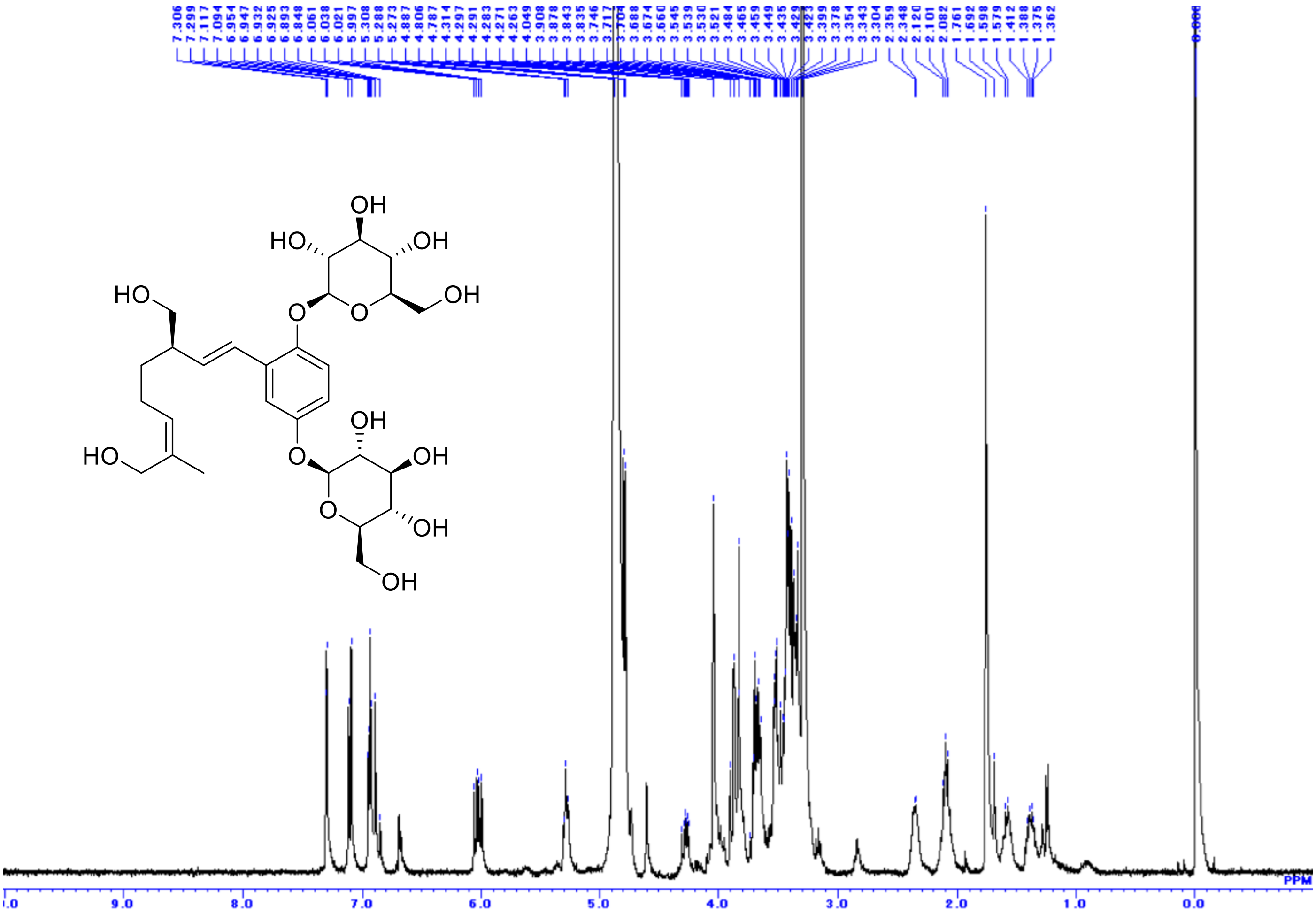

Figure S61 ^{1}H NMR spectrum of compound **9** (in methanol-d_4).

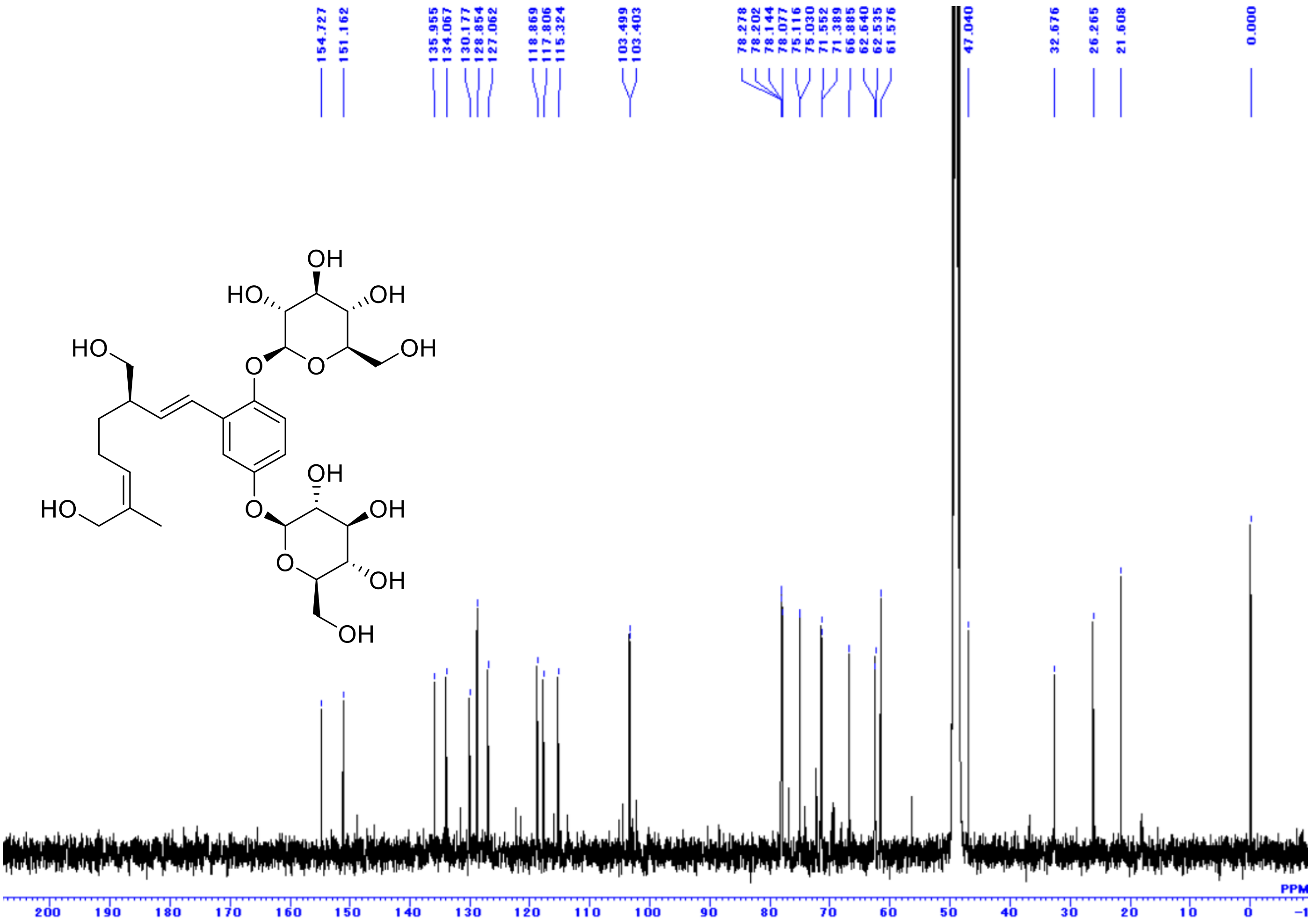

Figure S62 ^{13}C NMR spectrum of compound **9** (in methanol-d_4).

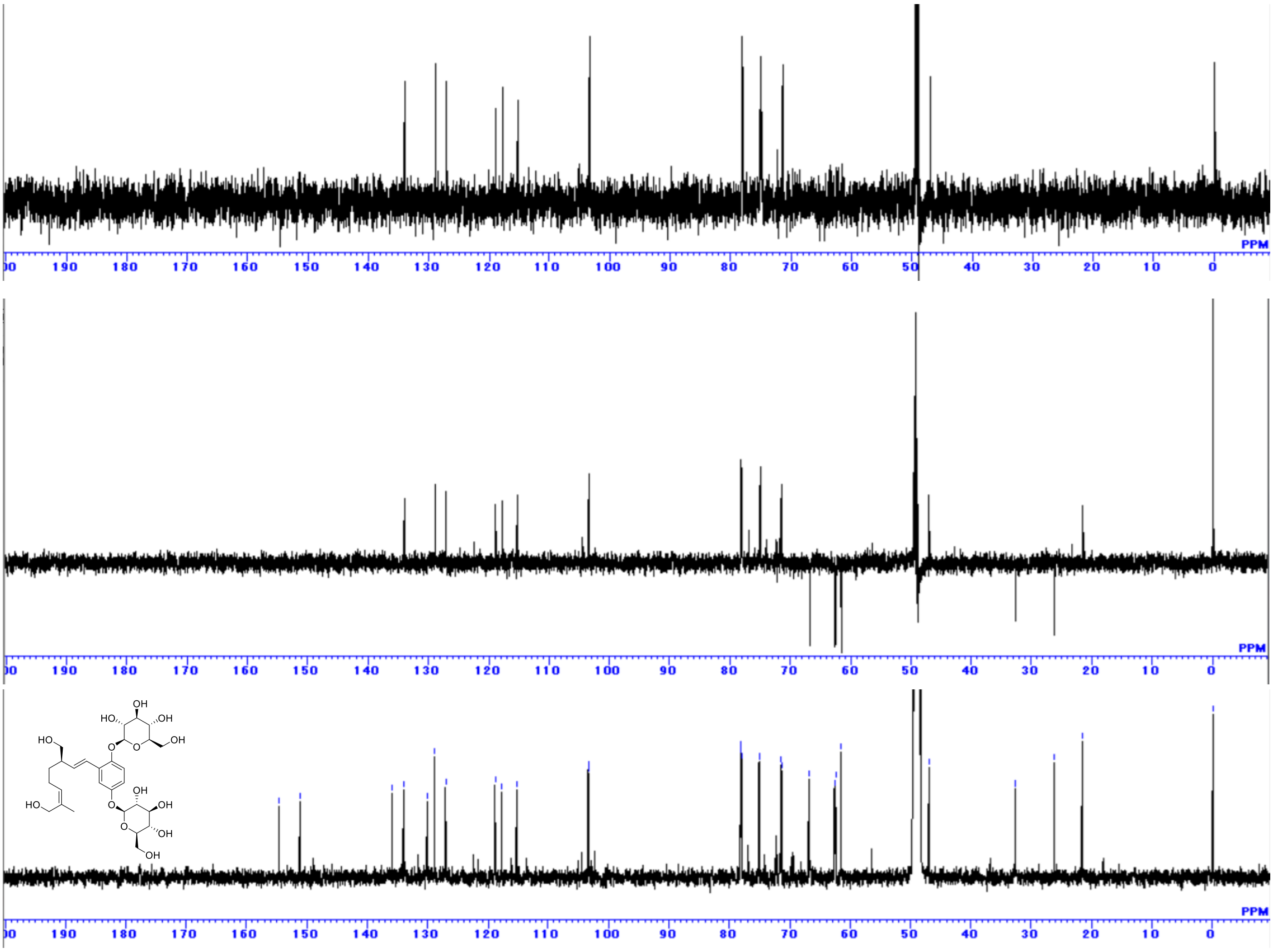

Figure S63 DEPT spectrum of compound **9** (in methanol-d_4).

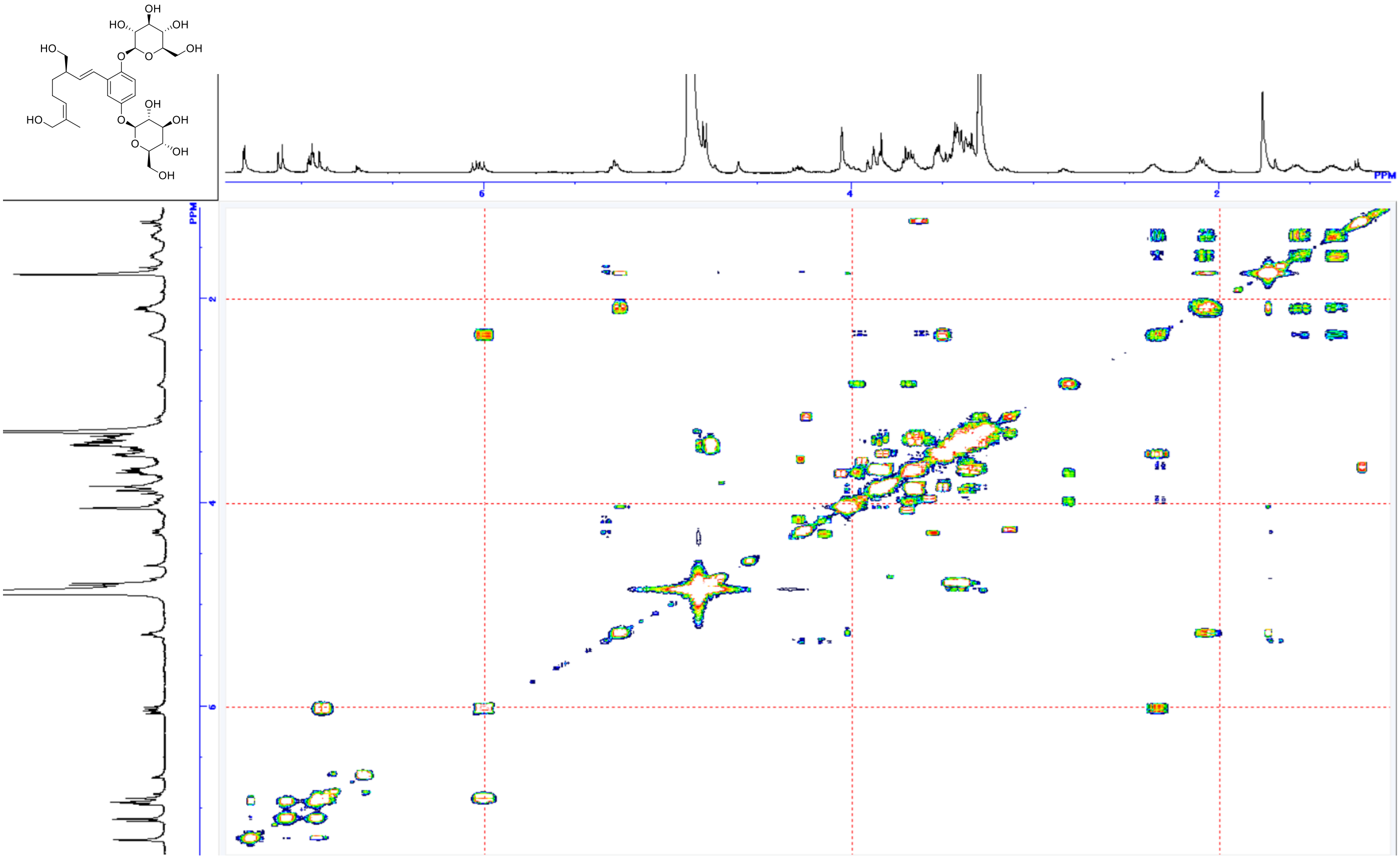

Figure S64 ^{1}H-^{1}H COSY spectrum of compound **9** (in methanol-*d*$_4$).

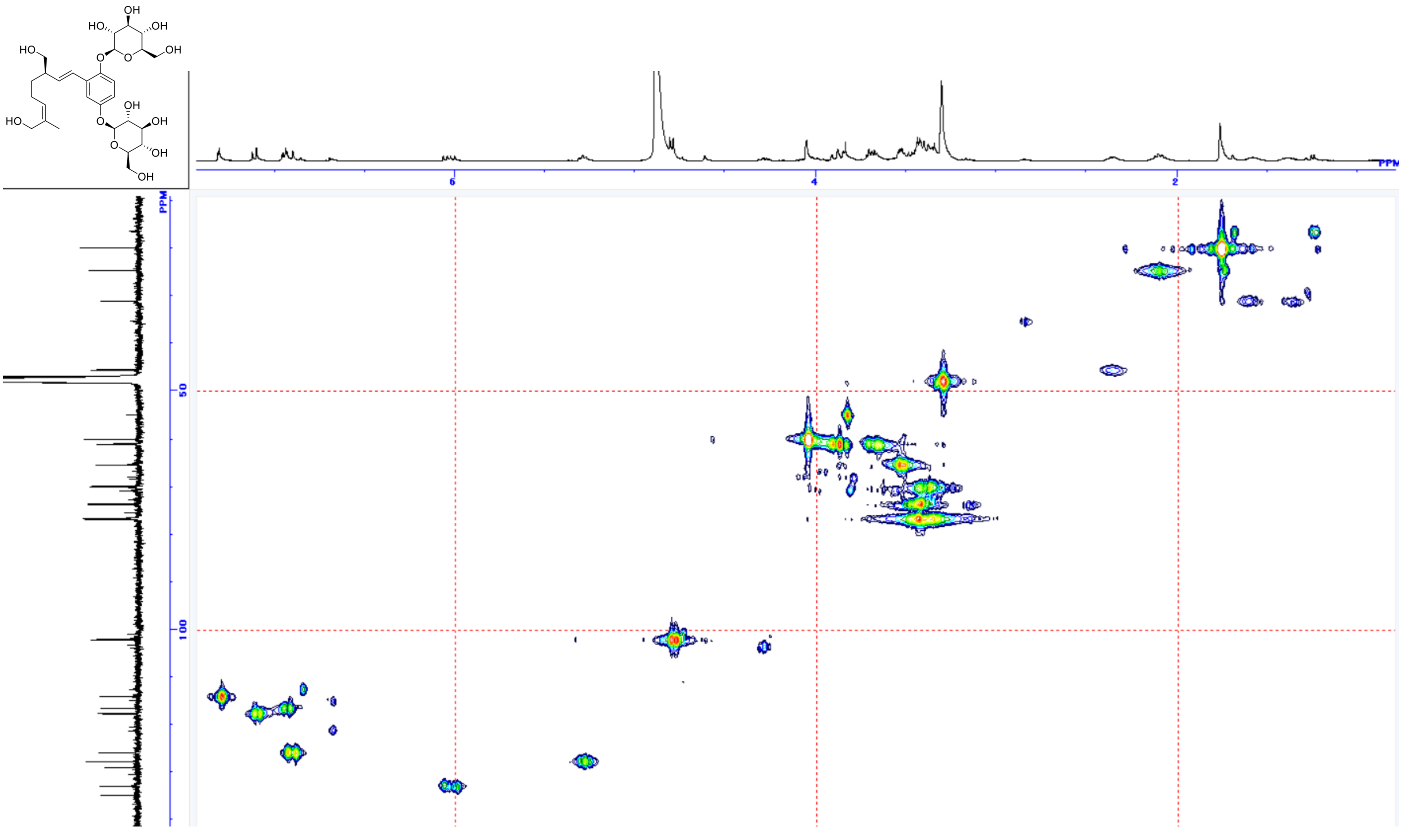

Figure S65 HMQC spectrum of compound **9** (in methanol-*d*$_4$).

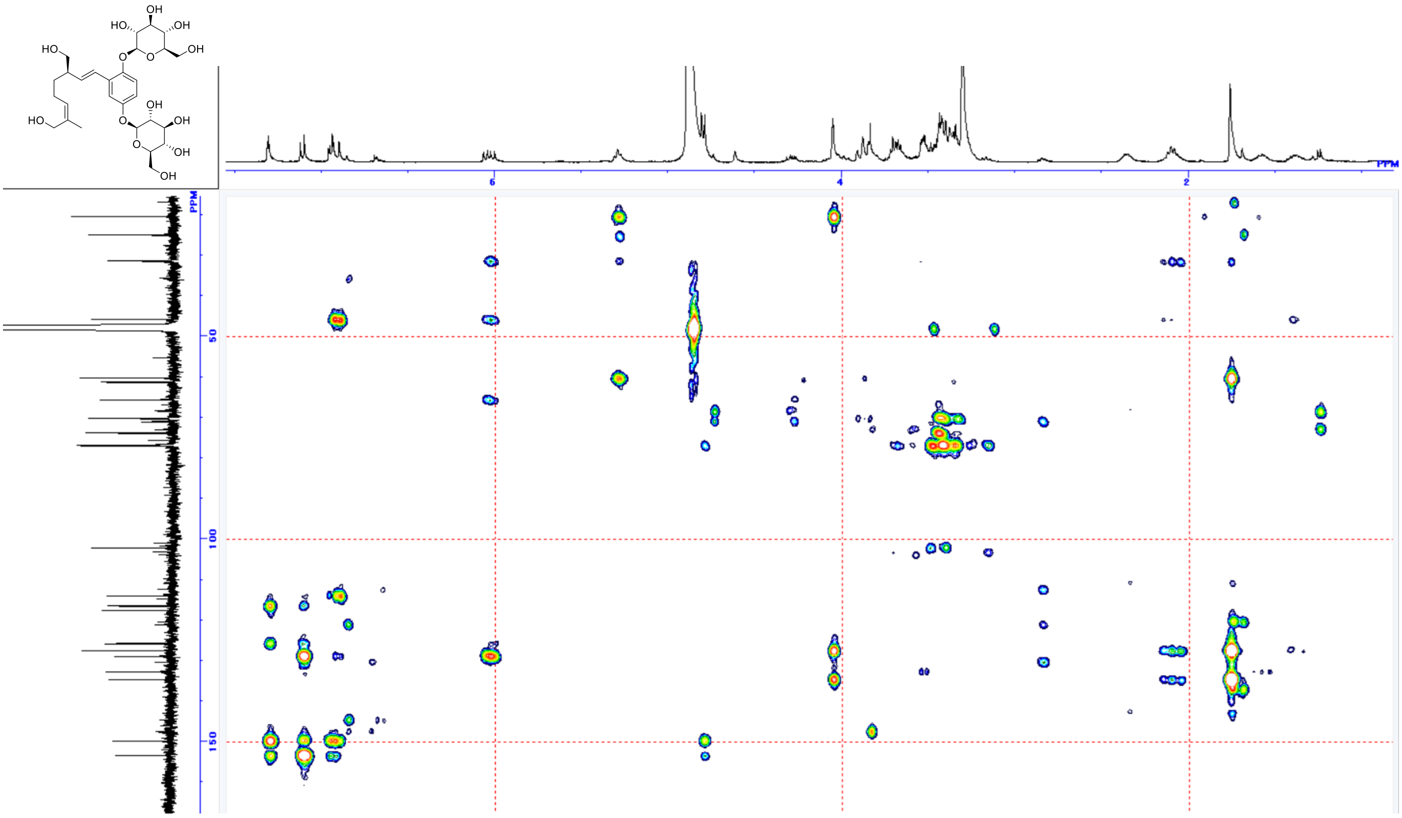

Figure S66 HMBC spectrum of compound **9** (in methanol-*d*4).

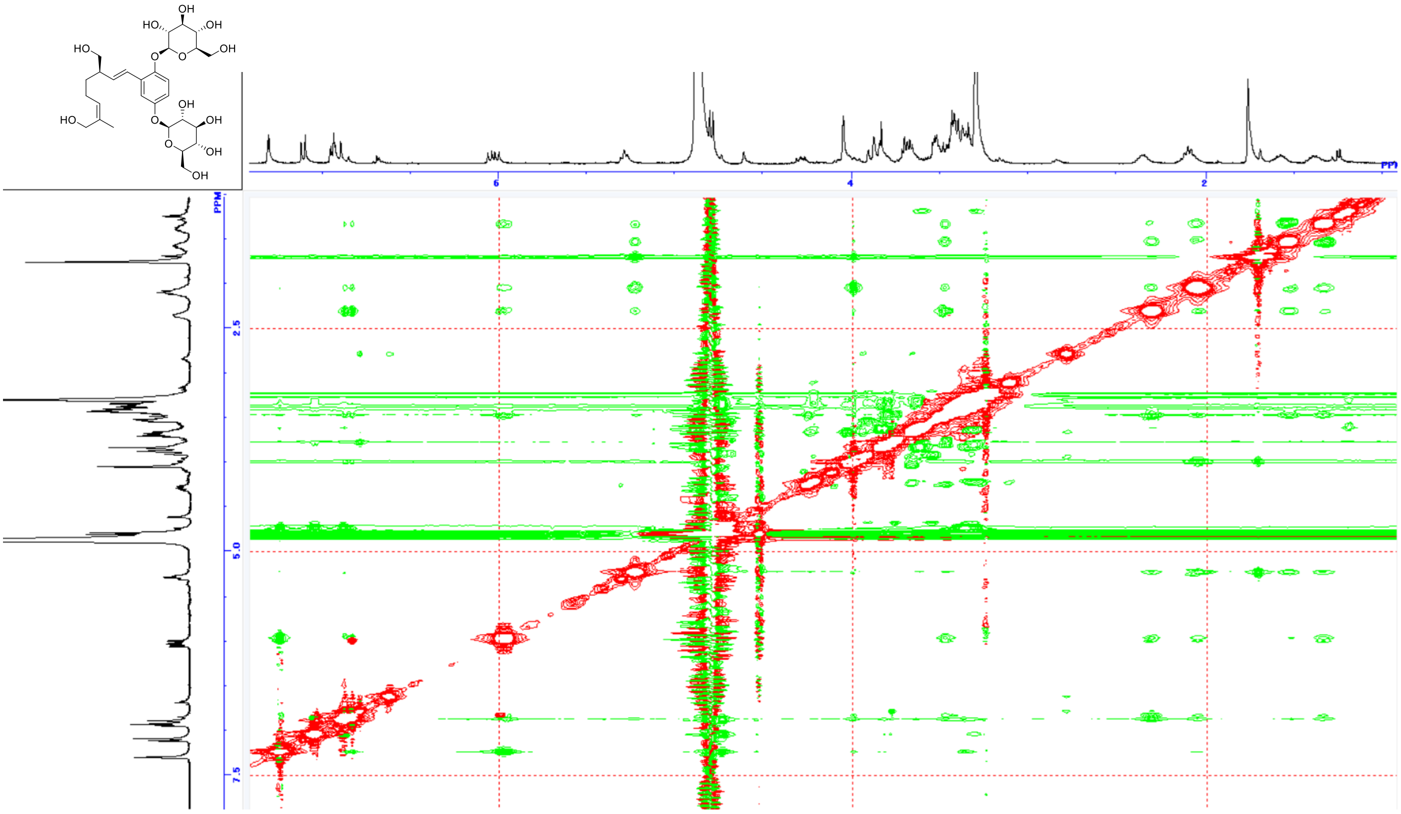

Figure S67 NOESY spectra of compound **9** (in methanol-*d*4).

Data : murata2024-05-17024 Date : 17-May-2024 11:21
Instrument : MStation
Sample : NM-31R (Glycerol)
Note : -
Inlet : Direct Ion Mode : FAB+
RT : 0.84 min Scan# : 11
Elements : C 28/0, H 36/0, O 13/0, Na 1/0
Mass Tolerance : 1000ppm, 5mmu if m/z < 5, 50mmu if m/z > 50
Unsaturation (U.S.) : -0.5 - 50.0

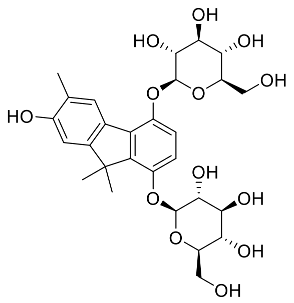

	Observed m/z	Int%	Err[ppm / mmu]	U.S.	Composition
1	603.2042	100.00	-1.9 / -1.2	10.5	C28 H36 O13 Na

Figure S68 HRFABMS of compound **1**.

Data : murata2024-05-21024 Date : 21-May-2024 11:14
Instrument : MStation
Sample : NM-31K (Glycerol) MW. 596
Note : -
Inlet : Direct Ion Mode : FAB-
RT : 2.42 min Scan# : 30
Elements : C 28/0, H 35/0, O 14/0
Mass Tolerance : 1000ppm, 5mmu if m/z < 5, 50mmu if m/z > 50
Unsaturation (U.S.) : -0.5 - 50.0

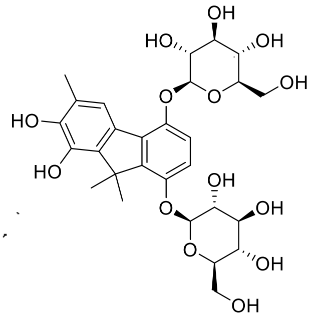

	Observed m/z	Int%	Err[ppm / mmu]	U.S.	Composition
1	595.2042	28.78	+2.6 / +1.5	11.5	C28 H35 O14

Figure S69 HRFABMS of compound **2**.

Data : murata2024-05-17023 Date : 17-May-2024 11:09
Instrument : MStation
Sample : NM-39F+15E (Glycerol)
Note : -
Inlet : Direct Ion Mode : FAB+
RT : 0.75 min Scan# : 10
Elements : C 28/0, H 42/0, O 13/0, Na 1/0
Mass Tolerance : 100mmu
Unsaturation (U.S.) : -0.5 - 50.0

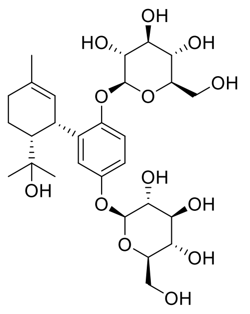

	Observed m/z	Int%	Err[ppm / mmu]	U.S.	Composition
1	609.2515	100.00	-1.3 / -0.8	7.5	C28 H42 O13 Na

Figure S70 HRFABMS of compound **3**.

Data : murata2024-05-21010 Date : 21-May-2024 09:16
Instrument : MStation
Sample : NM-16D (Glycerol)
Note : -
Inlet : Direct Ion Mode : FAB+
RT : 1.00 min Scan# : 13
Elements : C 28/0, H 42/0, O 13/0, Na 1/0
Mass Tolerance : 1000ppm, 5mmu if m/z < 5, 50mmu if m/z > 50
Unsaturation (U.S.) : -0.5 - 50.0

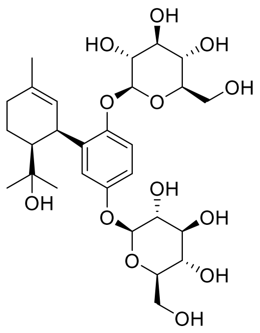

	Observed m/z	Int%	Err[ppm / mmu]	U.S.	Composition
1	609.2515	100.00	-1.3 / -0.8	7.5	C28 H42 O13 Na

Figure S71 HRFABMS of compound **4**.

Data : murata2024-05-21023 Date : 21-May-2024 11:02
Instrument : MStation
Sample : NM-20L (Glycerol) MW. 422
Note : -
Inlet : Direct Ion Mode : FAB-
RT : 0.67 min Scan# : 9
Elements : C 22/0, H 29/0, O 8/0
Mass Tolerance : 1000ppm, 5mmu if m/z < 5, 50mmu if m/z > 50
Unsaturation (U.S.) : -0.5 - 50.0

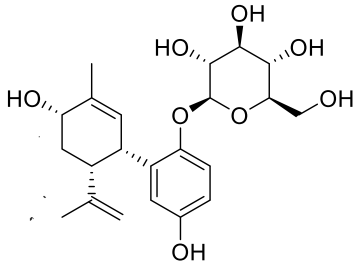

	Observed m/z	Int%	Err[ppm / mmu]	U.S.	Composition
1	421.1867	67.74	+1.1 / +0.5	8.5	C22 H29 O8

Figure S72 HRFABMS of compound **5**.

Data : murata2024-05-21022 Date : 21-May-2024 10:48
Instrument : MStation
Sample : NM-5L+J (Glycerol) MW. 600
Note : -
Inlet : Direct Ion Mode : FAB-
RT : 0.59 min Scan# : 8
Elements : C 28/0, H 39/0, O 14/0
Mass Tolerance : 1000ppm, 5mmu if m/z < 5, 50mmu if m/z > 50
Unsaturation (U.S.) : -0.5 - 50.0

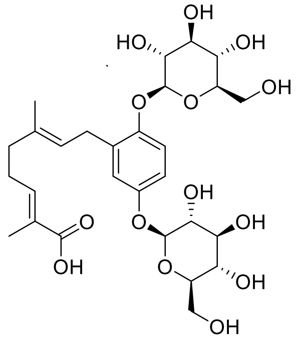

	Observed m/z	Int%	Err[ppm / mmu]	U.S.	Composition
1	599.2323	100.00	-2.8 / -1.7	9.5	C28 H39 O14

Figure S73 HRFABMS of compound **6**.

Data : kurosawa2024-10-04019 Date : 04-Oct-2024 10:26
Instrument : MStation
Sample : NM-65D (Glycerol) MW. 586
Note : -
Inlet : Direct Ion Mode : FAB-
RT : 2.09 min Scan# : 26
Elements : C 28/0, H 41/0, O 13/0
Mass Tolerance : 1000ppm, 5mmu if m/z < 5, 50mmu if m/z > 50
Unsaturation (U.S.) : -0.5 - 50.0

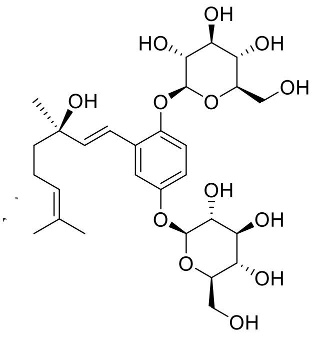

	Observed m/z	Int%	Err[ppm / mmu]	U.S.	Composition
1	585.2535	100.00	-2.1 / -1.2	8.5	C28 H41 O13

Figure S74 HRFABMS of compound **7**.

Data : kurosawa2024-10-04018 Date : 04-Oct-2024 10:06
Instrument : MStation
Sample : NM-66B (Glycerol) MW. 586
Note : -
Inlet : Direct Ion Mode : FAB-
RT : 2.25 min Scan# : 28
Elements : C 28/0, H 41/0, O 13/0
Mass Tolerance : 1000ppm, 5mmu if m/z < 5, 50mmu if m/z > 50
Unsaturation (U.S.) : -0.5 - 50.0

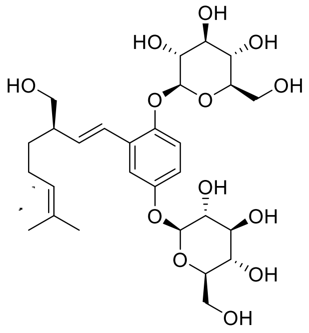

	Observed m/z	Int%	Err[ppm / mmu]	U.S.	Composition
1	585.2540	100.00	-1.2 / -0.7	8.5	C28 H41 O13

Figure S75 HRFABMS of compound **8**.

Data : kurosawa2024-10-04020 Date : 04-Oct-2024 10:42
Instrument : MStation
Sample : NM-67B (Glycerol) MW. 602
Note : -
Inlet : Direct Ion Mode : FAB-
RT : 2.34 min Scan# : 29
Elements : C 28/0, H 41/0, O 14/0
Mass Tolerance : 1000ppm, 5mmu if m/z < 5, 50mmu if m/z > 50
Unsaturation (U.S.) : -0.5 - 50.0

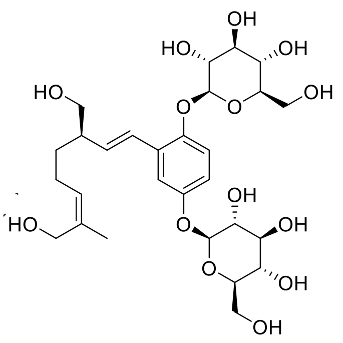

	Observed m/z	Int%	Err[ppm / mmu]	U.S.	Composition
1	601.2493	51.27	-0.6 / -0.3	8.5	C28 H41 O14

Figure S76 HRFABMS of compound **9**.

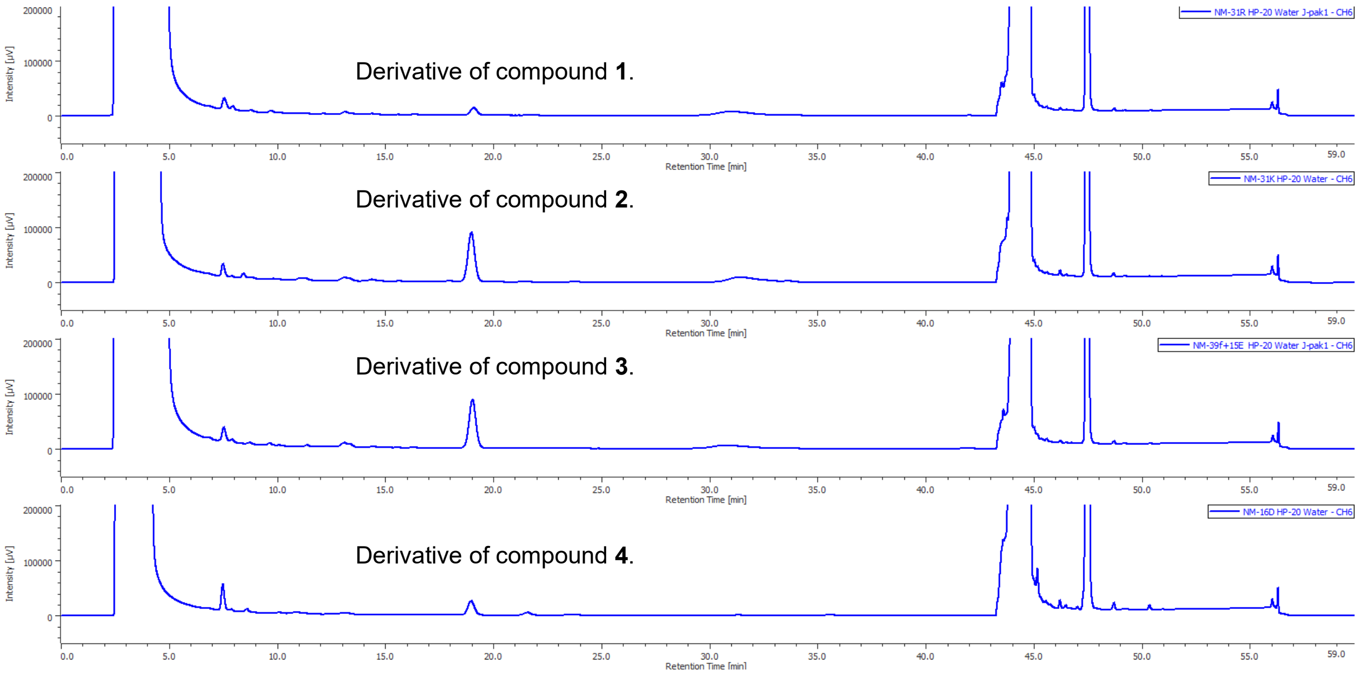

Figure S77 HPLC analyses for sugar identification of **1-4. f**

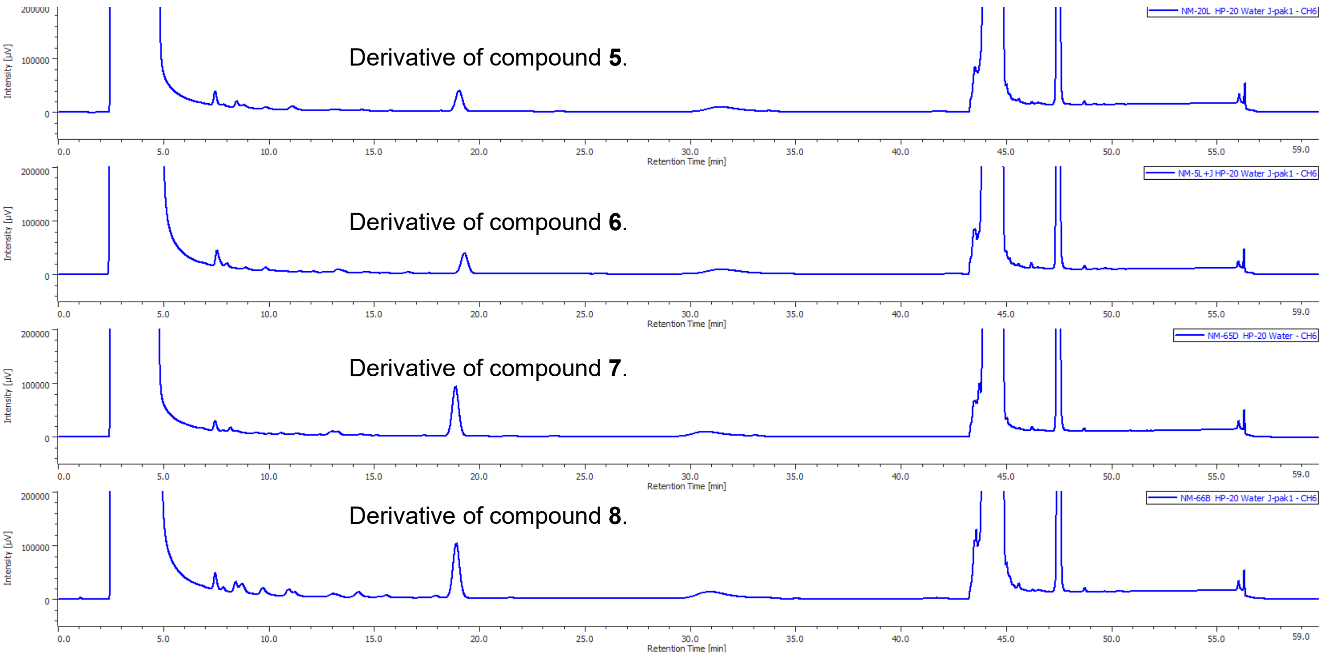

Figure S78 HPLC analyses for sugar identification of **5-8**.

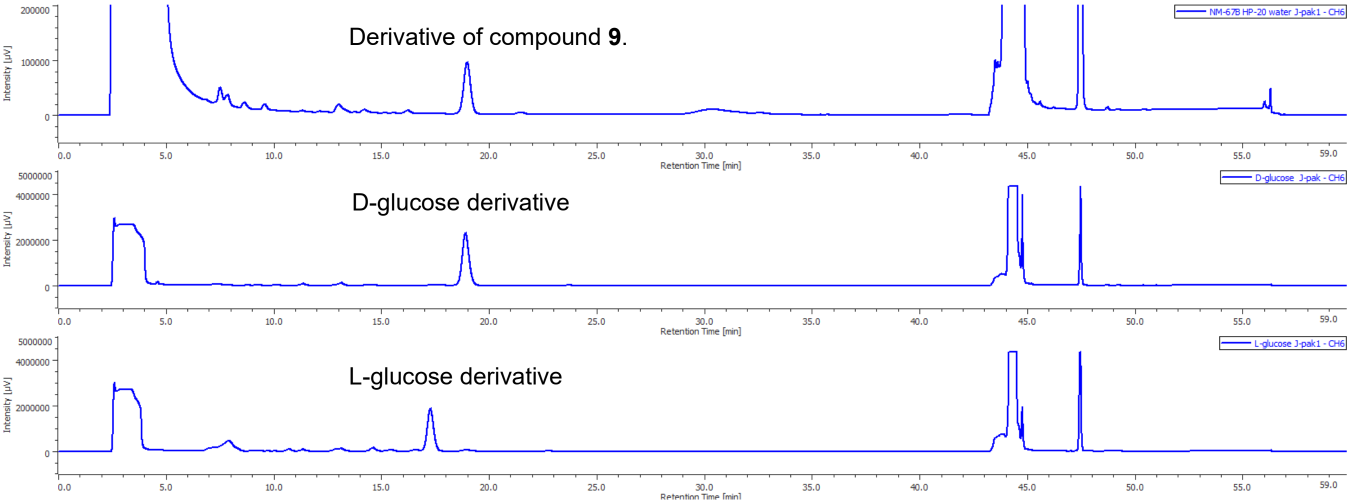

Figure S79 HPLC analyses for sugar identification of **9**.
